# Mechanochemically Synthesized Amidetriazoles for Effective Optical and Electrochemical Detection of Anions

**DOI:** 10.1021/acs.joc.5c03014

**Published:** 2026-03-27

**Authors:** Jakub S. Cyniak, Daria Szeląg, Róża Sitek, Wojciech Wróblewski, Artur Kasprzak

**Affiliations:** Faculty of Chemistry, 201870 Warsaw University of Technology, Noakowskiego Str. 3, Warsaw 00-664, Poland

## Abstract

Despite the fact that amide and 1,2,3-triazole moieties on their own are widely utilized in designing chemoreceptor structures, their direct connection—amidetriazole is rarely studied. In this work, we present a new class of polyaromatic AIE-active amidetriazoles, their mechanochemical synthesis, and their application as dual-mode anion receptors. Mechanochemical synthesis not only enabled high yields but also shortened the reaction time and reduced the use of solvents and harmful chemicals. The optimization of 1,3-dipolar cycloaddition reactions carried out in solution, as well as in sono- and mechanochemical versions, was mainly based on modifying the copper-ion source, where a nonclassical approach (copper metal mesh) enabled the highest yields to be achieved. The compounds obtained were characterized by spectroscopic methods to fully study their properties derived from the AIE effect. The relationship between their structure and receptor properties was outlined. Ultimately, both AIE-active compounds were successfully employed as anion receptors. Optical and potentiometric methods demonstrated a strong correlation between the receptor structure and its detection properties, as well as the selective interaction of one receptor with SO_4_^2–^ anions.

## Graphical abstract

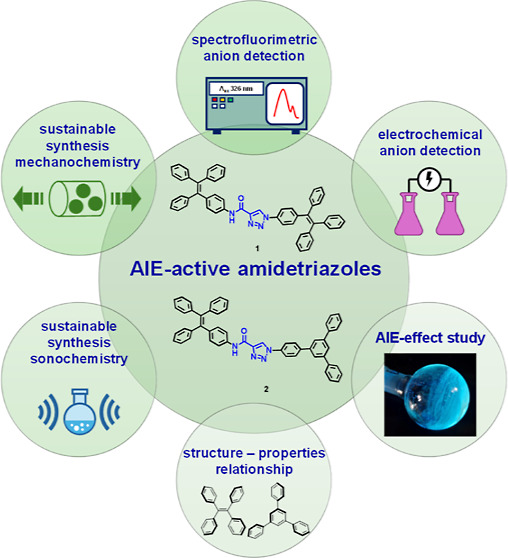

## Introduction

Over the past two decades, the chemistry of molecular receptors has undergone a revolution with the discovery of the aggregation-induced emission (AIE)^1^ effect in 2001.^2^ This effect is observed for nonplanar polyaromatic compounds that contain aromatic rings that can rotate in solution (no light emission). In contrast, in the aggregated or solid state, these compounds are very effective luminophores. This effect has found a wide range of applications in the design of new functional materials,^3–8^ including molecular receptors exhibiting the AIE effect.^9–21^ A characteristic feature of AIE-active chemoreceptors is a significant change in light emission intensity upon binding the analyte, which enhances the overall sensitivity of the method and mitigates the impact of interference from concentration fluctuations. Another important feature is the ability to use water-insoluble compounds for detecting analytes in organic solvent–water systems that contain a significant volume percentage of water (typically above 90% by volume). This is particularly favorable for analyzing real samples. To date, there are no examples in the literature of AIE-active chemoreceptors containing an amidetriazole motif.

Additionally, as the scientific community becomes increasingly aware of the negative environmental impact of chemical synthesis, attention is being paid to minimizing it. This can be achieved by employing nonclassical reaction techniques to minimize the amount of reagents and solvents used and by conducting the reactions in a manner that limits the amount of generated byproducts. Among these methods, sono- ^22–25^ and mechanochemistry^26–29^ are considered the leading methods. Both sonochemistry, where the energy required for a reaction to take place is supplied by high-frequency ultrasonic waves, and mechanochemistry, where the energy is supplied by the direct absorption of mechanical energy, have been gaining popularity in recent years due to their ease of implementation, almost complete elimination of solvents from chemical synthesis while allowing excellent yields to be obtained in less time as well as, in some cases, products that are unobtainable by classical methods.^30–37^ All this makes mechanochemistry increasingly widely used in the synthesis of luminescent materials,^38^ chemoreceptors,^30,36,39^ Active Pharmaceutical Ingredients (APIs),^40,41^ and porous materials.^42–44^ Sonochemistry is increasingly utilized in the synthesis of nanomaterials,^45^ heterocyclic compounds,^46^ amides,^47^ and photocatalysts.^48^

The amide bond and the 1,2,3-triazole moiety are both widely used in molecular receptor design^49–58^ due to their facile, diverse synthesis methods that utilize readily available substrates, mild reaction conditions, and high yields. This approach also provides effective noncovalent binding phenomena through electrostatic forces and hydrogen-bonding interactions. Despite these advantages, the direct combination of these two structural motifs in a single molecule, in which the amide moiety is directly linked to the 4-position of the triazole ring, remains a rare approach. Only a small number of papers describing the use of the amidetriazole motif have been published in the last 15 years.^59^ Most of the works concern biologically active molecules (Figure 1a–e) that act as macrophage migration inhibitory factors (MIF),^60,61^ cytostatic drug,^62,63^ or in the treatment of Alzheimer’s disease.^64^ An example of the use of an amidetriazole derivative as an auxiliary ligand in olefin polymerization catalysts (Figure 1f) is also known.^65^ The formation of a 1,4-dinitrogen system via an amidetriazole moiety makes it valuable in molecular receptor chemistry. More than a decade ago, a group of Y.-J. Li presented a series of foldamers (Figure 1g–j), and multicentered chemoreceptors (Figure 1k,l) containing up to 3 amidetriazole units per molecule.^66,67^ Presented compounds have been studied regarding their interaction with halide anions and oxyanions (NO_3_^–^, AcO^–^, H_2_PO_4_^–^, and SO_4_^2–^) by ^1^H NMR spectroscopy only. All compounds exhibited relatively low binding constants (*K*_1_ < 10^2^) for halide anions, showing a noticeably higher affinity toward oxyanions. The highest *K* values were obtained for the interaction with SO_4_^2–^ for foldamer shown in Figure 1i (R = H)—at the level of 10^3^ [M^–1^] and for the compound containing three amidetriazole moieties >10^5^ [M^–1^] (Figure 1l). UV–vis titrations were also carried out for this compound, where absorption quenching was observed with increasing anion concentrations in the sample. From this, the stoichiometry of the complex was determined at 1:1 (by Job’s plot).

**Figure 1:**
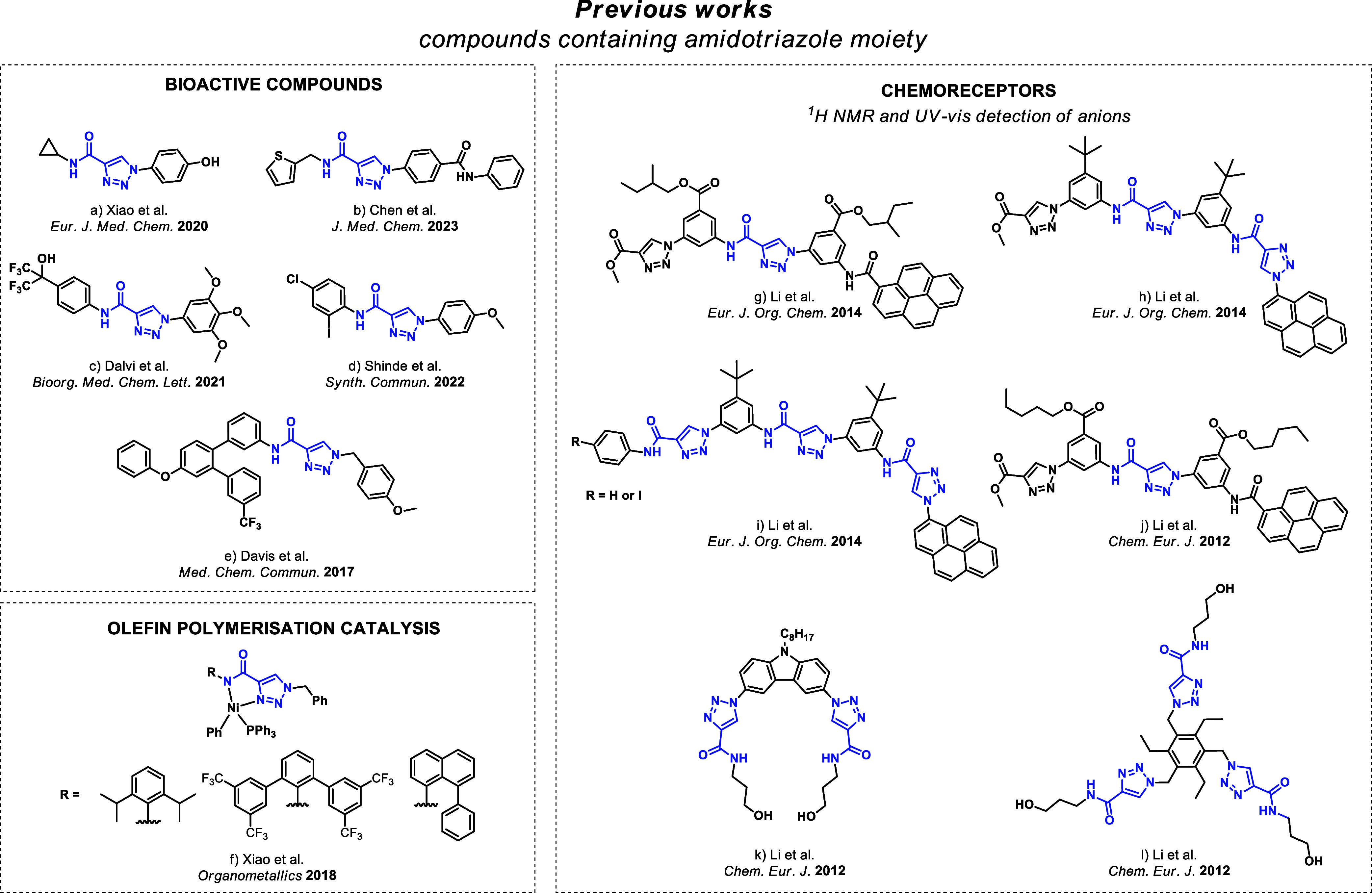
Graphical representation of the state-of-the-art regarding amidetriazoles: (a–e) bioactive compounds, (f) catalysts, (g–l) chemoreceptors.^60–67^

To date, there are no examples in the literature of AIE-active chemoreceptors containing an amidetriazole motif. In this work, we present the design and synthesis of novel polyaromatic AIE-active amidetriazole-bearing 1,1,2,2-tetraphenylethene (TPE) and 1,3,5-triphenylbenzene (TPB) skeletons (compounds **1** and **2**, Figure 2). The application of nonclassical reaction conditions (sono- and mechanochemistry) allowed us to achieve yields higher than those obtained in a solution-based approach. Properties derived from the AIE effect were thoroughly studied. Finally, we successfully applied the obtained compounds as both optical (spectrofluorimetry) and, for the first time in the history of AIE-active chemoreceptors, electrochemical (potentiometry) receptors for the detection of inorganic anions. The innovative application of AIE-active polyaromatic amidetriazoles in the construction of potentiometric sensors, in conjunction with good agreement with the outcomes from optical receptor studies, opens up exciting new possibilities in the chemistry of AIE-active chemoreceptors.^68^

**Figure 2:**
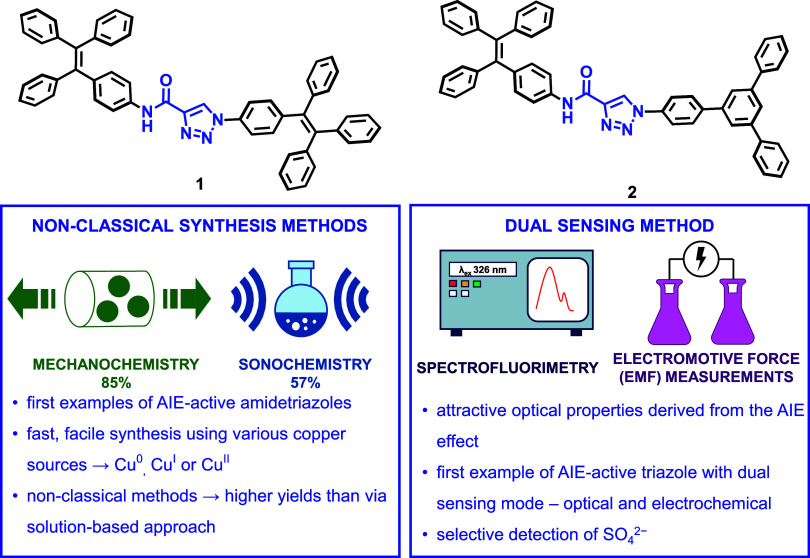
Key compounds obtained in this work with the outline of the results.

## Results and Discussion

### Synthesis

Refer to Supporting Information, Section S1, for the full experimental details. The syntheses of target molecules **1** and **2** are presented in Scheme 1. First, polyaromatic azides **4** and **7** were synthesized.^69^ The starting materials, namely 4-(1,2,2-triphenylvinyl)­aniline (**3**) and 5′-phenyl-[1,1’:3′,1″-terphenyl]-4-amine (**6**), were first reacted with sodium nitrite in acidic aqueous solution to obtain respective diazonium salts, which were then reacted with excess sodium azide to form azides **4** and **7** with 96% and 93% yields, respectively (Scheme 1 *i*, *iii*). Remarkably, the synthesis of azide **7**, in contrast to most protocols, was carried out entirely at room temperature. Optimization experiments (see Supporting Information, Sections S1.2.3 and S1.2.5) showed that the first step of the reaction, the synthesis of the diazonium salt at reduced temperature, results in lower yields. A 4-fold excess of sodium azide proved to be necessary in this case to convert the entire diazonium salt into the azide. Despite the similar structure, azide **4** required a reduced temperature for the diazonium salt synthesis step to obtain a high yield. Amide **5** was obtained in high yield (80%) (Scheme 1 (ii) by direct reaction of amine **3** with propiolic acid in the presence of the coupling agent *N,N′*-dicyclohexylcarbodiimide (DCC) and *N*-hydroxysuccinimide (NHS) in dichloromethane (DCM). The possibility of synthesizing amide **5** via a mechanochemical reaction was also demonstrated by grinding the substrates in the presence of DCC and NHS, with 15 μL of dichloromethane (LAG, Liquid Assisted Grinding) for 3 h at 30 Hz. This method allowed only a moderate yield (36%) to be achieved. The target derivatives **1** and **2** were synthesized in the direct reaction of azides **4** and **7**, respectively, with amide **5** via a copper-catalyzed 1,3-dipolar cycloaddition (*click chemistry* approach)^70^ in three variants: in solution, mechanochemically, and sonochemically (Scheme 1 *iv, v*). For the synthesis in solution of compound **1**, the catalyst system consisted of copper­(I) thiophene-2-carboxylate (CuTC) with *N,N*-diisopropylethylamine (DIPEA), while for compound **2**, copper­(I) ions were generated in situ by the reaction of copper sulfate pentahydrate (CuSO_4_·5H_2_O) with sodium l-ascorbate. In both cases, the reactions were carried out for 48 h in dimethylformamide (DMF) to give compounds **1** and **2** with yields of 52% and 56%, respectively.

**Scheme 1:**
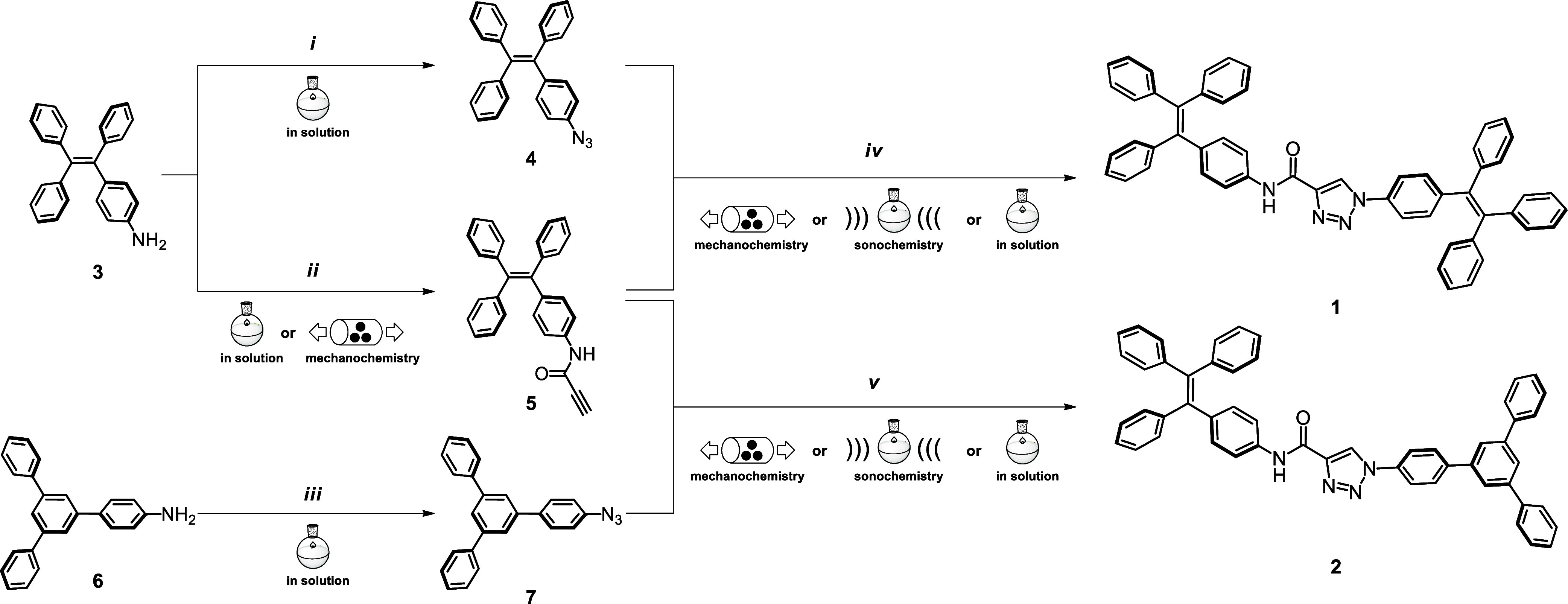
Synthesis of Compounds **1**-**2** and Their Precursors **4**, **5**, **7**; Conditions: (**i**) in Solution: NaNO_2_, NaN_3_, HCl_aq_, 4 h, 0 °C → RT, 96%; (**ii**) in Solution: Propiolic Acid, DCC, NHS, DCM, 48 h, RT, 80%; Mechanochemistry (Ball Mill): Propiolic Acid, DCC, NHS, DCM (LAG), 30 Hz, 3 h, 36%; (**iii**) in Solution: NaNO_2_, NaN_3_, HCl_aq_, 4 h, RT, 93%; (**iv**) in Solution: CuTC, DIPEA, DMF, 48 h, RT, 52%; Mechanochemistry (Ball Mill): Copper Metal Mesh (>99,95% Cu), Sodium l-Ascorbate, DCM (LAG), 8 h, RT, 85%; Sonochemistry: Copper Metal Mesh (>99,95% Cu), Sodium l-Ascorbate, DCM, 6 h, RT, 60%; (**v**) in Solution: CuSO_4_·H_2_O, Sodium l-Ascorbate, DMF, 48 h, RT, 56%; Mechanochemistry (Ball Mill): Copper Metal Mesh (>99,95% Cu), Sodium l-Ascorbate, DCM (LAG), 6 h, RT, 50%; Sonochemistry: Copper Metal Mesh (>99,95% Cu), Sodium l-Ascorbate, DCM (LAG), 6 h, RT, 40%

To the best of our knowledge, only a limited number of examples of 1,3-dipolar cycloaddition (click chemistry) reactions performed mechanochemically are reported in the literature.^71–73^ This method demonstrated excellent yields of products within a short time with minimal byproduct formation. However, those examples mostly concern reactions of simple alkyl and aryl substrates and are catalyzed by copper­(I)^74,75^ and copper­(II) salts,^76^ or copper­(II) supported on alumina.^77^ In this work, we conducted reactions with different sources of copper ions. Copper­(I) ions were generated in situ using a reducing agent (sodium l-ascorbate) from copper­(II) salts: copper­(II) sulfate pentahydrate (CuSO_4_·5H_2_O) and copper­(II) acetate (Cu­(OAc)_2_) or copper­(I) salts: copper­(I) iodide (CuI) and copper­(I) thiophene-2-carboxylate (CuTC) were used directly (with addition of DIPEA). Reagents were ground for 3 h with a frequency of 30 Hz in the presence of 25 μL of DCM (LAG^78,79^ ) or without additional solvent. The highest obtained yield was 33% for the reaction with Cu­(OAc)_2_. Then we investigated the possibility of using elemental copper (Cu(0)) instead of copper­(I) or copper­(II) salts. Such reactions are also known in the literature;^80–84^ however, these usually concern reactions carried out in solution (flow systems) at high temperatures, of the order of 100–150 °C, which are necessary due to the low activity of copper(0). Mechanochemical syntheses using copper(0) are sparse and involve grinding in a copper vessel with a copper ball(s),^85–87^ the use of metallic copper powder,^88,89^ or the Resonant Acoustic Mixing (RAM) technique;^90^ however, they allow very good yields to be achieved. In our study, we chose to use copper metal mesh (>99.95% Cu) as a catalyst (see Supporting Information, Section S1.2.1 for the full experimental details). Eight times the weight of amide **5** of copper metal mesh was used in each reaction. Grinding the substrates in the presence of a copper metal mesh for 3 h yielded 11%; the addition of sodium l-ascorbate more than tripled the yield to 35% after the same time. Interestingly, after grinding without the reducing agent, the metal mesh was broken into only small pieces. In contrast, the addition of the reducing agent caused the mesh not only to disintegrate but also the mesh pieces to be visibly etched. Extending the reaction time to 6 h resulted in a further increase in yield to 70% and finally, after 8 h, to 85%.

The sonochemical approach also proved to be an effective method of synthesizing compounds **1** and **2**; however, the obtained yields were slightly lower than those in the mechanochemical method. In the case of compound **1**, reactions with a copper metal mesh (in the presence of sodium l-ascorbate) and reactions with CuTC and DIPEA allowed similar yields to be achieved (60% and 57%, respectively). For compound **2**, a 40% yield was achieved in reaction with a copper metal mesh.

To compare the efficiency of the methods used to synthesize compound **1** (in solution, sonochemically, and mechanochemically), the syntheses yields obtained by these methods were compared for three sets of parameters covering different copper sources (Cu­(I), Cu­(II), and Cu(0)), used additives and solvent (Table 1). Overall, synthesis in solution provided the lowest yields for each of the three parameters and required the largest volume of solvent and the longest reaction time. Sonochemical synthesis using copper­(II) yielded results similar to those obtained with the solution method while consuming 40 times less solvent in 8-fold less time. This approach yielded similar results for syntheses using copper­(I) and copper(0). The best results were obtained for each of the three sets of parameters using mechanochemistry, while consuming the lowest solvent volume (80 times less than in synthesis in solution) and in a time comparable to that of sonochemical syntheses. Of particular importance here is the synthesis using a copper mesh, which not only eliminates the need for DIPEA (hazard identification: see SI, section S1.2) but also ensures the highest yield. The structure of the compounds **1** and **2** was confirmed by ^1^H NMR and {^1^H}^13^C NMR spectroscopies and high-resolution mass spectrometry (HRMS) (refer to Supporting Information, Sections S2 and S3, for the full details).

**Table 1:**
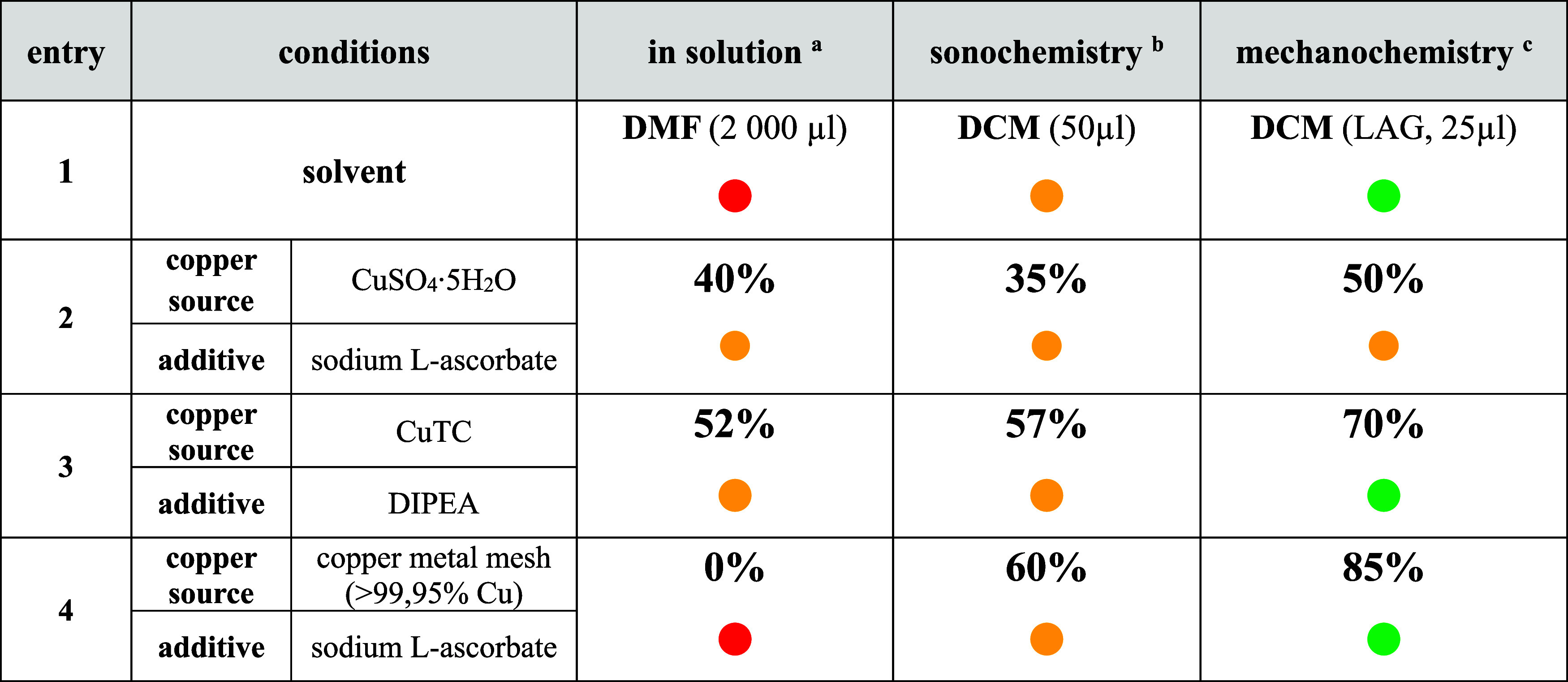
Summary of Optimization Experiments for the Synthesis of Compound **1** Using the Solution Method, Sonochemistry, and Mechanochemistry in Three Sets of Parameters

a Reaction time −48h.

b Reaction time – 6h.

c Reaction time – 8h.

### Analyses of AIE Properties of 1 and 2

The fundamental photophysical properties of **1** and **2** were studied by means of UV–vis and fluorescence spectroscopies. Full spectroscopic data are presented in Supporting Information, and Section S5. The UV–vis spectrum of **1** features three major absorption maxima (λ_max_) located at 218, 244, and 326 nm, while for compound **2**, only two absorption maxima (λ_max_) located at 218 and 266 nm were observed. Interestingly, the **2** featured molar absorption coefficient (ε) value is almost twice as high as that of **1**, at the level of 6.3·10^4^ dm^3^·mol^–1^·cm^–1^. Both compounds were found to be violet light emitters in the dissolved state, as their fluorescence spectra featured emission maxima (λ_em_) between 375 and 400 nm (Figure 3a).

**Figure 3:**
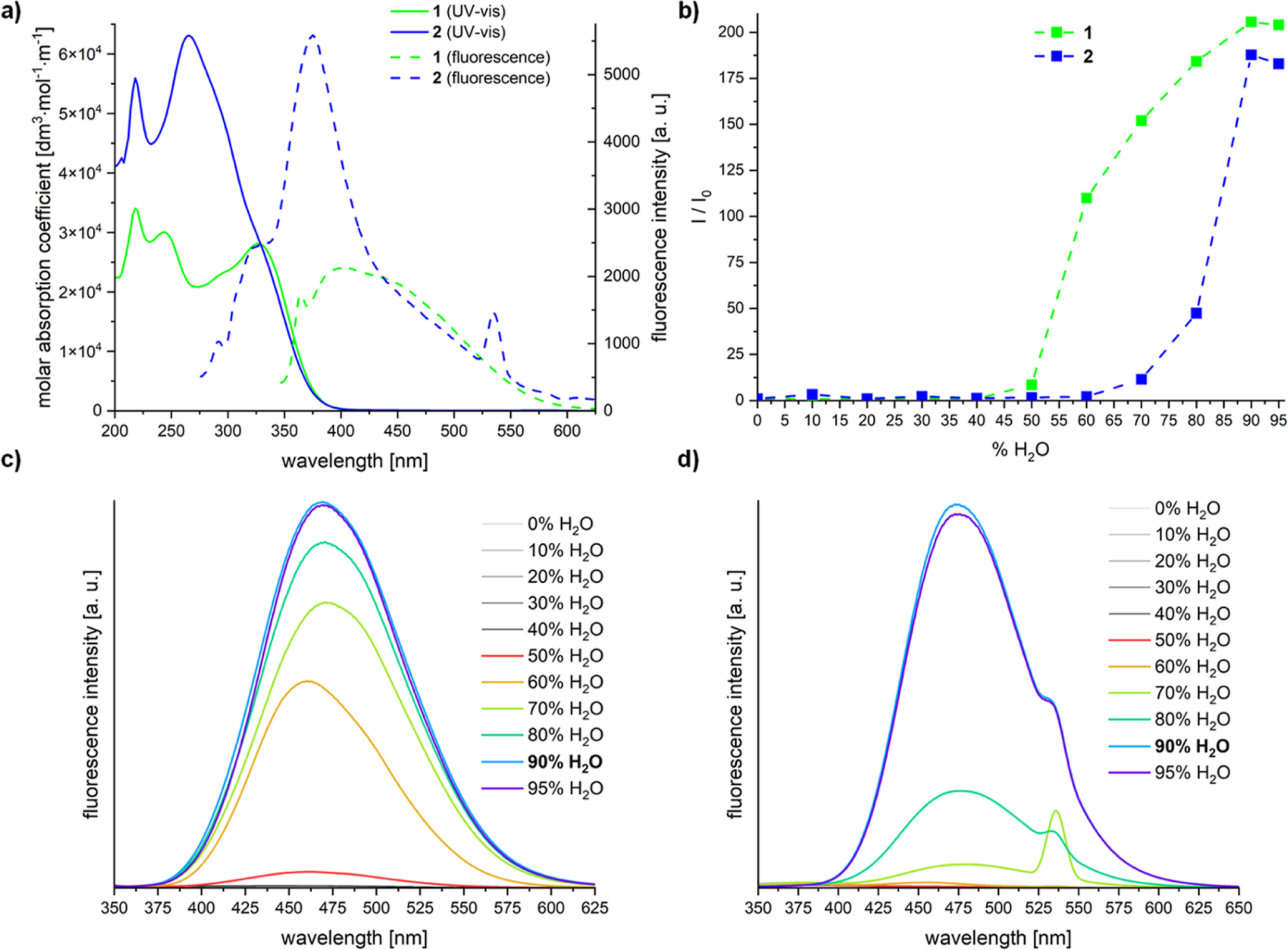
(a) UV–vis (*C*_1,2_ = 2·10^–5^ M, THF) and fluorescence spectra, experimental conditions: (*C*_1,2_ = 2·10^–5^ M, THF, λ_ex,1_ = 326 nm, λ_ex,2_ = 266 nm); (b) fluorescence intensity dependence for 1 and 2 on the H_2_O content (vol %) in the sample; (c) fluorescence spectra of 1 in different H_2_O/THF solvent systems (λ_ex_ = 326 nm); and (d) fluorescence spectra of 2 in different H_2_O/THF solvent systems (λ_ex_ = 266 nm).

In the next step, to check AIE behavior for these compounds, fluorescence spectra in mixtures of THF (good solvent) and H_2_O (bad solvent) were measured, with increasing vol % of H_2_O in the sample (Figure 3b–d). Both compounds exhibited exceptional AIE behavior. Compound **1** exhibited minimal fluorescence intensity up to 50% of that of water in the sample. For higher vol % of H_2_O, a sharp increase of fluorescence intensity was observed to reach a maximum for 90 vol % of H_2_O in the sample (205-fold increase in fluorescence intensity relative to the sample in 100% THF). Interestingly, for **2**, fluorescence intensity started to increase for samples containing more than 60 vol % of H_2_O, where the most significant (4-fold) increase in fluorescence intensity was observed between samples containing 80 and 90 vol % of H_2_O (maximum emission intensity, 187-fold increase in fluorescence intensity relative to the sample in 100% THF). For both compounds, further increasing the sample water content to 95 vol % resulted in a slight decrease in fluorescence intensity. The increase in the fluorescence intensity was accompanied by an increase in the fluorescence quantum yield (Φ_F_; relative method using quinine sulfate as a standard^91,92^ ). A 15-fold increase was observed for compound **1**, from 0.0096 for solution in THF to 0.1360 for aggregates in 90 vol % of H_2_O in THF (for compound **2**: from 0.008 to 0.0663, respectively; 8-fold increase). In addition, for solutions of aggregated **1** and **2**, a significant red shift of emission maxima (λ_em_), in comparison to the solution in THF, was observed (from 376 to 470 nm and from 400 to 475 nm for compounds **1** and **2**, respectively). For solid-state measurements, emission maximum (λ_em_) for **1** was observed at 458 nm (470 nm for aggregated **1**) and for **2** at 454 nm (474 nm for aggregated **2**) (for spectra see: Supporting Information, Section S5.1).

### Spectrofluorimetric Receptor Studies with **1** and **2**

Synthesized amidetriazoles were expected to exhibit receptor properties toward anions due to the presence of an amide bond, which allows the formation of noncovalent hydrogen bonds as well as electrostatic interactions with negatively charged analyte molecules (Figure 4a). In addition, together with the 1,2,3-triazole moieties, they form 1,4-dinitrogen systems, which may also play a role in the interaction with the analyte. These possible binding sites of **1**-**2** could be supported with the density functional theory (DFT) computed (B3LYP^93^/6–31g­(d,p)^94^) electrostatic surface potential (ESP) plots (see Figure 4b for ESP plots; for DFT-optimized geometries of **1**-**2** and DFT computational details, see Supporting Information, Section S4).

**Figure 4:**
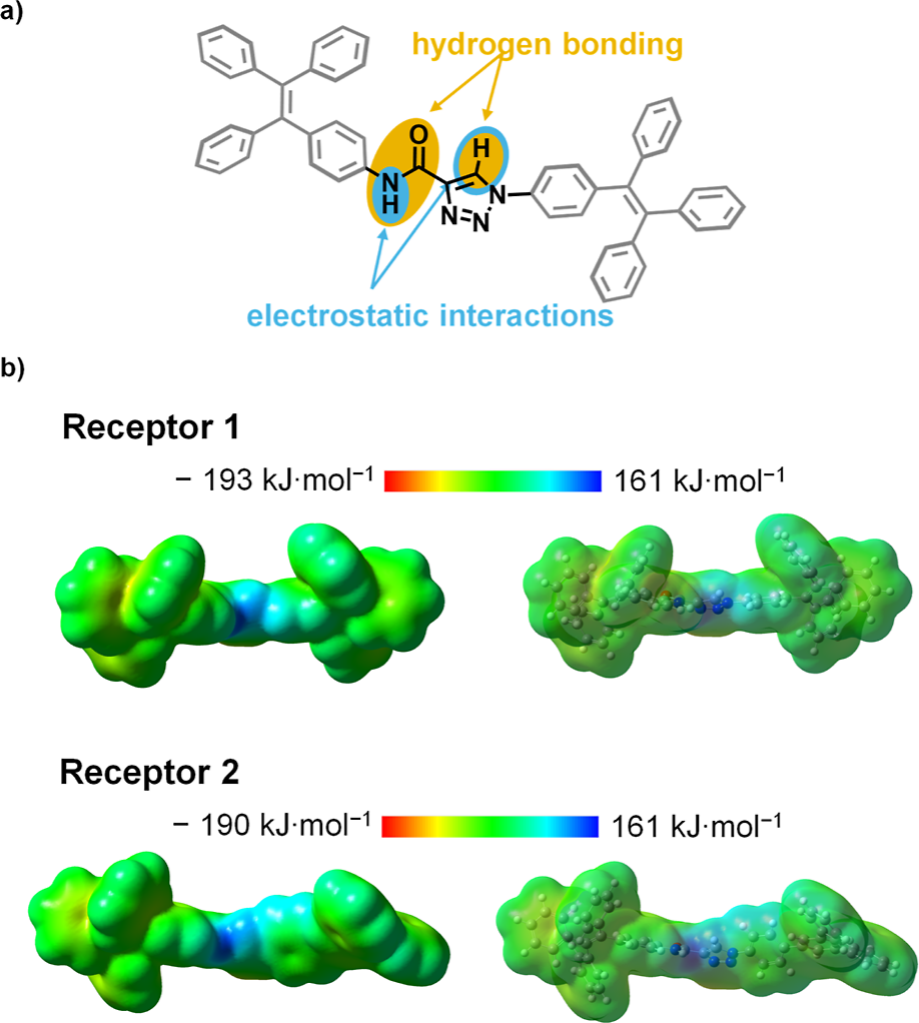
(a) Graphical presentation of the structural moieties of receptor **1** which participated in noncovalent interactions and the binding mechanism (orange = hydrogen bonding, blue = electrostatic interactions) and (b) DFT-computed (B3LYP/6–31g­(d,p)) ESP maps for receptors **1**-**2** (two views are presented).

First, to test a plausible binding mode, ^1^H NMR titrations were performed for the interaction of **1** with H_2_PO_4_^–^ as a representative anion (refer to Supporting Information, Section S7, for full data). Shifts for signals coming from both NH-amide and H-triazole were observed upon addition of further portions (molar equivalents) of H_2_PO_4_^–^ (in the form of tetrabutylammonium salt); in both cases, a downfield shift was observed (Figure 5). For anion contents above 0.5 equiv, NH-amide signal broadened significantly, and the signal coming from H-triazole was shifted significantly (from 9.3219 to 9.6620 ppm after addition of 5.0 equiv of H_2_PO_4_^–^). Shift of H_Ar_ signals, which were ascribed to the *p*-phenylene moieties in the structures of **1-2** (in the range 7.90–7.65 ppm), was also observed. This experiment revealed that the presence of the amidetriazole moiety played an essential role in the receptor properties of **1-2**. DFT computations (B3LYP/3–21g) were additionally performed for the representative **1-Br** ^**–**^ system to further support the binding mechanism concluded from the ^1^H NMR titration. These considerations included comparing the free enthalpy (Δ*G*) values for various complex arrangements, including those involving the analyte located in the proximity of (i) amidetriazole moiety (Figure 6, arrangement 1), (ii) triazole moiety, only (Figure 6, arrangement 2), and (iii) H–Ar from the *p*-phenylene moiety of TPE in conjunction with N–H from amide (Figure 6, arrangement 3). These studies supported the outcomes from the ^1^H NMR titration, since the complex involving analyte bound to the amidetriazole moiety of **1** (arrangement 1) featured a *ca*. 12–15 kJ^·^mol^–1^ lower Δ*G* value in comparison to the other tested system arrangements.

**Figure 5:**
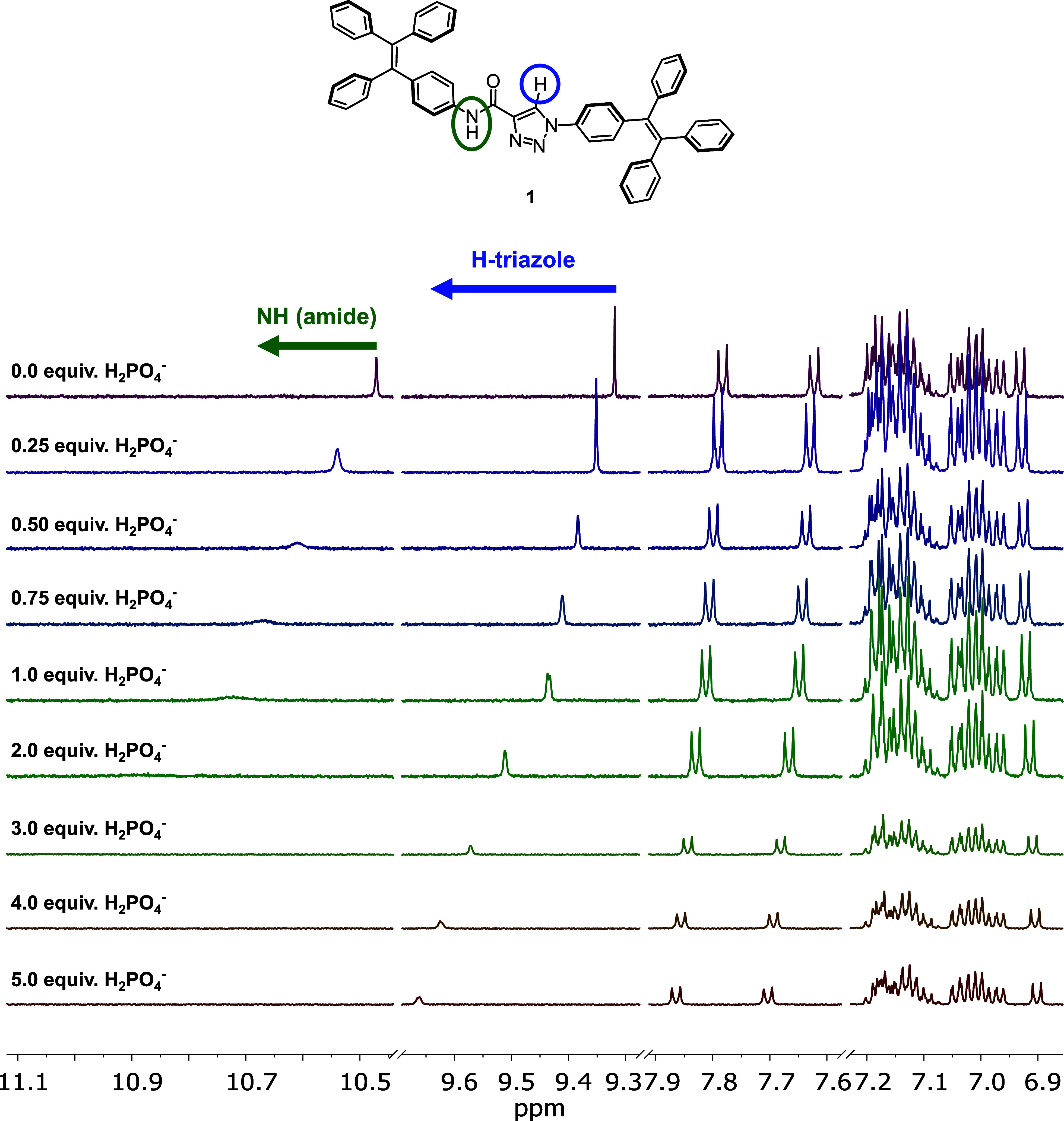
Representative key-insets regarding ^1^H NMR spectra titration experiment (600 MHz, DMSO-*d*_6_ + TMS, *C*_1_ = 1.29 mM) with compound **1** and H_2_PO_4_^–^.

**Figure 6:**
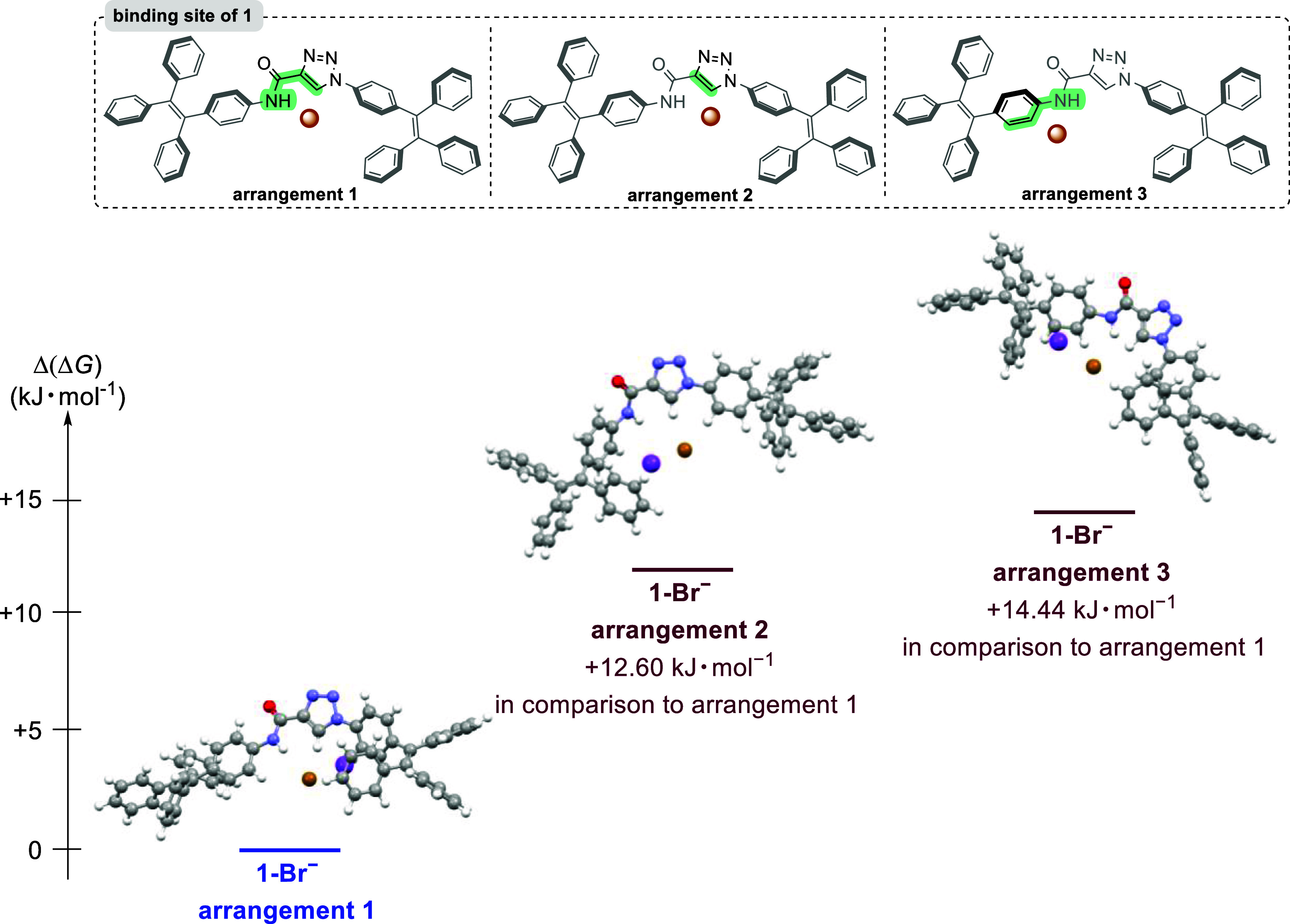
Graphical presentation of the results of DFT studies (B3LYP/3–21g) for system **1-Br** ^**–**^ (in the form of sodium salt) supporting the experimental (^1^H NMR) interaction pattern. A reference Δ*G* value was taken for the system featuring the lowest Δ*G* value (arrangement 1).

Receptor properties of **1** and **2** in solution were further tested in the aggregated state. Based on the results of initial AIE studies, a solvent system containing 95 vol % water and 5 vol % THF was chosen because it provided fluorescence intensity close to the maximal value while ensuring the highest possible water content in the sample. The ability to detect anions in samples with the highest possible water content is particularly important from a practical viewpoint. Spectrofluorimetric titrations were performed in a chosen solvent system using selected anions, namely, halide ions (F^–^, Cl^–^, Br^–^, I^–^) and trigonal planar (NO_3_^–^) and tetrahedral (H_2_PO_4_^–^ and SO_4_^2–^) oxyanions in the form of their tetrabutylammonium salts (refer to Supporting Information, Section S1.5 and S6, for full details). It should be noted that recognition occurs between compounds **1** and **2** in the form of aggregates, with the analytes (anions) dissolved in solution, indicating that this detection process is quasi-heterogeneous.

Spectrofluorimetric measurements showed that the emission intensity of the tested compounds decreases with the addition of subsequent portions of the analyte. The data collected from the spectrofluorimetric titrations were analyzed using the nonlinear data analysis tool, Bindfit.^95–97^ A representative example of emission spectra reflecting the interaction of **1** with the SO_4_^2–^ anion, together with a fitting plot, is presented in Figure 7a,b. Despite their relatively similar structures, compounds **1** and **2** exhibit noticeable differences in the obtained association constants, thereby affecting the selectivity and binding efficiency. Compound **1**, containing two TPE moieties in its structure, is characterized by higher values of the binding constant (*K*) at a satisfactory level of 10^5^ [M^–1^] for most of the analytes tested. Only for the sulfate anion (SO_4_^2–^) *K*_a_ value of 10^6^ [M^–1^] was obtained, suggesting selectivity of this receptor toward this anion in thermodynamic terms. DFT computations (B3LYP/3–21g) also supported this thermodynamic preference by comparing Δ*G* values for representative systems **1**-SO_4_^2–^ and **1**-Br^–^ (system arrangement 1, presented in Figure 6; sodium salts used in calculations, see data in Section S4, Supporting Information). It was found that the Δ*G* value for the **1**-SO_4_^2–^ system was *ca*. 70 kJ^·^mol^–1^ lower in comparison to the **1**-Br^–^ system, supporting the experimentally observed thermodynamic preference of receptor **1** toward binding SO_4_^2–^ over other analytes.

**Figure 7:**
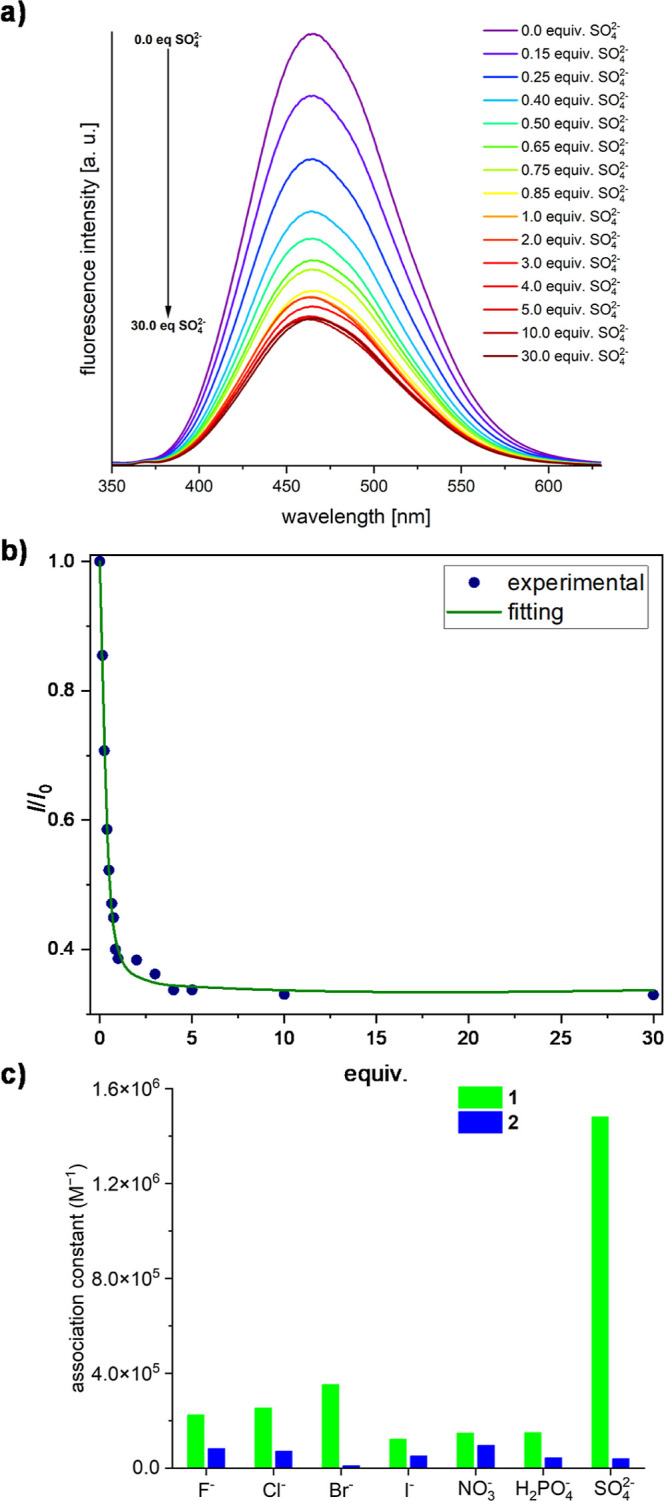
(a) Emission spectra of aggregated **1** as a representative receptor in the presence of increasing molar equivalents of SO_4_^2–^ as representative analyte; (b) titration curve and global fitting (Bindfit) regarding this titration, experimental conditions: H_2_O: THF = 95:5 *v/v*, C_1_ = 2·10^–5^ M, λ_ex_ = 326 nm, λ_em_ = 468 nm; and (c) graphical comparison of association constants (*K*_a_) values for the interaction between **1**-**2** and tested anions.

Compound **2**, containing both the TPE and TPB moieties in its structure, does not show similar selectivity, and the values of the association constants are at the lower level of 10^3^–10^4^ [M^–1^]. A graphical summary of the obtained association constants is shown in Figure 7c. The results clearly indicate that the introduction of two distinct AIE-active moieties into the receptor molecule negatively affects its detection capabilities. For both receptors and all analytes, a 2:1 stoichiometry was observed (i.e., two receptor molecules bind one analyte). The limit of detection (LOD) for the studied systems ranged from 0.38 to 5.32 μM (see Supporting Information, Section S6, for data). In particular, the LOD value for the interactions between receptor **1** and the SO_4_^2–^ anion was equal to 1.58 μM. Further, the receptor properties of aggregated **1** toward the detection of SO_4_^2–^ were tested using pH-dependent spectrofluorimetric titrations (buffer/THF = 95:5 *v/v*; refer to Supporting Information, Section S6, for spectra and data). Across all tested pH values (5.1, 7.4, and 8.2), receptor **1** showed SO_4_^2–^ sensing with a similar *K*_a_ of 10^5^ M^–1^. This confirms the wide applicability of chemoreceptor **1** in different aqueous media, including more complex matrixes.

To further investigate the mechanism of interaction between **1** and SO_4_^2–^ anion, Dynamic Light Scattering (DLS) analyses were performed (for full details see Supporting Information, Section S8). It was found that in the solvent system H_2_O: THF = 95:5 *v/v*, the average aggregate size was 1058 nm (315.2 nm for compound **2**). Addition of 5 equiv of SO_4_^2–^ caused aggregates to break down, resulting in an average size of 299.4 nm (a 3.5-fold decrease). It may be postulated that hydrogen bond formation and electrostatic interactions upon analyte binding to receptor **1** might promote deaggregation, resulting in a decrease in the mean hydrodynamic radius and in the fluorescence extinction.

### Electrochemical Receptor Studies with **1** and **2**

The selectivity of anion recognition by the studied receptors **1** and **2** was also investigated in a heterogeneous system: the derivatives were incorporated into polymeric membranes based on plasticized poly­(vinyl chloride) used in classical ion-selective electrodes. A lipophilic positively charged additive (tridodecylmethylammonium chloride—TDMAC) was added to the membranes to enhance their permselectivity for anions and ensure theoretical operation of the sensors.^98,99^ The anion selectivity of the membranes containing cationic sites (TDMA^+^) follows the so-called Hofmeister sequence.^98^ In this case, the preference toward lipophilic anions (e.g., ClO_4_^–^), characterized by less negative values of standard molar Gibbs free energy of hydration,^100^ is enhanced. For this reason, the selectivity of the membranes doped with the studied receptors should be compared to the selectivity determined for membranes containing only TDMAC (blank membranes).

The selectivity of anion binding in the polymeric phase was evaluated on the basis of the potentiometric selectivity coefficients log *K* (NO_3_^–^, X^–^), for membranes containing derivatives **1** and **2**, as shown in Figure 8 and listed in Table 2. The values of the selectivity coefficients express and quantify the influence of an arbitrarily chosen primary ion (in our case: NO_3_^–^) and a given interfering ion X^–^ on the signal of the ion-selective electrode. As expected, the results were strongly influenced by the presence of cationic sites in the membrane, with the highest values observed for lipophilic anions ClO_4_^–^ and I^–^, while the selectivity pattern was comparable for both derivatives. Nevertheless, a significant increase in membrane selectivity toward hydrophilic anions (especially SO_4_^2–^, F^–^, and H_2_PO_4_^–^) relative to blank membranes was noticed. For instance, a fifty-fold increase in the value of the selectivity coefficient toward sulfate anions was calculated for the membrane containing receptor **1**. This effect was much more pronounced for membranes doped with **1**, which could be related to their specific symmetrical structure. However, the differences in the anion association constants for the tested derivatives (calculated based on spectrofluorimetric experiments) were too low to reverse the potentiometric selectivity pattern of the membranes, consistent with the Hofmeister series. The observed enhanced selectivity toward SO_4_^2–^, analogous to results from spectrofluorimetric titrations, indicates the possibility of considering polyaromatic AIE-active chemoreceptors into the design of electrochemical sensors, thereby expanding their range of applications.

**Figure 8:**
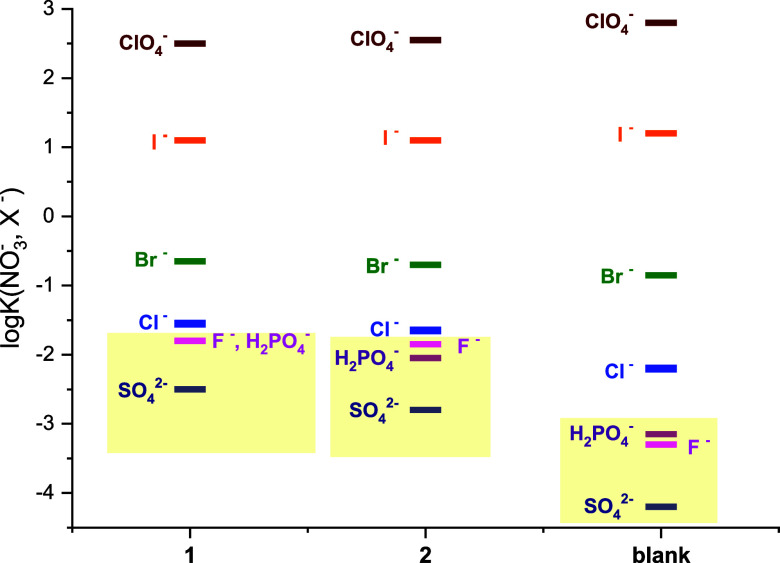
Graphical representation of the selectivity (values of log *K* (NO_3_^–^, X^–^)) of PVC/o-NPOE membranes formulated with receptors 1 and 2 (10 mol % TDMAC) and without a receptor (blank membrane); mean values were calculated for 3 electrode specimens.

**Table 2:** Values of Selectivity Coefficients log *K* (NO_3_^–^, X^–^) of PVC/o-NPOE Membranes Formulated with Receptors **1** and **2** (10 mol % TDMAC) and without a Receptor (Blank Membrane); Mean Values Were Calculated for 3 Electrode Specimens

	log *K* (NO_3_^–^, SO_4_^2–^)	log *K* (NO_3_^–^, F^–^)	log *K* (NO_3_^–^, H_2_PO_4_^–^)	log *K* (NO_3_^–^, Cl^–^)	log *K* (NO_3_^–^, Br^–^)	log *K* (NO_3_^–^, I^–^)	log *K* (NO_3_^–^, ClO_4_^–^)
**blank**	–4.20	–3.30	–3.15	–2.20	–0.85	1.20	2.80
**1**	–2,50	–1.80	–1.80	–1.55	–0.65	1.10	2.50
**2**	–2.80	–1.85	–2.05	–1.65	–0.70	1.10	2.55

### Comparison of Receptor Performance

Finally, we compared the performance of chemoreceptor **1** in recognizing SO_4_^2–^ with the parameters of the reported sensors. There are numerous reports in the literature on receptors developed for the recognition of sulfate ions, mainly fluorescent probes^101,102^ (Table 3). However, few of these studies calculate and report LOD values; titrations are usually performed in solution (NMR, UV–vis, or spectrofluorimetric); on this basis, association constants are determined and, in turn, a selectivity series is presented based on their values. We compared our results with several similar studies, for which selectivity and LOD values have been determined. Notably, among the reported systems, chemoreceptor **1** has dual receptor characteristics, enabling the detection of SO_4_^2–^ both spectrofluorimetrically and potentiometrically, with satisfactory parameters (*K*_a_ and LOD). Furthermore, thanks to the AIE effect (which is still rarely used for the detection of sulfate ions; Table 3, entries 2 and 5), receptor **1** offers a unique opportunity for the spectrofluorimetric detection of sulfate ions in solutions largely composed of water.

**Table 3:** Comparison of the Performance of Chemoreceptor **1** toward Detecting SO_4_^2–^ with Different Classes of the Reported Sensors^103–115^

entry	receptor	fluorescent receptor in solution	ionophore in membrane of potentiometric sensor	selectivity	limit of detection [μM]	binding constant 10^6^ [M^–1^]	ref.
1	amidotriazole 1	yes, AIE-active	yes	high selectivity for SO_4_^2–^ over inorganic anions (F^–^, Cl^–^, Br^–^, I^–^, H_2_PO_4_^–^, NO_3_^–^)	1.53	1.48	this work
2	naphthalimide derivative	yes, AIE-active	-	high selectivity for HSO_4_^–^/SO_4_^2–^ over inorganic anions (F^**–**^, Cl^**–**^, Br^**–**^, I^**–**^, ClO_4_^**–**^, NO_3_^**–**^, AcO^**–**^, H_2_PO_4_^**–**^, PO_4_^3**–**^, OH^**–**^)	3.25	see footnote^a^	^103^
3	nicotinamide–anthracene conjugate	yes	-	high selectivity for SO_4_^2–^ over inorganic anions (F^–^, Cl^–^, Br^–^, I^–^, AcO^–^, HSO_4_^–^, H_2_PO_4_^–^, CN^–^, OH^–^, NO_3_^–^, ClO_4_^–^, SCN^–^)	0.5	0.120	^104^
4	mono- and bisguanidine derivatives	yes	-	high selectivity for SO_4_^2–^ over inorganic anions (HPO_4_^2–^, PO_4_^3–^, Cl^–^, NO_3_^–^, CO_3_^2–^, AcO^–^, SCN^–^, SO_3_^2–^)	0.1	0.122	^105^
5	benzo[*e*]indol-3-ium derivative	yes, AIE-active (after interaction with analyte)	-	high selectivity for SO_3_^2–^ and SO_4_^2–^ over inorganic anions (F^–^, Cl^–^, Br^–^, I^–^, NO_2_^–^, NO_3_^–^, AcO^–^, H_2_PO_4_^–^, HCO_3_^–^, PF_6_^–^, ClO_4_^–^, SCN^–^, S_2_O_3_^2–^, S_2_O_8_^2–^, ascorbate, SH^–^)	0.53	-	^106^
6	copper(II) complex of naphthoic acid-based *N*-bridged tripodal ligand	yes	-	high selectivity for SO_4_^2–^ over inorganic anions (AcO^–^, NO_3_^–^, PO_4_^3–^, ClO_4_^–^, F^–^, Br^–^, I^–^)	0.5	620	^107^
7	ruthenium(II) arene complex	yes	-	high selectivity for SO_4_^2–^ and CO_3_^2–^ over inorganic anions (Cl^–^, Br^–^, I^–^, CO_3_^2–^, SO_4_^2–^, N_3_^–^, NO_2_^–^, SCN^–^, BPh_4_^–^, S_2_O_8_^2–^)	0.48	0.0259	^108^
8	Schiff base derived from 2-hydroxy-5-nitrobenzaldehyde and triethylenetetramine	-	yes	high selectivity for SO_4_^2–^ over inorganic anions (NO_3_^–^, Cl^–^, H_2_PO_4_^–^, SCN^–^, AcO^–^, Br^–^, ClO_4_^–^, HCO_3_^–^)	0.1	-	^109^
9	bis(cyclopeptide) based on l-proline	yes	-	selectivity decreases in the order: SO_4_^2–^ > I^–^ > Br^–^ > Cl^–^, NO_3_^–^	-	0.209	^110^
10	squaramide-based tripodal receptor	-^b^	-^b^	selectivity decreases in the order: SO_4_^2–^ > H_2_PO_4_^–^, HSO_4_^–^ ≫ AcO^–^, Cl^–^	-	0.00881	^111^
11	anthracene-pyridinium conjugate	yes, fluorescent probe in optical sensor	-	high selectivity for SO_4_^2–^ over inorganic anions (HPO_4_^2–^, Br^–^, Cl^–^, CO_3_^2–^, I^–^, SO_3_^2–^, NO^3–^)	-	-	^112^
12	TREN^c^-based tris(urea) receptor	-^b^	-^b^	selectivity decreases in the order: SO_4_^2–^, H_2_PO_4_^–^ > AcO^–^ > NO_3_^–^	-	0.0933	^113^
13	TREN^c^-based tris(ferrocenylurea) receptor	-^d^	-	selectivity decreases in the order: SO_4_^2–^ > F^–^ > AcO^–^ > H_2_PO_4_^–^ > Cl^–^ > HSO_4_^–^ > Br^–^ > NO_3_^–^ > I^–^	-	5.01	^114^
14	tetralactam-based macrocyclic receptor	-^b^	-^b^	high selectivity for SO_4_^2–^ over inorganic anions (NO_3_^–^, Cl^–^, Br^–^, I^–^, ClO_4_^–^, AcO^–^)	-	0.00289	^115^

a Binding constant value provided by the Authors of this paper was 54.34 μM^–1^.

b ^1^H NMR studies.

c TREN = *tris*(2-aminoethyl)­amine.

d UV–vis and ^1^H NMR studies.

## Conclusions

In conclusion, in this work, we present a new class of polyaromatic AIE-active amidetriazoles. Sono- and mechanochemical syntheses have enabled high yields to be achieved in a short time, outperforming the classical solvent-based synthesis method, while also improving the synthesis sustainability. Mechanochemical ball-milling not only enabled the efficient synthesis of AIE-active amidetriazoles but also opened new avenues for this class of derivatives. A nonclassical approach to copper-catalyzed 1,3-dipolar cycloaddition reactions, utilizing mechanochemistry and metallic copper rather than copper­(I), enabled high yields and introduced a new dimension to the chemistry of AIE-active molecular receptors. The obtained compounds were subjected to thorough spectroscopic and material analyses, revealing the influence of their structures on spectroscopic and receptor properties. Finally, recognition properties of synthesized receptors toward inorganic anions were tested. First, we have demonstrated by spectrofluorimetric titrations that receptor **1** (comprising two 1,1,2,2-tetraphenylethene skeletons in the structure) interacts selectively with SO_4_^2–^, with an association constant at the level of 10^6^ [M^–1^]. Compound **2** (comprising one 1,1,2,2-tetraphenylethene and one 1,3,5-triphenylbenzene skeleton in the structure) was characterized by *K*_a_ values at the level of 10^3^–10^4^ [M^–1^] and demonstrated an affinity similar to that of all tested anions, which elucidated structure–property behavior for the tested molecules. Next, for the first time in the field of AIE-active receptors, potentiometric ion-selective electrodes were constructed using membranes doped with compounds **1** and **2**. As in the case of spectrofluorimetric measurements, electrodes containing compound **1** exhibited more favorable recognition parameters, with a 50-fold increase in selectivity toward SO_4_^2–^ (compared to a reference electrode without a receptor in the membrane). The innovative use of AIE-active polyaromatic receptors in the design of electrochemical sensors could be considered an interesting subject of research; thus, we believe that our work opens up new avenues in the chemistry of AIE-active compounds.

## Experimental Section

### Materials

Chemical reagents and solvents were of the highest possible purity, purchased commercially, and purified according to standard methods. If necessary, thin-layer chromatography (TLC) and preparative thin-layer chromatography (PTLC; 2 mm) on SiO_2_ were performed using Merck Silica gel 60 F254 plates. Thin-layer chromatography (TLC) and column chromatography on Al_2_O_3_ were performed using an aluminum oxide 90 neutral gel (CarlRoth). Mechanochemical syntheses were performed in a ball mill Retsch MM400 with 1.5 or 5.0 mL stainless-steel jar with φ 3.0 mm stainless-steel balls (number of added balls is indicated in the synthesis section), frequency 30 Hz. For sonochemical reactions, Bandelin Sonorex RK 100H (ultrasonic probe; ultrasonic peak output/HF power: 320W/80W; 35 kHz) was used. The NMR experiments were carried out using a JEOL 600 MHz spectrometer (^1^H at 600 MHz, ^13^C­{^1^H} NMR at 151 MHz) equipped with a multinuclear z-gradient inverse probe head or Varian VNMRS 500 MHz spectrometer (^1^H at 500 MHz, ^13^C­{^1^H} NMR at 126 MHz) equipped with a multinuclear z-gradient inverse probe head. The spectra were recorded at 297.15 K and standard 5 mm NMR tubes were used. ^1^H NMR (δ_H_) and {^1^H}^13^C NMR (δ_C_) chemical shifts were reported in parts per million (ppm) relative to the solvent signal, i.e., DMSO-*d*_6_, δ_H_ (residual DMSO) 2.50 ppm, δ_C_ (residual DMSO) 39.50 ppm, CDCl_3_, δ_H_ (residual CHCl_3_) 7.26 ppm, δ_C_ (residual CHCl_3_) 77.16 ppm, THF-*d*_8,_ δ_H_ (residual THF) 1.73 and 3.58 ppm, and δ_C_ (residual THF) 25.4 and 67.6 ppm. ^1^H DOSY (Diffusion Ordered Spectroscopy) NMR experiments were performed at 297.15 K and using a Varian VNMRS 500 MHz spectrometer using a stimulated echo sequence incorporating bipolar gradient pulses^116^ and with convection compensation.^117^ ^1^H DOSY NMR spectra were analyzed with the DOSYToolbox^118^ software. The hydrodynamic radii of the selected molecules from ^1^H DOSY NMR experiments were calculated using the unmodified Stokes–Einstein equation^119^

$$r_{H,solv}=\frac{k_{B}T}{6\pi \eta D}$$

where *D* is the measured diffusion coefficient of the molecule, *k*_B_ is the Boltzmann constant (1.3806485 · 10^–23^ kg·s^–2^K^–1^), T is the temperature for the ^1^H DOSY NMR spectrum acquisition (298.15 K), *r*_H,solv_ is the calculated hydrodynamic radius, and η is the viscosity of the solvent (DMSO, 0.001991 kg·m^–1^s^–1^) at temperature T. **ESI-HRMS (TOF)** measurements were performed with a Q-Exactive ThermoScientific spectrometer. UV–vis spectra were recorded with a WVR UV-1600PC spectrometer, with a spectral resolution of 2 cm^–1^. For the UV–vis measurements, the wavelengths for the absorption maxima λ_max_ were reported in nm. Fluorescence spectra were recorded with a HITACHI F-7100 FL spectrometer. Parameters for the liquid spectra acquisition: scan speed: 1200 nm/min, delay: 0.0 s, EX slit: 5.0 nm, EM slit: 5.0 nm, PMT Voltage: 400 V. The wavelengths for the emission maxima (λ_em_) were reported in nm. Dynamic light scattering (DLS) measurements were performed with a Brookhaven Instruments Particle Size Analyzer 90Plus. EMF measurements were carried out using a potentiometric multiplexer (EMF 16 Interface, Lawson Laboratories Inc., Malvern, USA). The values of the potentiometric selectivity coefficients of the ion-selective electrodes log *K* (NO_3_^–^, X^–^) were determined by the separate solution method (SSM) using 0.01 M solutions of sodium salts containing 0.01 M MES pH 5.0.^98^ The activities of the ions in aqueous solutions were calculated according to the Debye–Hueckel approximation.

#### Synthesis of **1**

##### Synthesis of **1** in Solution

A mixture of **4** (24.0 mg; 0*.*075 mmol*;* 1.5 equiv), **5** (20.0 mg; 0.05 mmol; 1.0 equiv), CuTC (1.0 mg; 0.005 mmol; 0.1 equiv), and DIPEA (5.83 mg; 0.05 mmol; 1.0equiv) in *N*,*N*-dimethylformamide (DMF; 2 mL) was stirred for 48 h at room temperature. Distilled water (50 mL) was added, and the formed precipitate was filtered onto a nylon membrane (0.45 μm). The resulting solid was dissolved in CHCl_3_ (45 mL). After drying with MgSO_4_ followed by filtration, volatiles were distilled off on a rotary evaporator. Finally, the product was purified using column chromatography (SiO_2_, *c*-hexane/CHCl_3_ = 5/95 *v/v*, *R*_f_ = 0.44) to obtain a pale brown solid (20.10 mg; 52%). ^1^H NMR (THF-*d*_8_, 600 MHz, ppm) δH: 9.66 (s, 1H), 8.99 (s, 1H), 7.71 (m, 2H), 7.63 (m, 2H), 7.22 (m, 2H), 7.03–7.11 (m, 30H), 6,69 (m, 2H); {^1^H}^13^C NMR (THF-*d*_8_, 151 MHz, ppm) δC: 158.5, 145.7, 145.2, 145.1, 144.9, 144.9, 144.4, 144.4, 144.2, 143.3, 141.7, 141.6, 140.5, 140.1, 138.4, 136.0, 133.5, 132.2, 132.2, 132.1, 128.7, 128.7, 128.5, 128.4, 128.4, 127.6, 127.2, 127.2, 125.0, 120.3, 119.8; HRMS (ESI-TOF) *m*/*z*: [M^+^H]^+^ Calcd for C_55_H_40_N_4_O_1_, 722.31966; Found, 772.31950. For optimization studies see: Supporting Information section S1.2.1.

##### Mechanochemical Synthesis of **1**

Into a stainless-steel jar (volume 1.5 mL), compounds **4** (14.0 mg; 0.0375 mmol; 1.5equiv) and **5** (10.0 mg; 0.025 mmol; 1.0 equiv), together with a copper metal mesh (80.0 mg) and sodium l-ascorbate (5.0 mg; 0.025 mmol; 1.0 equiv), were added. Five stainless-steel grinding balls were added (diameter: 3.0 mm) together with dichloromethane (LAG; 25 μL). The reaction mixture was ground at a frequency of 30 Hz for 8 h. Dichloromethane (10–15 mL) was added to remove the solid reaction mixture from a jar. 1 M HCl (10 mL) was added, and the crude product was extracted with CH_2_Cl_2_ (3 × 20 mL). Organic layers were combined, washed with water and brine. After drying with MgSO_4_ followed by filtration, volatiles were distilled off on a rotary evaporator. Finally, the product was purified using column chromatography (SiO_2_, *c*-hexane/CHCl_3_ = 5/95 *v/v*, *R*_f_ = 0.44) to obtain a pale brown solid (32.85 mg; 85%). For optimization studies see: Supporting Information, section S1.2.1.

##### Notes on Mechanochemical Reactions with a Copper Metal Mesh

A copper metal mesh (>99.95% Cu) produced by PPH Polskie Odczynniki Chemiczne (now Avantor Performance Materials Poland S.A.). A copper metal mesh was cleaned before use by rinsing with 1 M HCl and then with distilled water, acetone, and finally dried in a stream of hot air. The mesh was then cut into 0.5 × 0.5 cm square sections; three such fragments were used for the synthesis with a total mass of 80.0 mg (eight times the weight of amide **5**).

##### Sonochemical Synthesis of **1**

Into a glass vial (ϕ_ext._: 16 mm, high: 150 mm), compounds **4** (14.0 mg; 0.0375 mmol; 1.5 equiv) and **5** (10.0 mg; 0.025 mmol; 1.0 equiv), together with a copper metal mesh (80.0 mg) and sodium l-ascorbate (5.0 mg; 0.025 mmol; 1.0 equiv) were placed together with dichloromethane (DCM; 50 μL). The vial was then placed in an ultrasonic bath for 6 h. Dichloromethane (20 mL) was added to remove the solid reaction mixture from the vial. 1 M HCl (20 mL) was added, and the crude product was extracted with CH_2_Cl_2_ (3 × 20 mL). Organic layers were combined and washed with water and brine. After drying with MgSO_4_ followed by filtration, volatiles were distilled off on a rotary evaporator. Finally, the product was purified using column chromatography (SiO_2_, *c*-hexane/CHCl_3_ = 5/95 *v/v*, *R*_f_ = 0.44) to obtain a pale brown solid (23.19 mg; 60%). For optimization studies see: Supporting Information, section S1.2.1.

#### Synthesis of **2**

##### Synthesis in Solution

A mixture of **7** (12.6 mg; 0.075 mmol; 1.5 equiv), **5** (18.0 mg; 0.05 mmol; 1.0 equiv), CuSO_4_·5H_2_O (11.4 mg; 0.05 mmol; 1.0 equiv), and sodium l-ascorbate (13.1 mg; 0.07 mmol; 1.4 equiv) in *N*,*N*-dimethylformamide (DMF; 2 mL) was stirred for 48 h at room temperature. Distilled water (50 mL) was added, and the formed precipitate was filtered on a nylon membrane (0.45 μm). The resultant solid was dissolved in CHCl_3_ (45 mL). After drying with MgSO_4_ followed by filtration, volatiles were distilled off on a rotary evaporator. Finally, the product was purified using column chromatography (SiO_2_, *n*-hexane/CHCl_3_ = 7/93 *v/v*, *R*_f_ = 0.67) to obtain a pale brown solid (20.92 mg; 56%). ^1^H NMR (THF-*d*_*8*_, 600 MHz, ppm) δH: 9.72 (s,1H), 9.16 (s, 1H), 8.12–8.10 (d, 2H), 8.05–8.03 (d, 2H) 7.95 (s, 2H), 7.91 (s, 2H), 7.79–7.78 (d, 4H), 7,67–7.66 (d, 2H) 7.48–7.46 (t, 4H), 7.38–7.37 (m, 2H), 7.11–6.98 (m, 18H); {^1^H}^13^C NMR (THF-*d*_*8*_, 150 MHz, ppm) δC: 158.6, 145.5, 145.1, 145.1, 145.1, 143.7, 142.8, 142.1, 141.8, 141.8, 140.2, 138.5, 137.4, 132.7, 132.4, 132.3, 129.8, 129.5, 128.7, 128.5, 128.2, 127.3, 127.3, 127.2, 126.5, 125.7125.4, 121.5, 120.1; ESI-HRMS (TOF): calcd for C_53_H_38_N_4_O_1_ [M^+^H]^+^ *m*/*z* = 746.3040, found, 746.3042.

##### Mechanochemical Synthesis of **2**

Into a stainless-steel jar (volume 1.5 mL) compounds **7** (13.0 mg; 0.0375 mmol; 1.5equiv) and **5** (10.0 mg; 0.025 mmol; 1.0 equiv) together with a copper metal mesh (80.0 mg) and sodium l-ascorbate (5.0 mg; 0.025 mmol; 1.0 equiv) were added. Five stainless-steel grinding balls were added (diameter: 3.0 mm) together with dichloromethane (LAG; 25 μL). The reaction mixture was ground with a frequency of 30 Hz, for 6 h. Dichloromethane (10–15 mL) was added to remove the solid reaction mixture from a jar. 1 M HCl (10 mL) was added, and the crude product was extracted with CH_2_Cl_2_ (3 × 20 mL). Organic layers were combined, washed with water and brine. After drying with MgSO_4_ followed by filtration, volatiles were distilled off on a rotary evaporator. Finally, the product was purified using column chromatography (SiO_2_, *n*-hexane/CHCl_3_ = 7/93 *v/v*, *R*_f_ = 0.67) to obtain a pale brown solid (18.68 mg; 50%). For optimization studies see: Supporting Information, Section S1.2.2.

##### Sonochemical Synthesis of **2**

Into a glass vial (ϕ_ext._: 16 mm, high: 150 mm), compounds **7** (13.0 mg; 0.0375 mmol; 1.5 equiv) and **5** (10.0 mg; 0.025 mmol; 1.0 equiv), together with a copper metal mesh (80.0 mg) and sodium l-ascorbate (5.0 mg; 0.025 mmol; 1.0 equiv), were placed together with dichloromethane (DCM; 50 μL). The vial was then placed in an ultrasonic bath for 6 h. Dichloromethane (20 mL) was added to remove the reaction mixture from the vial. 1 M HCl (20 mL) was added, and the crude product was extracted with CH_2_Cl_2_ (3 × 20 mL). Organic layers were combined and washed with water and brine. After drying with MgSO_4_ followed by filtration, volatiles were distilled off on a rotary evaporator. Finally, the product was purified using column chromatography (SiO_2_, *n*-hexane/CHCl_3_ = 7/93 *v/v*, *R*_f_ = 0.67) to obtain a pale brown solid (22.41 mg; 60%).

#### Synthesis of **4**

To the flask containing solution concentrated hydrochloric acid (HCl; 1100.0 μL) in water (16.0 mL), 4-(1,2,2-triphenylvinyl)­aniline (**3**; 100.0 mg; 0.146 mmol; 1.0 equiv) was added, followed by sodium nitrite (NaNO_2_; 181.4 mg; 2.260 mmol; 9.0 equiv). The reaction mixture was stirred vigorously for 2 h in an ice bath. Sodium azide (NaN_3_; 76.0 mg; 1.152 mmol; 4.0 equiv) was added, and the reaction mixture was stirred for 2 h at room temperature. The crude product was extracted with CHCl_3_ (3 × 20 mL). Organic layers were combined and washed with brine and water. After drying with MgSO_4_ followed by filtration, volatiles were distilled off on a rotary evaporator. Finally, the product was purified using column chromatography (SiO_2_, *n*-hexane/CHCl_3_ = 1/1 *v/v*, *R*_f_ = 0.8) to obtain a yellow solid (52.34 mg; 96%). ^1^H NMR (CDCl_3_, 600 MHz, ppm) δH: 7.10–7,13 (m, 9H), 7.00–7.09 (m, 8H), 6.77 (m, 2H); {^1^H}^13^C NMR (CDCl_3_, 151 MHz, ppm) δC: 143.7, 143.6, 143.6, 141.4, 140.7, 140.0, 138.1, 132.9, 131.4, 131.4, 131.4, 127.9, 127.8, 127.8, 126.7, 126.6, 118.5; HRMS (ESI-TOF) *m*/*z*: [M^+^H]^+^ Calcd for C_26_H_19_N_3_, 373.1575; Found, 373.15723. For optimization studies see: Supporting Information, Section S1.2.3.

#### Synthesis of **5**

##### Synthesis of **5** in Solution

A mixture of propiolic acid (20.2 mg; 0.288 mmol; 1.0 equiv), and *N,N′*-dicyclohexylcarbodiimide (DCC; 59.4 mg; 0.288 mmol; 1.0 equiv) in dichloromethane (DCM; 2.0 mL) were stirred for 20 min under an argon atmosphere in an ice bath. Then, a solution of 4-(1,2,2-triphenylvinyl)­aniline (**3**; 100.0 mg; 0.288 mmol; 1.0 equiv) and *N*-hydroxysuccinimide (NHS; 33.1 mg; 0.288 mmol; 1.0 equiv) in dichloromethane (DCM; 2.0 mL) was added and the reaction mixture was stirred at room temperature for 72 h. 1 M HCl (10 mL) was added, and the crude product was extracted with dichloromethane (CH_2_Cl_2_; 3 × 20 mL). Organic layers were combined and washed with water and brine. After drying with MgSO_4_ followed by filtration, volatiles were distilled off on a rotary evaporator. Finally, the product was purified using column chromatography (Al_2_O_3_, *n*-hexane/dichloromethane = 2/9 *v/v*) (*R*_f_ = 0.45) to obtain **5** as pale yellow solid (92.0 mg; 80%). ^1^H NMR (DMSO-*d*_6_, 600 MHz, ppm) δH: 10.73 (s, 1H), 7.35–7.33 (m, 2H), 7.15–7.10 (m, 9H), 6.98–6.94 (m, 7H), 6.90–6.89 (m, 2H), 4.37 (s, 1H); {^1^H}^13^C NMR (DMSO-*d*_6_, 150 MHz, ppm) δC: 149.5, 143.2, 143.2, 143.1, 140.4, 140.0, 139.1, 136.5, 131.1, 130.6, 130.6, 130.6, 127.9, 127.8, 127.8, 126.6, 126.5, 119.0, 78.3, 77.1; ESI-HRMS (TOF): calcd for C_29_H_21_N_1_O_1_: [M^+^H]^+^ *m*/*z* = 400.1698, found, 400.1696. For optimization studies see: Supporting Information Section S1.2.4.

##### Mechanochemical Synthesis of **5**

Into a stainless-steel jar (volume 1.5 mL), compounds **3** (50.0 mg; 0.146 mmol; 1.0 equiv) and propiolic acid (10.9 mg; 0.146 mmol; 1.0 equiv) together with *N,N′*-dicyclohexylcarbodiimide (DCC; 32.1 mg; 0.146 mmol; 1.0 equiv) and *N*-hydroxysuccinimide (NHS; 17.9 mg; 0.146 mmol; 1.0 equiv). Five stainless-steel grinding balls were added (diameter: 3.0 mm) together with LAG solvent (DCM; 15 μL). A reaction mixture was ground with a frequency of 30 Hz for 3 h. Dichloromethane (10–15 mL) was added to remove the solid reaction mixture from a jar. 1 M HCl (10 mL) was added, and the crude product was extracted with dichloromethane (CH_2_Cl_2_; 3 × 20 mL). Organic layers were combined and washed with water and brine. After drying with MgSO_4_ followed by filtration, volatiles were distilled off on a rotary evaporator. Finally, the product was purified using column chromatography (Al_2_O_3_, *n*-hexane/dichloromethane = 2/9 *v/v*) (*R*_f_ = 0.45) to obtain **5** as a pale yellow solid (33.13 mg; 36%). For optimization studies see: Supporting Information, Section S1.2.4.

#### Synthesis of **7**

To the flask containing solution-concentrated hydrochloric acid (HCl; 280 μL) in water (16.0 mL), 5′-phenyl-[1,1’:3′,1″-terphenyl]-4-amine (**6**; 100.0 mg; 0.31 mmol; 1.0 equiv) was added, followed by sodium nitrite (NaNO_2_; 21.4 mg; 4.5 mmol; 9.0 equiv). The reaction mixture was stirred vigorously for 2 h. Sodium azide (NaN_3_; 81.0 mg; 1.2 mmol; 4.0 equiv) was added and the reaction mixture was stirred for 2 h. The crude product was extracted with CHCl_3_ (3 × 20 mL). Organic layers were combined and washed with brine and water. After drying with MgSO_4_ followed by filtration, volatiles were distilled off on a rotary evaporator. Finally, the product was purified using column chromatography (SiO_2_, *n*-hexane/CHCl_3_ = 4/6 *v/v*, *R*_f_ = 0.8) to obtain a yellow solid (100.20 mg; 93%). ^1^H NMR (600 MHz, CDCl_3_, ppm) δH: 7.78 (d, 1H), 7.75 (d, 2H), 7.73–7.67 (m, 6H), 7.51–7.46 (m, 4H), 7.41 (m, 2H), 7.17–7.11 (m, 2H) {^1^H}^13^C NMR (CDCl_3_, 151 MHz, ppm) δC: 142.5, 141.2, 141.0, 139.4, 137.9, 129.5, 128.9, 128.6, 128.1, 127.6, 127.3, 125.3, 124.8, 119.5; HRMS (ESI-TOF) *m*/*z*: [M^+^H]^+^ Calcd for C_24_H_17_N_3_ 348.1495; Found, 348.1495. For optimization studies see: Supporting Information, Section S1.2.5.

### Aggregation-Induced Emission Studies—Preparation of the Samples

The studies on the aggregation-induced emission (AIE) behavior were performed by employing measurements of the fluorescence spectra. The experiments were performed in the H_2_O/THF solvent mixtures. Stock solutions of **1** and **2** (2·10^–3^ M) in THF were diluted with proper volume of pure THF followed by addition of H_2_O to reach given vol % of H_2_O in the sample.

### Estimation of Fluorescence Quantum Yield

The measurements for the estimation of fluorescence quantum yields (Φ_F_) for **1** and **2**, as well as their aggregates were performed at room temperature according to the literature procedures.^91,92^ Fluorescence quantum yields (Φ_F_) were determined by comparison with quinine sulfate (QS) in 0.5 M H_2_SO_4_ (Φ_F,ref_ = 0.5)^91^ as the standard. The measurements were performed with highly diluted solutions, for which absorbance (A) values for the highest wavelength were not higher than 0.1 au. The excitation wavelengths (λ_ex_) for each sample were selected on the basis of the UV–vis spectra and were as follows: λ_ex_ = 320 nm for **1**, λ_ex_ = 320 nm for **2**, and concentrations were as follows: *C*_QS_ = 2·10^–6^ M; *C*_molecule/aggregates_ = 2·10^–6^ M. The following formula was used for the calculation of Φ_F_

$$\phi_{F}=\phi_{F,ref}\cdot \frac{F_{sample}}{F_{reference}}\cdot \frac{1-10^{-A_{ref}}}{1-10^{-A_{sample}}}\cdot \frac{n_{sample}^{2}}{n_{reference}^{2}}$$

where Φ_F,ref_ is the quantum yield for QS (0.551), F is the integrated area under the fluorescence spectra, A is the absorbance at the excitation wavelength, *n* is the refractive index of the solvent (1.346 for 0.5 M H_2_SO_4_, 1.4072 for THF, *n* for the aggregate solution in the given volume ratio was taken as the weighted arithmetic mean with weights equal to vol % of H_2_O and THF in the mixture).

### Receptor Studies—Titration Experiments Methodology

The anion binding experiments between **1** and **2** (receptors) and cations (analytes; F^–^, Cl^–^, Br^–^, I^–^, NO_3_^–^, H_2_PO_4_^–^, and SO_4_^2–^ in the form of tetrabutylammonium ([N­(C_4_H_9_)_4_]^+^) salts) were performed employing the fluorescence spectra titration experiments. The experiments were performed in the H_2_O/THF = 95:5 *v/v* system as follows. Stock solutions of **1** or **2** (2·10^–3^ M) in THF were diluted with adequate volume of pure THF and H_2_O to reach the final sample volume of 3 mL and the desired composition of solvents. The given anion was introduced into the mixture in the form of H_2_O/THF = 95:5 *v/v* solutions. Each titration experiment consisted of 15 steps. First, fluorescence of a solution containing only a receptor was measured, then solutions containing given cations were added in 14 consecutive steps to achieve the following proportions of cation to receptor: 0.00, 0.15, 0.25, 0.4, 0.5, 0.65, 0.75, 0.85, 1.0, 2.0, 3.0, 4.0, 5.0, 10.0, 30.0 equiv. To ensure proper mixing, before the measurement of the spectrum, the contents of the cuvette were well-mixed using a magnetic stirrer (1200 rpm). For the studies using buffer solutions, buffers of appropriate pH were used instead of distilled water, and the titration experiments were performed as described above. The following buffers were used: pH 5.1 MES buffer (*C* = 0.01 M), pH 7.4 PBS buffer (*C* = 0.01 M), and pH 8.2 *Tris* buffer (*C* = 0.01 M). The limit of detection (LOD) value for each system was calculated using a linear plot: (*I*-*I*_min_)/(*I*_max_-*I*_min_) = *f*(log­(*C*)). The *x* value for *y* = 1 was calculated (value *x*(*y* = 1)), and then LOD was taken as 10^*x*(*y*=1)^.

### Sensors Preparation and EMF Measurements

The membrane preparation method was the same as that for standard ion-selective electrodes. The membranes contained: 1 wt % receptor (compounds **1**-**2**), 65–66 wt % plasticizer (*o*-NPOE), 32–33 wt % PVC, and 10% mol (vs receptor) TDMAC (lipophilic additive). The membrane components (200 mg in total) were dissolved in 1.5 mL of THF. The solution was poured into a glass ring placed on a glass. After solvent evaporation, membrane discs of appropriate size were cut off and mounted in electrode bodies (type IS 561, Philips) for electromotive force (EMF) measurements. NaCl solution (0.01 M) was used as an internal filling; the electrodes were conditioned overnight in a NaCl solution (0.001 M). For each membrane composition, at least three sensor specimens were prepared. All measurements were carried out with cells of the following type: Ag, AgCl; KCl 1 M/CH_3_COOLi 1M/sample solution//membrane//internal filling solution; AgCl, Ag.

## Supplementary Material



## Data Availability

The data underlying this study are available in the published article and its Supporting Information.

## Notes

Materials and methods, experimental procedures, compounds characterization data, and data on: AIE studies, receptor studies and DFT computations details (PDF)
