# Supplementary material for: Mechanochemically Synthesized Amidetriazoles for Effective Optical and Electrochemical Detection of Anions
Source: J Org Chem. 2026 Mar 27;91(14):4945–61. doi: 10.1021/acs.joc.5c03014 (PMC13298899; doi:10.1021/acs.joc.5c03014)
Supplement: Supplementary file 1 [file jo5c03014_si_001.pdf]

SUPPORTING INFORMATION (SI) FOR

Mechanochemically Synthesised Amidetriazoles for Effective Optical and Electrochemical Detection of Anions

Jakub S. Cyniak[a,*], Daria Szeląg[a], Róża Sitek[a], Wojciech Wróblewski[a], Artur Kasprzak[a,*]

[a] Faculty of Chemistry, Warsaw University of Technology, Noakowskiego Str. 3, 00-664 Warsaw, Poland

* Corresponding authors e-mail: artur.kasprzak@pw.edu.pl (A.K.), jakub.cyniak.dokt@pw.edu.pl (J.S.C).

Table of contents

List of abbreviations S2

S1. Experimental section S3

S1.1 Materials and methods S3

S1.2 Synthesis S5

S1.2.1 Compound 1 .... S6

S1.2.2 Compound 2 .... S9

S1.2.3 Compound 4 .... S10

S1.2.4 Compound 5 .... S10

S1.2.5 Compound 7 .... S12

S1.3 Aggregation-induced emission (AIE) studies – preparation of the samples S13

S1.4 Estimation of fluorescence quantum yield S13

1.5 Receptor studies – titration experiments methodology S13

1.6 Sensors preparation and EMF measurements S14

S2. NMR spectra S15

S3. HRMS spectra S22

S4. DFT computations S25

S5. Photophysical and AIE studies S46

S5.1 Fluorescence spectra S46

S5.2 AIE-studies – spectrofluorimetry S47

S6. Receptor studies – spectrofluorimetry S49

S7. Receptor studies – NMR S73

S8. DLS S74

S9. supplementary references S76

List of abbreviations

- **DCC** - *N,N'*-dicyclohexylcarbodiimide
- **NHS** - *N*-hydroxysuccinimide
- **LAG** - Liquid Assisted Grinding
- **CuTC** - Copper(I) thiophene-2-carboxylate
- **DIPEA** - *N,N*-diisopropylethylamine
- **PVC** - poly(vinyl chloride)
- **o-NPOE** - 2-nitrophenyl octyl ether
- **TDMAC** - tridodecylmethylammonium chloride
- **EMF** - electromotive force
- **MES** - 2-morpholin-4-ylethanesulfonic acid

S1. Experimental section

S1.1 Materials and methods

Materials. Chemical reagents and solvents were of the higher possible purity and were commercially purchased and purified according to the standard methods, if necessary Thin layer chromatography (TLC) and preparative thin layer chromatography (PTLC; 2 mm) on SiO2 were performed using Merck Silica gel 60 F254 plates. Thin layer chromatography (TLC) and column chromatography on Al_2O_3 were performed using aluminium oxide 90 neutral gel (CarlRoth).

Mechanochemical syntheses were performed in a ball mill Retsch MM400 with 1.5 mL or 5.0 mL stainless steel jar with φ 3.0 mm stainless steel balls (number of added balls is indicated in the synthesis section), frequency 30 Hz.

For sonochemical reactions Bandelin Sonorex RK 100H (ultrasonic probe; ultrasonic peak output/HF power: 320W/80W; 35kHz) was used.

The NMR experiments were carried out using JEOL 600 MHz spectrometer (^{1}H at 600 MHz, ^{13}C{^{1}H} NMR at 151 MHz) equipped with a multinuclear z-gradient inverse probe head or Varian VNMRS 500 MHz spectrometer (^{1}H at 500 MHz, ^{13}C{^{1}H} NMR at 126 MHz) equipped with a multinuclear z-gradient inverse probe head. The spectra were recorded at 297.15 K and standard 5 mm NMR tubes were used. ^{1}H NMR (δ_H) and {^{1}H}^{13}C NMR (δ_C) chemical shifts were reported in parts per million (ppm) relative to the solvent signal, *i.e.* DMSO-d_6 δ_H (residual DMSO) 2.50 ppm, δ_C (residual DMSO) 39.50 ppm, $CDCl_3$, δ_H (residual $CHCl_3$) 7.26 ppm, δ_C (residual $CHCl_3$) 77.16 ppm, THF-d_8 δ_H (residual THF) 1.73 and 3.58 ppm, δ_C (residual THF) 25.4 and 67.6 ppm.

^{1}H DOSY (Diffusion Ordered Spectroscopy) NMR experiments were performed at 297.15 K and using a Varian VNMRS 500 MHz spectrometer using a stimulated echo sequence incorporating bipolar gradient pulses and with convection compensation. ^{1}H DOSY NMR spectra were analyzed with the DOSYToolbox software. The hydrodynamic radii of the selected molecules from ^{1}H DOSY NMR experiments were calculated using the unmodified Stokes-Einstein equation:

$$r_{H,solv} = \frac{k_B \mathrm{T}}{6\pi\eta D}$$

where D is the measured diffusion coefficient of the molecule, k_B is the Boltzmann constant ($1.3806485 \cdot 10^{-23}$ kg·s^{-2}K^{-1}), T is the temperature for the ^{1}H DOSY NMR spectrum acquisition (298.15 K), $r_{H,solv}$ is the calculated hydrodynamic radius, η is the viscosity of the solvent (DMSO, 0.001991 kg·m^{-1}s^{-1}) at temperature T.

ESI-HRMS (TOF) measurements were performed with a Q-Exactive ThermoScientific spectrometer.

UV-vis spectra were recorded with a WVR UV-1600PC spectrometer, with the spectral resolution of 2 cm^{-1}. For the UV-Vis measurements, the wavelengths for the absorption maxima λ_{max} were reported in nm.

Fluorescence spectra were recorded with a HITACHI F-7100 FL spectrometer. Parameters for the liquid spectra acquisition: scan speed: 1200 nm/min, delay: 0.0 s, EX slit: 5.0 nm, EM slit: 5.0 nm, PMT Voltage: 400 V. The wavelengths for the emission maxima (λ_{em}) were reported in nm.

Dynamic light scattering (DLS) measurements were performed with Brookhaven Instruments Particle Size Analyser 90Plus.

EMF measurements were carried out using potentiometric multiplexer (EMF 16 Interface, Lawson Labs Inc., Malvern, USA) . The values of the potentiometric selectivity coefficients of the ion-selective electrodes log K (NO_3^-, X^-) were determined by the separate solution method (SSM) using 0.01 M solutions of sodium salts containing 0.01M MES pH 5.0[1]. The activities of ions in aqueous solutions were calculated according to the Debye-Hueckel approximation.

S1.2 Synthesis

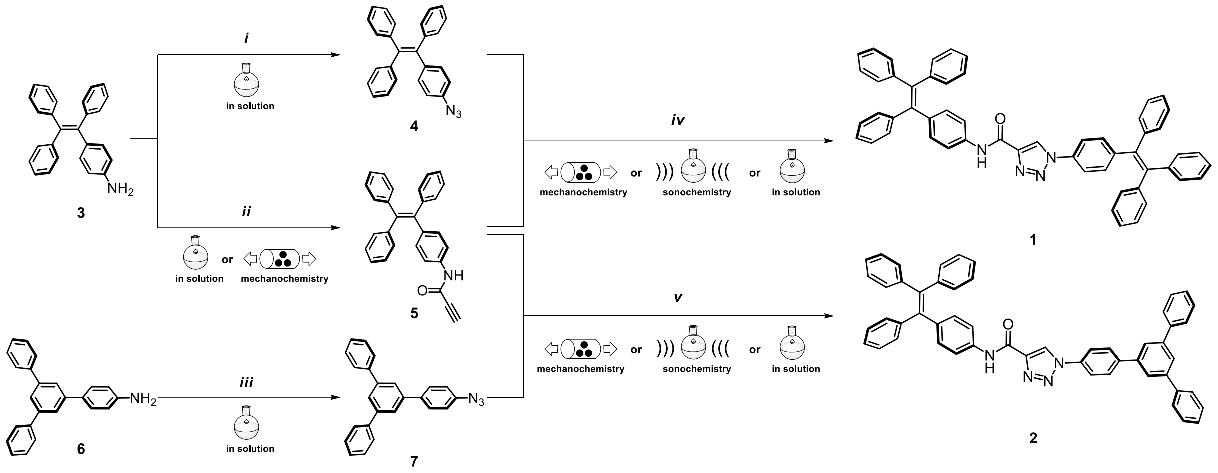

Scheme S 1 Synthesis of compounds **1** – **2** and their precursors **4**, **5**, **7**; conditions: **i)** *in solution:* $NaNO_2$, NaN_3, HCl_{aq}, 4h, 0°C→RT, 96%; **ii)** *in solution:* propiolic acid, DCC, NHS, DCM, 48h, RT, 80%; *mechanochemistry (ball mill):* propiolic acid, DCC, NHS, DCM (LAG), 30Hz, 3h, 36%; **iii)** *in solution:* $NaNO_2$, NaN_3, HCl_{aq}, 4h, RT, 93%; **iv)** *in solution:* CuTC, DIPEA, DMF, 48h, RT, 52%; *mechanochemistry (ball mill): copper* metal mesh (>99,95% Cu), sodium L-ascorbate, DCM (LAG), 8h, RT, 85%; *sonochemistry*: *copper* metal mesh (>99,95% Cu), sodium L-ascorbate, DCM, 6h, RT, *60%;* **v)** *in solution*: $CuSO_4{\cdot}H_2O$, sodium L-ascorbate, DMF, 48h, RT, 56%; *mechanochemistry (ball mill): copper* metal mesh (>99,95% Cu), sodium L-ascorbate, DCM (LAG), 6h, RT, *50%; sonochemistry*: *copper* metal mesh (>99,95% Cu), sodium L-ascorbate, DCM (LAG), 6h, RT, *40%*

HAZARD IDENTIFICATION

$NaNO_2$	NaN_3	DIPEA
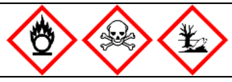	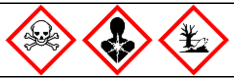	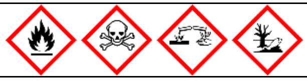
**H272** (May intensify fire; oxidizer)	**H300** (fatal if swallowed)	**H225** (highly flammable liquid and vapour)
**H301** (toxic if swallowed)	**H330** (fatal if inhaled)	**H302** (harmful if swallowed)
**H319** (causes serious eye irritation)	**H310** (fatal in contact with skin)	**H331** (toxic if inhaled)
**H400** (very toxic to aquatic life)	**H373** (may cause damage to organs through prolonged or repeated exposure if swallowed)	**H318** (causes serious eye damage)
	**H400** (very toxic to aquatic life)	**H335** (may cause respiratory irritation)
	**H410** (very toxic to aquatic life with long lasting effects)	**H411** (toxic to aquatic life with long lasting effects)
**for more information about hazards, first aid, firefighting measures, handling and storage see safety data sheet:**		
https://tiny.pl/t_pkm5r92	https://tiny.pl/p6dzwf019	https://tiny.pl/hxwtkh9tm

Caution! *Sodium nitrite is an oxidiser and may intensify fire. Keep away from heat, hot surfaces, sparks, open flames, and other ignition sources. Causes serious eye irritation. If in eyes: rinse cautiously with water for several minutes. Remove contact lenses, if present and easy to do.*

Caution! *Sodium azide is fatal if swallowed, in contact with skin, or if inhaled. May cause damage to organs (including the brain) through prolonged or repeated exposure if swallowed.*

Caution! *N,N-Diisopropylethylamine (DIPEA) is highly flammable as a liquid and a vapour. Keep away from heat, hot surfaces, sparks, open flames, and other ignition sources. Harmful and toxic if swallowed or inhaled. May cause respiratory irritation. Causes serious eye damage. Wear protective gloves/ protective clothing/ eye protection.*

S1.2.1 Compound 1

General procedure for the synthesis of **1** in solution:

Mixture of **4** (1.5 equiv.), **5** (1.0 equiv.) and copper source (with reductor and/or additional reagents when required; see: Table S 1, column D) in *N,N*-Dimethylformamide (DMF; 2 ml) was stirred for 48 hours at room temperature. Distilled water (50 mL) was added, and the formed precipitate was filtered on a nylon membrane (0.45 μm). The resulting solid was dissolved in $CHCl_3$ (45 ml). After drying with $MgSO_4$ followed by filtration, volatiles were distilled off on a rotary evaporator. Finally, the product was purified using column chromatography (SiO_2, c-hexane/$CHCl_3$ = 5/95 *v/v*, R_f =0.44) to obtain a pale brown solid (52%).

^{1}H NMR (THF–d_8, 600 MHz, ppm) δH: 9.66 (s, 1H), 8.99 (s, 1H), 7.71 (m, 2H), 7.63 (m, 2H), 7.22 (m, 2H), 7.03 – 7.11 (m, 30H), 6,69 (m, 2H);
{^{1}H}^{13}C NMR (THF–d_8, 151 MHz, ppm) δC: 158.5, 145.7, 145.2, 145.1, 144.9, 144.9, 144.4, 144.4, 144.2, 143.3, 141.7, 141.6, 140.5, 140.1, 138.4, 136.0, 133.5, 132.2, 132.2, 132.1, 128.7, 128.7, 128.5, 128.4, 128.4, 127.6, 127.2, 127.2, 125.0, 120.3, 119.8;
ESI-HRMS (TOF): calcd. for $C_{55}H_{40}N_4O_1$ $[M^+H]^+$, m/z= 722.31966, found: 772.31950

Table S 1 Conditions for synthesis of compound **1** in solution

A	B	C	D	E
**entry**	**4** (mg; *mmol*; eq)	**5** (mg; *mmol*; eq)	**other** (mg; *mol*; eq)	**yield** (%)
**1**	24.0; *0.075*; 1.5	20.0; *0.05*; 1.0	**copper metal mesh** 80.0; -; -<br>**sodium L-ascorbate** 5.0; *0.025*; 1.0	**0**
**2**			**$CuSO_4 \cdot H_2O$** 18.8; *0.065*; 1.5<br>**sodium L-ascorbate** 20.2; *0.01*; 2.0	**40**
**3**			**CuTc** 1.0; *0.005*; 0.1<br>**DIPEA** 5.83; *0.05*; 1.0	**52**

General procedure for mechanochemical synthesis of **1**:

Into a stainless-steel jar (volume 1.5 mL), compounds **4** (1.5 equiv.) and **5** (1.0 equiv.), together with a copper source (with reductor and/or additional reagents when required; see: **Table S 2**, column D), were added. Five stainless steel grinding balls were added (diameter: 3.0 mm) together with dichloromethane (LAG, when required; 25 μL). The reaction mixture was ground at a frequency of 30 Hz for 3-8 hours. Dichloromethane (10-15 mL) was added to remove the solid reaction mixture from a jar. 1M HCl (10 mL) was added, and the crude product was extracted with CH_2Cl_2 (3x20 mL). Organic layers were

combined, washed with water and brine. After drying with $MgSO_4$ followed by filtration, volatiles were distilled off on a rotary evaporator. Finally, the product was purified using column chromatography (SiO_2, c-hexane/$CHCl_3$ = 5/95 *v/v*, R_f =0.44) to obtain a pale brown solid.

Table S 2 Conditions for the mechanochemical synthesis of **1**

<table>
<tr><th>A</th><th>B</th><th>C</th><th>D</th><th>E</th><th>F</th></tr>
<tr><th>entry</th><th>4
(mg; mmol; eq)</th><th>5
(mg; mmol; eq)</th><th>other
(mg; mmol; eq)</th><th>LAG solv.
(µl)</th><th>yield
(%)</th></tr>
<tr><td>1</td><td rowspan="13">14.0; 0.0375; 1.5</td><td rowspan="13">10.0; 0.025; 1.0</td><td>$CuSO_4{\cdot}H_2O$ 12.5; 0.05; 2.0
sodium L-ascorbate 9.9; 0.05; 2.0</td><td rowspan="3">DCM
(25)</td><td>20[a]</td></tr>
<tr><td>2</td><td>$CuSO_4{\cdot}5H_2O$ 12.5; 0.05; 2.0
sodium L-ascorbate 9.9; 0.05; 2.0</td><td>50[c]</td></tr>
<tr><td>3</td><td>CuTC 2.4; 0.013; 0.5
DIPEA 5.0; 0.025; 1.0</td><td>30[a]</td></tr>
<tr><td>4</td><td>CuTC 2.4; 0.013; 0.5
DIPEA 5.0; 0.025; 1.0</td><td></td><td>70[c]</td></tr>
<tr><td>5</td><td>CuI 4.8; 0.025; 1.0
DIPEA 5.0; 0.025; 1.0</td><td rowspan="2">-</td><td>26[a]</td></tr>
<tr><td>6</td><td>$Cu(OAc)_2$ 4.6; 0.025; 1.0
DIPEA 5.0; 0.025; 1.0</td><td>24[a]</td></tr>
<tr><td>7</td><td>$Cu(OAc)_2$ 4.6; 0.025; 1.0
sodium L-ascorbate 5.0; 0.025; 1.0</td><td>DCM
(25)</td><td>33[a]</td></tr>
<tr><td>8</td><td>copper metal mesh 80.0; -; -</td><td>-</td><td>11[a]</td></tr>
<tr><td>9</td><td>copper metal mesh 80.0; -; -
sodium L-ascorbate 5.0; 0.025; 1.0</td><td rowspan="2">DCM
(25)</td><td>35[a]</td></tr>
<tr><td>10</td><td>copper metal mesh 80.0; -; -
sodium L-ascorbate 5.0; 0.025; 1.0</td><td>70[b]</td></tr>
<tr><td>11</td><td>copper metal mesh 80.0; -; -
sodium L-ascorbate 5.0; 0.025; 1.0</td><td>EtOAc
(25)</td><td>32[b]</td></tr>
<tr><td>12</td><td>copper metal mesh 80.0; -; -
sodium L-ascorbate 5.0; 0.025; 1.0
1,10-phenanthroline 0.9; 0.005; 0.2</td><td rowspan="2">DCM
(25)</td><td>48[b]</td></tr>
<tr><td>13</td><td>copper metal mesh 80.0; -; -
sodium L-ascorbate 5.0; 0.025; 1.0</td><td>85[c]</td></tr>
</table>

[a] reaction time – 3 h; [b] reaction time – 6 h; [c] reaction time – 8 h

Notes on mechanochemical reactions with copper metal mesh:

1. Copper metal mesh (**Figure S 1a**) (> 99.95 % Cu) produced by PPH Polskie Odczynniki Chemiczne (now Avantor Performance Materials Poland S.A.)
2. Copper metal mesh was cleaned before use by rinsing with 1M HCl and then with distilled water, acetone, and finally dried in a stream of hot air.
3. The mesh was then cut into 0.5 cm × 0.5 cm square sections (**Figure S 1b**), three such fragments were used for the synthesis with a total mass of 80.0 mg (eight times the weight of amide **5**).

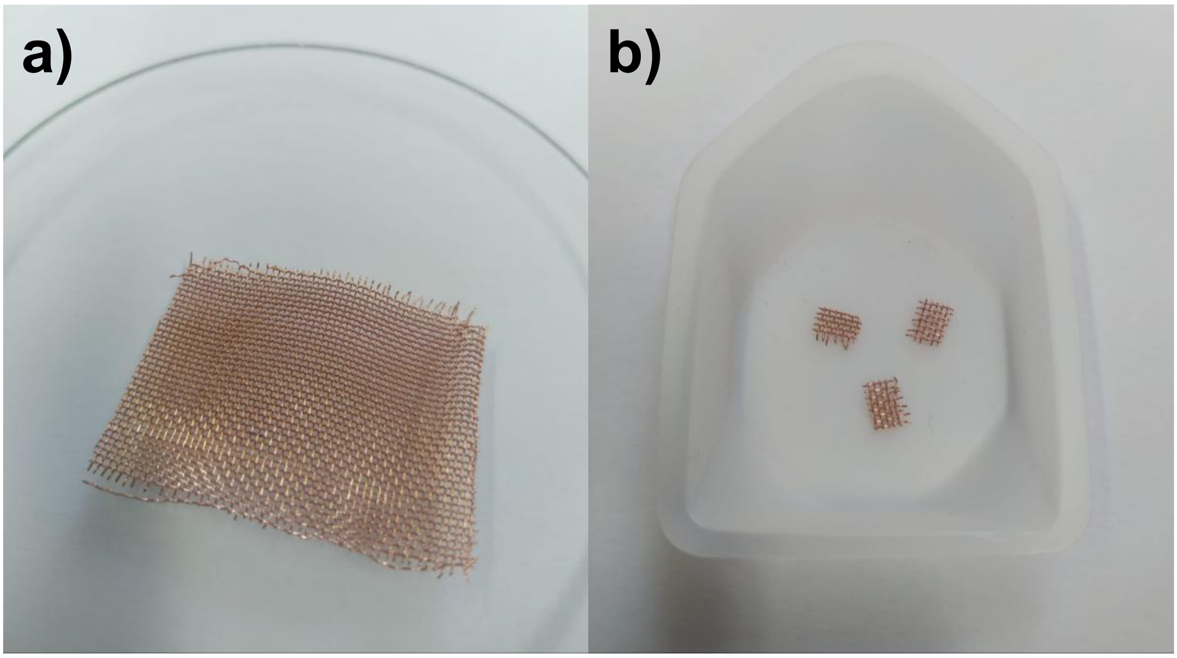

Figure S 1 Copper metal mesh used in mechanochemical syntheses: **a)** whole piece of mesh, **b)** fragments of copper metal mesh used for synthesis.

General procedure for the sonochemical synthesis of **1**:

Into a glass vial ($\phi_{ext.}$: 16 mm, high: 150 mm), compounds **4** (1.5 equiv.) and **5** (1.0 equiv.), together with a copper source (with reductor and/or additional reagents when required; see: **Table S 3**, column D), were placed together with dichloromethane (DCM; 50 µl). The vial was then placed in an ultrasonic bath for 3-6 hours. Dichloromethane (20 mL) was added to remove the solid reaction mixture from the vial. 1M HCl (20 mL) was added, and the crude product was extracted with CH_2Cl_2 (3x20 mL). Organic layers were combined, washed with water and brine. After drying with $MgSO_4$ followed by filtration, volatiles were distilled off on a rotary evaporator. Finally, the product was purified using column chromatography (SiO_2, c-hexane/$CHCl_3$ = 5/95 *v/v*, R_f =0.44) to obtain a pale brown solid.

Table S 3 Conditions for sonochemical synthesis of **1**

A	B	C	D	E
**entry**	**4** (mg; *mmol*; eq)	**5** (mg; *mmol*; eq)	**other** (mg; *mmol*; eq)	**yield** (%)
**1**	14.0; *0.0375*; 1.5	10.0; *0.025*; 1.0	**CuTc** 2.4; *0.013*; 0.5<br>**DIPEA** 5.0; *0.025*; 1.0	**32**[a]
**2**			**$CuSO_4$·$5H_2O$** 12.5; *0.05*; 2.0<br>**sodium L-ascorbate** 9.9; *0.05*; 2.0	**35**[b]
**3**			**CuTc** 2.4; *0.013*; 0.5<br>**DIPEA** 5.0; *0.025*; 1.0	**57**[b]
**4**			**copper metal mesh** 80.0; -; -<br>**sodium L-ascorbate** 5.0; *0.025*; 1.0	**60**[b]

[a] reaction time – 3 h; [b] reaction time – 6 h

S1.2.2 Compound 2

synthesis in solution:

Mixture of **7** (12.6 mg; 0.075 mmol), **5** (18.0 mg; 0,05 mmol), $CuSO_4{\cdot}5H_2O$ (11.4 mg; 0,05 mmol) and sodium L-ascorbate (13.1 mg; 0.07 mmol) in *N*,*N*-Dimethylformamide (DMF; 2 ml) was stirred for 48 hours at room temperature. Distilled water (50 mL) was added, and the formed precipitate was filtered on a nylon membrane (0.45 μm). The resultant solid was dissolved in $CHCl_3$ (45 ml). After drying with $MgSO_4$ followed by filtration, volatiles were distilled off on a rotary evaporator. Finally, the product was purified using column chromatography (SiO_2, n-hexane/$CHCl_3$ = 7/93 *v/v*, R_f =0.67) to obtain pale brown solid (56%).

^{1}H NMR (THF-d_8, 600 MHz, ppm) δH: 9.72 (s,1H), 9.16 (s, 1H), 8.12 – 8.10 (d, 2H), 8.05 – 8.03 (d, 2H) 7.95 (s, 2H), 7.91 (s, 2H), 7.79 – 7.78 (d, 4H), 7,67 – 7.66 (d, 2H) 7.48 – 7.46 (t, 4H), 7.38 – 7.37 (m, 2H), 7.11 – 6.98 (m, 18H);
{^{1}H}^{13}C NMR (THF–d_8, 150 MHz, ppm) δC: 158.6, 145.5, 145.1, 145.1, 145.1, 143.7, 142.8, 142.1, 141.8, 141.8, 140.2, 138.5, 137.4, 132.7, 132.4, 132.3, 129.8, 129.5, 128.7, 128.5, 128.2, 127.3, 127.3, 127.2, 126.5, 125.7125.4, 121.5, 120.1;
ESI-HRMS (TOF): calcd. for $C_{53}H_{38}N_4O_1$ $[M^+H]^+$ m/z = 746.3040, found: 746.3042

General procedure for mechanochemical synthesis of **2**:

Into a stainless-steel jar (volume 1.5 mL) compounds **7** (1.5 equiv.) and **5** (1.0 equiv.) together with copper source (with reductor and/or additional reagents when required) were added. Five stainless steel grinding balls were added (diameter: 3.0 mm) together with dichloromethane (LAG when required; 25 μL). Reaction mixture was ground with frequency of 30 Hz, for 3-8 hours. Dichloromethane (10-15 mL) was added to remove solid reaction mixture from a jar. 1M HCl (10 mL) was added, and the crude product was extracted with CH_2Cl_2 (3x20 mL). Organic layers were combined, washed with water and brine. After drying with $MgSO_4$ followed by filtration, volatiles were distilled off on a rotary evaporator. Finally, the product was purified using column chromatography (SiO_2, n-hexane/$CHCl_3$ = 7/93 *v/v*, R_f =0.67) to obtain pale brown solid.

Table S 4 Conditions for mechanochemical synthesis of **2**

<table>
<tr><th>A</th><th>B</th><th>C</th><th>D</th><th>E</th><th>F</th></tr>
<tr><th>entry</th><th>7
(mg; mmol; <u>eq</u>)</th><th>5
(mg; mmol; <u>eq</u>)</th><th>other
(mg; mmol; <u>eq</u>)</th><th>LAG solv.
(μl)</th><th>yield
(%)</th></tr>
<tr><td>1</td><td rowspan="2">13.0; 0.0375; <u>1.5</u></td><td rowspan="2">10.0; 0.025; <u>1.0</u></td><td>CuTc 2.4; 0.013; <u>0.5</u>
DIPEA 5.0; 0.025; <u>1.0</u></td><td rowspan="2">DCM
(25)</td><td>42[a]</td></tr>
<tr><td>2</td><td>copper metal mesh 80.0; -; <u>-</u>
sodium L-ascorbate 5.0; 0.025; <u>1.0</u></td><td>50[b]</td></tr>
</table>

[a] reaction time – 3 h; [b] reaction time – 6 h

General procedure for the sonochemical synthesis of **2**:

Into a glass vial ($\phi_{ext.}$: 16 mm, high: 150 mm), compounds **7** (1.5 equiv.) and **5** (1.0 equiv.), together with a copper source and reductor; see: **Table S 5**, column D), were placed together with dichloromethane (DCM; 50 μl). The vial was then placed in an ultrasonic bath for 6 hours. Dichloromethane (20 mL) was added to remove the solid reaction mixture from the vial. 1M HCl (20 mL) was added, and the crude

product was extracted with CH_2Cl_2 (3x20 mL). Organic layers were combined, washed with water and brine. After drying with $MgSO_4$ followed by filtration, volatiles were distilled off on a rotary evaporator. Finally, the product was purified using column chromatography (SiO_2, n-hexane/$CHCl_3$ = 7/93 *v/v*, R_f =0.67) to obtain pale brown solid.

Table S 5 Conditions for sonochemical synthesis of **2**

A	B	C	D	E
**entry**	**4** (mg; *mmol*; eq)	**5** (mg; *mmol*; eq)	**other** (mg; *mmol*; eq)	**yield** (%)
**1**	13.0; *0.0375*; 1.5	10.0; *0.025*; 1.0	**copper metal mesh** 80.0; -; -<br>**sodium L-ascorbate** 5.0; *0.025*; 1.0	**40**

S1.2.3 Compound 4

General procedure for synthesis of **4**:

To the flask containing a solution of hydrochloric acid in water (16.0 ml), 4-(1,2,2-triphenylvinyl)aniline (**3**; 1.0 equiv.) was added, followed by sodium nitrite ($NaNO_2$; 9.0 equiv.). The reaction mixture was stirred vigorously for 2 hours. Sodium azide (NaN_3; 4.0 equiv.) was added, and the reaction mixture was stirred for 2 hours. Crude product was extracted with $CHCl_3$ (3x20 ml). Organic layers were combined, washed with brine and water. After drying with $MgSO_4$ followed by filtration, volatiles were distilled off on a rotary evaporator. Finally, the product was purified using column chromatography (SiO_2, n-hexane/$CHCl_3$ = 1/1 *v/v*, R_f =0.8) to obtain a yellow solid (96%).

^{1}H NMR ($CDCl_3$, 600 MHz, ppm) δH: 7.10 – 7,13 (m, 9H), 7.00 – 7.09 (m, 8H), 6.77 (m, 2H);
{^{1}H}^{13}C NMR ($CDCl_3$, 151 MHz, ppm) δC: 143.7, 143.6, 143.6, 141.4, 140.7, 140.0, 138.1, 132.9, 131.4, 131.4, 131.4, 127.9, 127.8, 127.8, 126.7, 126.6, 118.5;
ESI-HRMS (TOF): calcd. for $C_{26}H_{19}N_3$ $[M^+H]^+$, m/z= 373.1575, found: 373,15723

Table S 6 Conditions for synthesis of **4**

A	B	C	D	D	E
**entry**	**7** (mg; *mmol*; eq)	**HCl** conc. (µl; *mmol*; eq)	**other** (mg/µl; *mmol*; eq)	**temp.** (°C)	**yield** (%)
**1**	100.0; *0.146*; 1.0	110.0; *2.592*; 9.0	**$NaNO_2$** 181.4; *2.260*; 9.0<br>**NaN_3** 76.0; *1.152*; 4.0	RT	**58**
**2**		420.0; *2.592*; 18.0	**$NaNO_2$** 181.4; *2.260*; 9.0<br>**NaN_3** 76.0; *1.152*; 4.0		**62**
**3**		550.0; *2.592*; 22.5	**$NaNO_2$** 181.4; *2.260*; 9.0<br>**NaN_3** 76.0; *1.152*; 4.0	0°C → RT	**67**
**4**		1100.0; *2.592*; 45.0	**$NaNO_2$** 181.4; *2.260*; 9.0<br>**NaN_3** 76.0; *1.152*; 4.0		**96**

S1.2.4 Compound 5

General procedure for the synthesis of **1** in solution:

Mixture of propiolic acid (1.0 equiv.), and *N,N'*-dicyclohexylcarbodiimide (DCC; 1.0 equiv.) in dichloromethane (DCM; 2.0 ml) were stirred for 20 minutes under argon atmosphere in ice bath. Then

solution of 4-(1,2,2-triphenylvinyl)aniline (**3**; 1.0 equiv.) and *N*-hydroxysuccinimide (NHS; 1.0 equiv.) in dichloromethane (DCM; 2.0 ml) was added and reaction mixture was stirred at room temperature. 1M HCl (10 mL) was added, and the crude product was extracted with dichloromethane (CH_2Cl_2; 3x20 mL). Organic layers were combined, washed with water and brine. After drying with $MgSO_4$ followed by filtration, volatiles were distilled off on a rotary evaporator. Finally, the product was purified using column chromatography (Al_2O_3, n-hexane/dichloromethane = 2/9 *v/v*) (R_f = 0.45) to obtain **5** as pale yellow solid (80%).

^{1}H NMR (DMSO-d_6, 600 MHz, ppm) δH: 10.73 (s, 1H), 7.35 – 7.33 (m, 2H), 7.15 – 7.10 (m, 9H), 6.98 – 6.94 (m, 7H), 6.90 – 6.89 (m, 2H), 4.37 (s, 1H);
{^{1}H}^{13}C NMR (DMSO -d_6, 150 MHz, ppm) δC: 149.5, 143.2, 143.2, 143.1, 140.4, 140.0, 139.1, 136.5, 131.1, 130.6, 130.6, 130.6, 127.9, 127.8, 127.8, 126.6, 126.5, 119.0, 78.3, 77.1;
ESI-HRMS (TOF): calcd. for $C_{29}H_{21}N_1O_1$ $[M^+H]^+$ m/z = 400.1698, found: 400.1696

Table S 7 Conditions for synthesis of **5** in solution

A	B	C	D	E	E
**entry**	**3**<br>(mg; *mmol*; eq)	**propiolic acid**<br>(mg; *mmol*; eq)	**other**<br>(mg; *mol*; eq)	**time**<br>(h)	**yield**<br>(%)
**1**	50.0; *0.146*; 1.0	10.9; *0.146*; 1.0	**DCC** 32.1; *0.146*; 1.0<br>**NHS** 17.9; *0.146*; 1.0	48	**80**
**2**	100.0; *0.288*; 1.0	20.2; *0.288*; 1.0	**DCC** 59.4; *0.288*; 1.0<br>**NHS** 33.1; *0.288*; 1.0	72	**70**

General procedure for mechanochemical synthesis of **5**:

Into a stainless-steel jar (volume 1.5 mL) compounds **3** (1.0 equiv.) and propiolic acid (4.0 equiv.) together with coupling agents were added. Five stainless steel grinding balls were added (diameter: 3.0 mm) together with LAG solvent. Reaction mixture was ground with frequency of 30 Hz. Dichloromethane (10-15 mL) was added to remove solid reaction mixture from a jar. 1M HCl (10 mL) was added, and the crude product was extracted with dichloromethane (CH_2Cl_2; 3x20 mL). Organic layers were combined, washed with water and brine. After drying with $MgSO_4$ followed by filtration, volatiles were distilled off on a rotary evaporator. Finally, the product was purified using column chromatography (Al_2O_3, n-hexane/dichloromethane = 2/9 *v/v*) (R_f = 0.45) to obtain **5** as pale yellow solid (36%).

Table S 8 Conditions for mechanochemical synthesis of **5**

A	B	C	D	E	F	G
**entry**	**3**<br>(mg; *mmol*; eq)	**propiolic acid**<br>(mg; *mmol*; eq)	**other**<br>(mg; *mmol*; eq)	**LAG solv.**<br>(µl)	**time** (h)<br>**temp.**	**yield**<br>(%)
**1**	20.0; *0.0576*; 1.0	16.1; *0.23*; 4.0	**EDC·HCl** 8.9; *0.0576*; 1.0	**EtOAc**<br>(25)	1h<br>RT	**21**
**2**				**DCM**<br>(10)	2h<br>RT	**21**
**3**	50.0; *0.146*; 1.0	10.9; *0.146*; 1.0	**DCC** 32.1; *0.146*; 1.0<br>**NHS** 17.9; *0.146*; 1.0	**DCM**<br>(15)	3h<br>RT	**36**

S1.2.5 Compound 7

General procedure for synthesis of **7**:

To the flask containing solution of acid in water, 5'-phenyl-[1,1':3',1"-terphenyl]-4-amine (**6**; 1.0 equiv.) was added, followed by sodium nitrite ($NaNO_2$; 9.0 equiv.). The reaction mixture was stirred vigorously for 1 or 2 hours. Sodium azide (NaN_3; 4.0 equiv.) was added and the reaction mixture was stirred for 1 or 2 hours. Crude product was extracted with $CHCl_3$ (3x20 ml). Organic layers were combined, washed with brine and water. After drying with $MgSO_4$ followed by filtration, volatiles were distilled off on a rotary evaporator. Finally, the product was purified using column chromatography (SiO_2, n-hexane/$CHCl_3$ = 4/6 *v/v*, R_f =0.8) to obtain yellow solid (93%).

^{1}H NMR (600 MHz, $CDCl_3$, ppm) δH: 7.78 (d, 1H), 7.75 (d, 2H), 7.73-7.67 (m, 6H), 7.51-7.46 (m, 4H), 7.41 (m, 2H), 7.17-7.11 (m, 2H)
{^{1}H}^{13}C NMR ($CDCl_3$, 151 MHz, ppm) δC: 142.5, 141.2, 141.0, 139.4, 137.9, 129.5, 128.9, 128.6, 128.1, 127.6, 127.3, 125.3, 124.8, 119.5
ESI-HRMS (TOF): calcd. for $C_{24}H_{17}N_3$ $[M^+H]^+$ m/z = 348.14952, found: 348.14949

Table S 9 Conditions for synthesis of **7**

A	B	C	D	E	F	G
**entry**	**6** (mg; *mmol*; eq)	**acid** (mg/µl; *mmol*; eq)	**H_2O** (ml)	**other** (mg/µl; *mmol*; eq)	**time** (h) **temp.**	**yield** (%)
**1**	32.1; *0.1*; 1.0	**p-TsOH** 155.0; *0.9*; 9.0	5.0	**$NaNO_2$** 7.0; *0.9*; 9.0 **NaN_3** 13.0; *0.2*; 2.0	1h + 1h RT	**45**
**2**					1h + 1h 0°C	**5**
**3**					1h + 1h 0°C → RT	**21**
**4**					2h + 2h 0°C → RT	**50**
**5**		**HCl** conc. 90.0; *0.9*; 9.0			2h + 2h RT	**88**
**6**	160.7; *0.5*; 1.0	**HCl** conc. 450.0; *4.5*; 9.0	25.0	**$NaNO_2$** 34.5; *4.5*; 9.0 **NaN_3** 65.0; *1.0*; 2.0		**78**
**7**	100.0; *0.31*; 1.0	**HCl** conc. 280.0; *2.8*; 9.0	16.0	**$NaNO_2$** 21.4; *4.5*; 9.0 **NaN_3** 81.0; *1.2*; 4.0		**93**

S1.3 Aggregation-induced emission (AIE) studies – preparation of the samples

The studies on the aggregation induced emission (AIE) behaviour were performed employing measurements of the fluorescence spectra. The experiments were performed in the H_2O/THF solvent mixtures. Stock solutions of **1** and **2** ($2{\cdot}10^{-3}$ M) in THF were diluted with proper volume of pure THF followed by addition of H_2O to reach given vol% of H_2O in the sample.

S1.4 Estimation of fluorescence quantum yield

The measurements for the estimation of fluorescence quantum yields (Φ_F) for the **1** and **2**, as well as their aggregates were performed at room temperature according to the literature procedures.[2,3] Fluorescence quantum yields (Φ_F) were determined by comparison with quinine sulfate (QS) in 0.5M H_2SO_4 ($\Phi_{F,ref}$ = 0.5)[2] as the standard. The measurements were performed with highly diluted solutions, for which absorbance (A) values for the highest wavelength were not higher than 0.1 a.u. The excitation wavelengths (λ_{ex}) for each sample were selected on the basis of the UV-vis spectra, and were as follows:

- λ_{ex} = 320 nm for **1**,
- λ_{ex} = 320 nm for **2**,

Concentrations were as follows: $C_{QS} = 2{\cdot}10^{-6}$ M; $C_{\mathbf{molecule/aggregates}} = 2{\cdot}10^{-6}$ M.

The following formula was used for the calculation of Φ_F:

$$\phi_{\mathrm{F}} = \phi_{\mathrm{F,ref}} \cdot \frac{F_{sample}}{F_{reference}} \cdot \frac{1-10^{-A_{\mathrm{ref}}}}{1-10^{-A_{\mathrm{sample}}}} \cdot \frac{n_{sample}^2}{n_{reference}^2}$$

where $\Phi_{F,ref}$ is the quantum yield for QS (0.551), F is the integrated area under the fluorescence spectra, A is the absorbance at the excitation wavelength, *n* is the refractive index of the solvent (1.346 for 0.5M H_2SO_4, 1.4072 for THF, *n* for the aggregates solution in the given volume ratio was taken as weighted arithmetic mean with weights equal to vol% of H_2O and THF in the mixture).

1.5 Receptor studies – titration experiments methodology

The anion binding experiments between **1** and **2** (receptors) and cations (analytes; F^-, Cl^-, Br^-, I^-, NO_3^-, $H_2PO_4^-$, SO_4^{2-} in form of tetrabutylammonium ($[N(C_4H_9)_4]^+$) salts) were performed employing the fluorescence spectra titration experiments. The experiments were performed in the H_2O/THF = 95:5 *v/v* system as follows. Stock solution of **1** or **2** ($2{\cdot}10^{-3}$M) in THF was diluted with adequate volume of pure THF and H_2O to reach the final sample volume of 3 ml and the desired composition of solvents. The given anion was introduced to the mixture in the form of H_2O/THF = 95:5 *v/v* solutions. Each titration experiment consisted of 15 steps. First, fluorescence of a solution containing only a receptor was measured, then solutions containing given cation were added in 14 consecutive steps to achieve the following proportions of cation to receptor: 0.00, 0.15, 0.25, 0.4, 0.5, 0.65, 0.75, 0.85, 1.0, 2.0, 3.0, 4.0, 5.0, 10.0, 30.0 equiv. To ensure proper mixing, before the measurement of the spectrum, the contents of the cuvette were well-mixed using a magnetic stirrer (1200 rpm).

For the studies using buffer solutions, buffers of appropriate pH were used instead of distilled water and the titration experiments were performed as described above. The following buffers were used: pH 5.1 – MES buffer (C = 0.01 M), pH 7.4 – PBS buffer (C = 0.01 M), pH 8.2 – Tris buffer (C = 0.01 M).

The limit of detection (LOD) value for each system was calculated using a linear plot: $(I - I_{\min})/(I_{\max} - I_{\min}) = f\ (\log(C))$. The x value for y =1 was calculated (value x(y=1)), and then LOD was taken as $10^{x(y=1)}$.

1.6 Sensors preparation and EMF measurements

The method of the membranes preparation was the same as for standard ion-selective electrodes. The membranes contained: 1% wt receptor (compounds **1**-**2**), 65-66% wt plasticizer (o-NPOE), 32-33% wt PVC and 10% mol (vs receptor) TDMAC (lipophilic additive). The membrane components (200 mg in total) were dissolved in 1.5 ml of THF. The solution was poured into a glass ring placed on a glass. After solvent evaporation, membrane discs of appropriate size were cut off and mounted in electrode bodies (type IS 561, Philips) for electromotive force (EMF) measurements. NaCl solution (0.01 M) was used as an internal filling; the electrodes were conditioned overnight in NaCl solution (0.001 M). For each membrane composition at least three sensor specimens were prepared.

All measurements were carried out with cells of the following type: Ag, AgCl; KCl 1 M / CH_3COOLi 1M / sample solution // membrane // internal filling solution; AgCl, Ag.

S2. NMR spectra

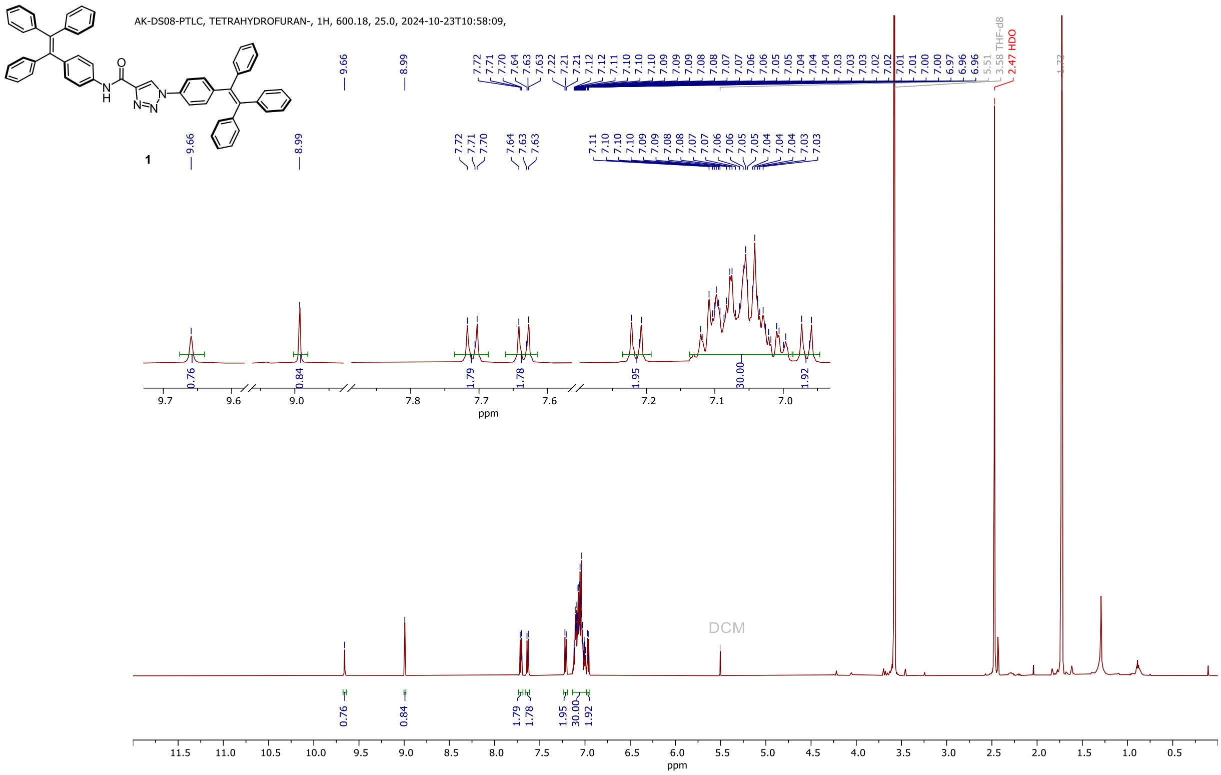

Figure S 2 ^{1}H NMR spectrum (600 MHz, THF-d_8) of compound **1**

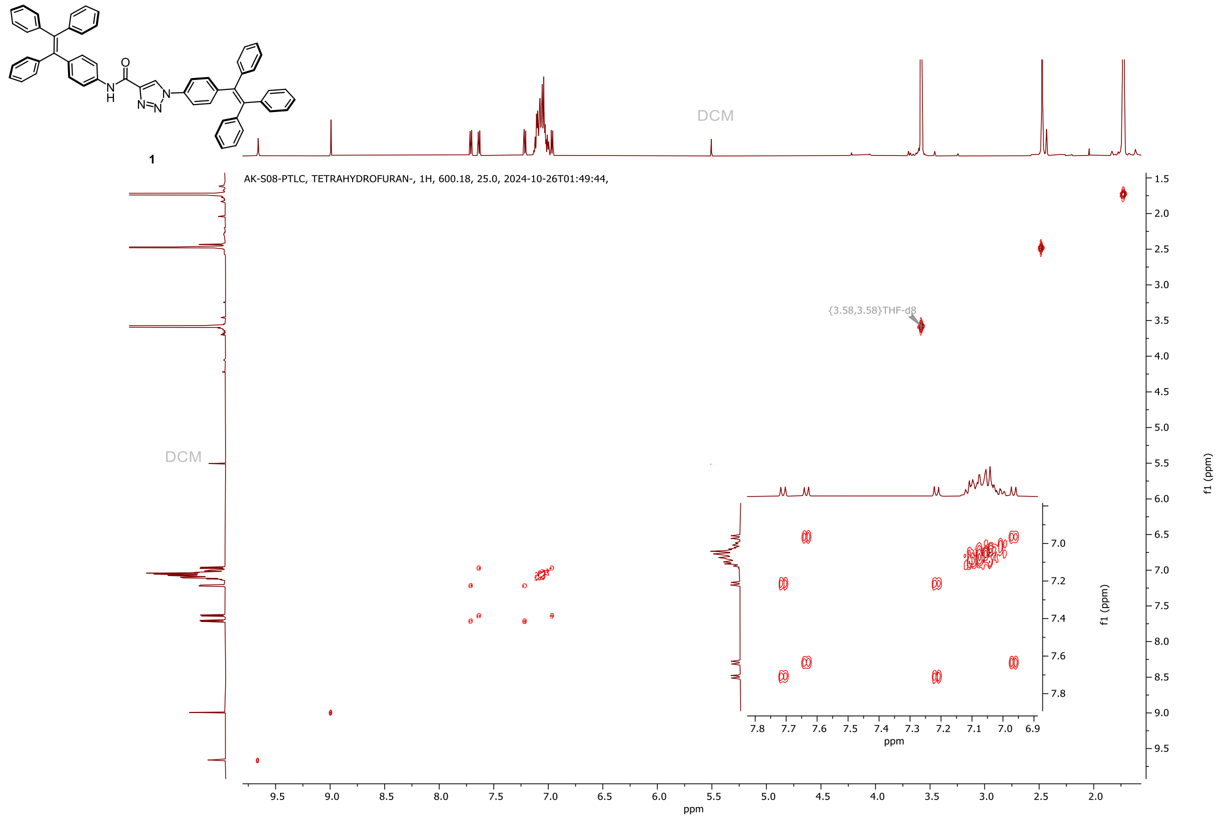

Figure S 3 ^{1}H-^{1}H COSY NMR spectrum (600 MHz, THF-d_8) of compound **1**

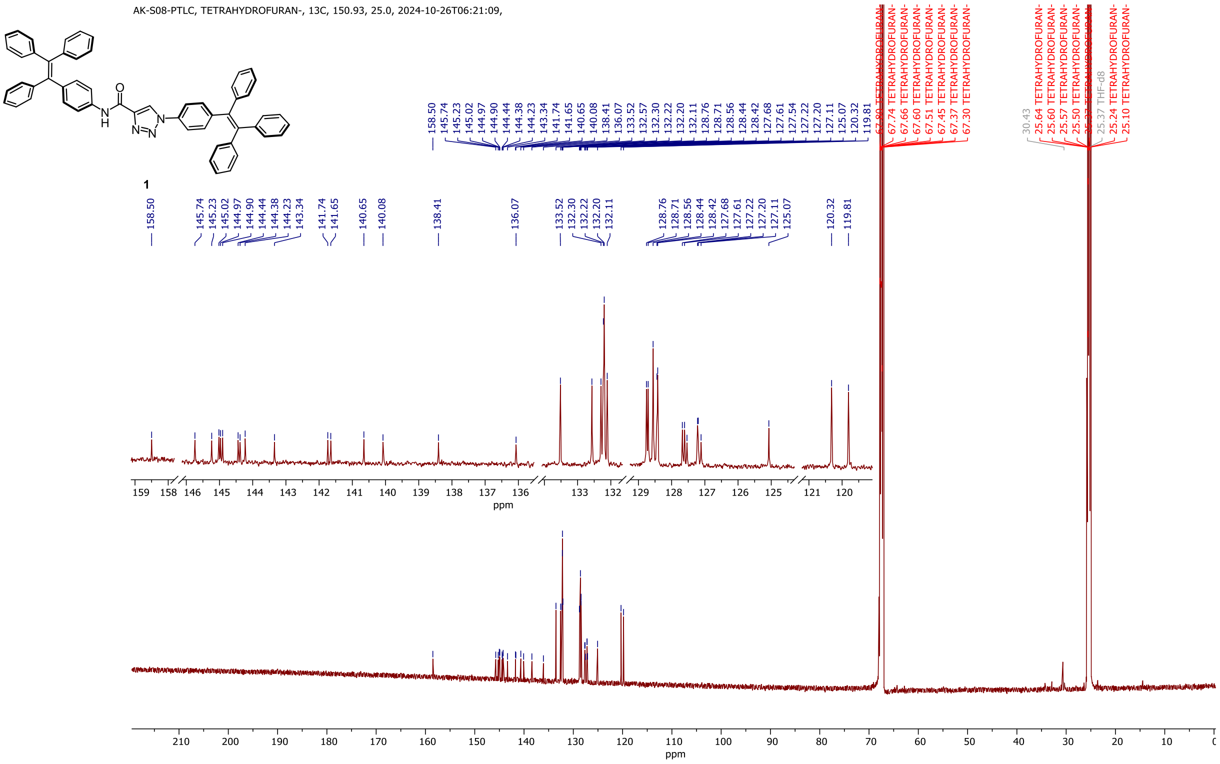

1

Figure S 4 {^{1}H}^{13}C NMR spectrum (151 MHz, THF-d_8) of compound **1**

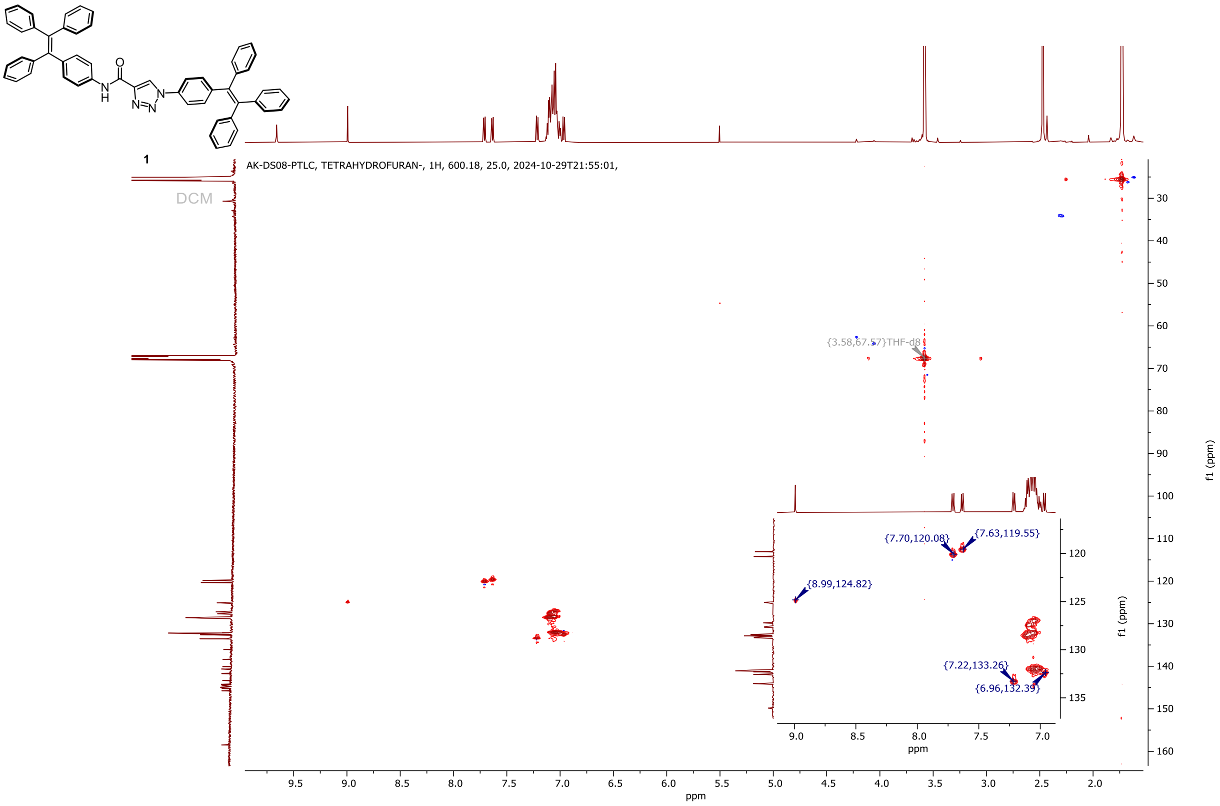

1

Figure S 5 ^{1}H- ^{13}C HSQC (THF-d_8) of compound **1**

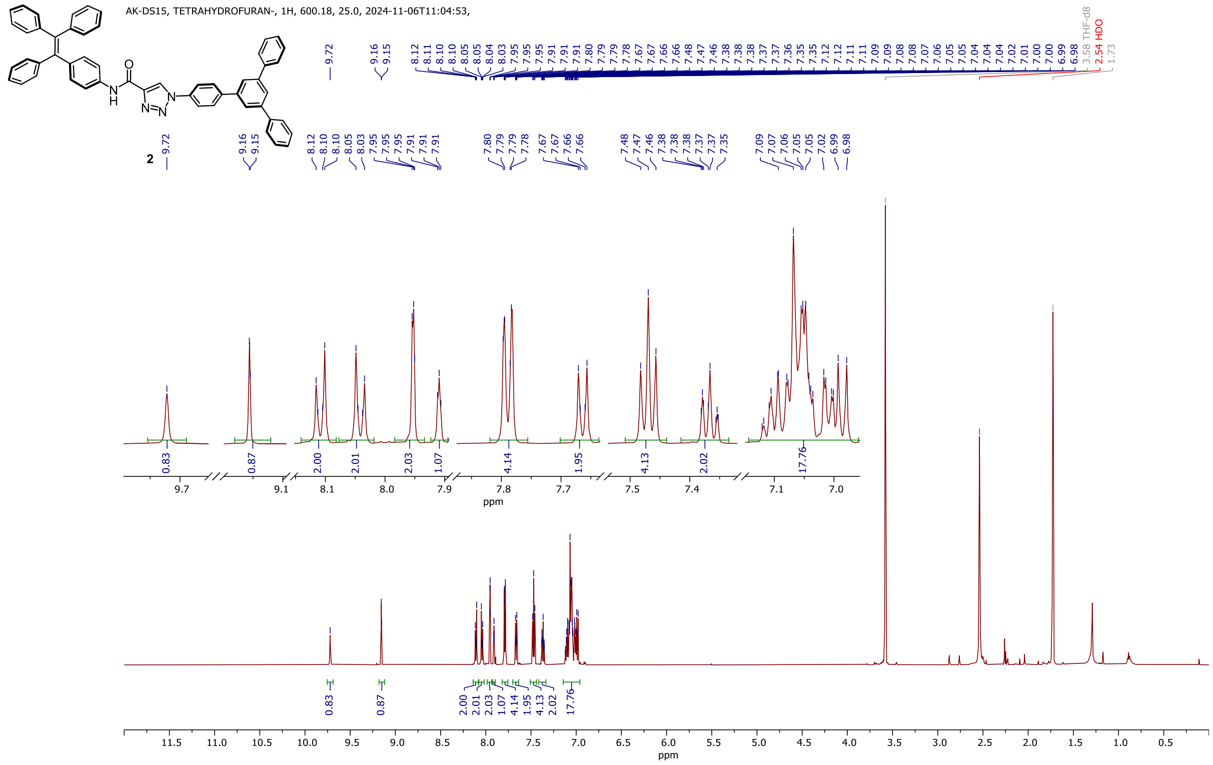

Figure S 6 ^{1}H NMR spectrum (600 MHz, THF-d_8) of compound **2**

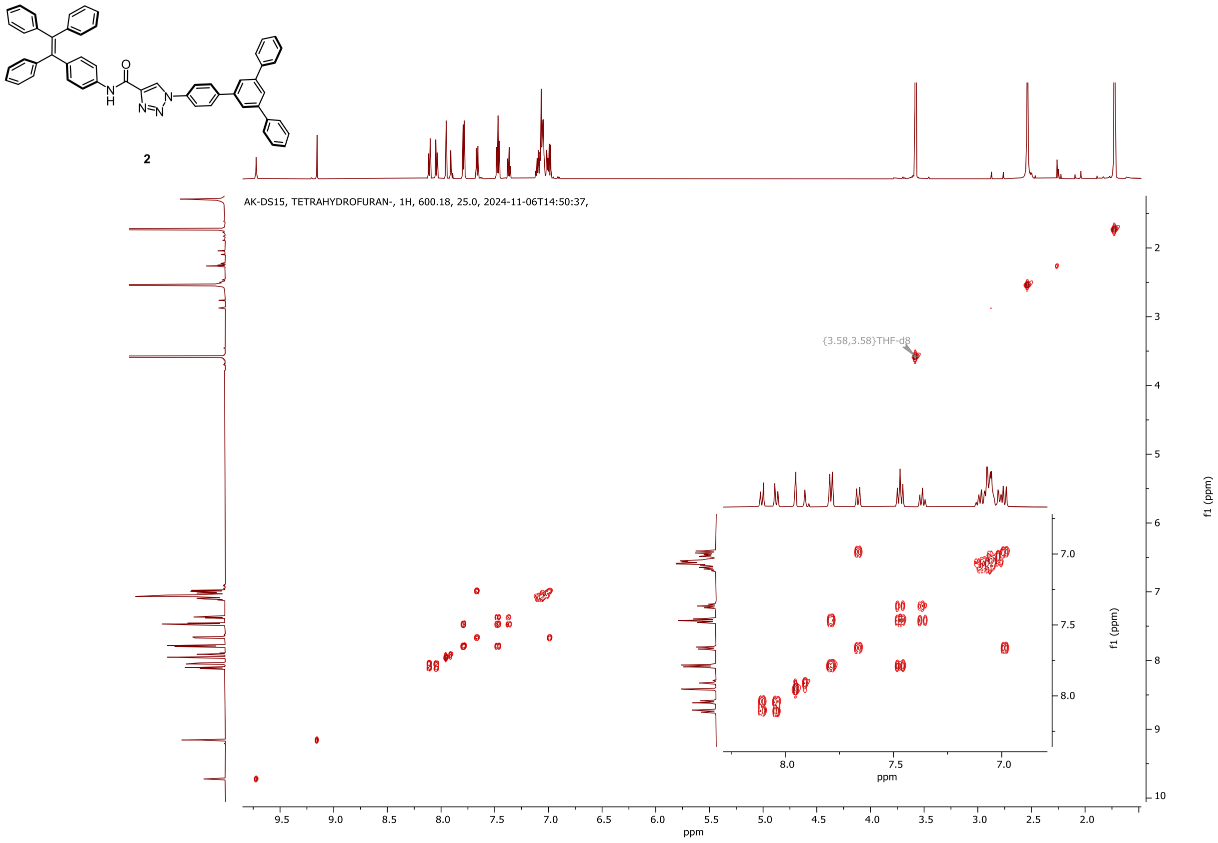

Figure S 7 ^{1}H-^{1}H COSY NMR spectrum (600 MHz, THF-d_8) of compound **2**

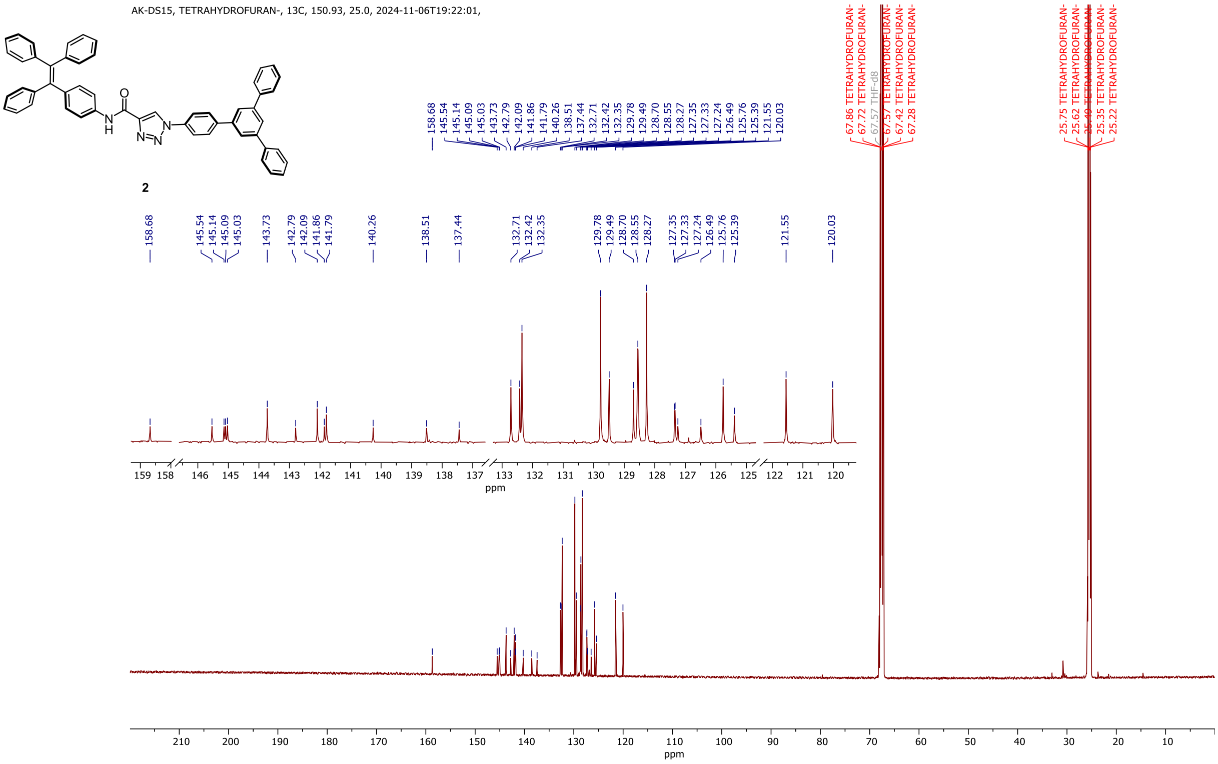

2

Figure S 8 $\{^1H\}^{13}C$ NMR (151 MHz, THF-d_8) of compound **2**

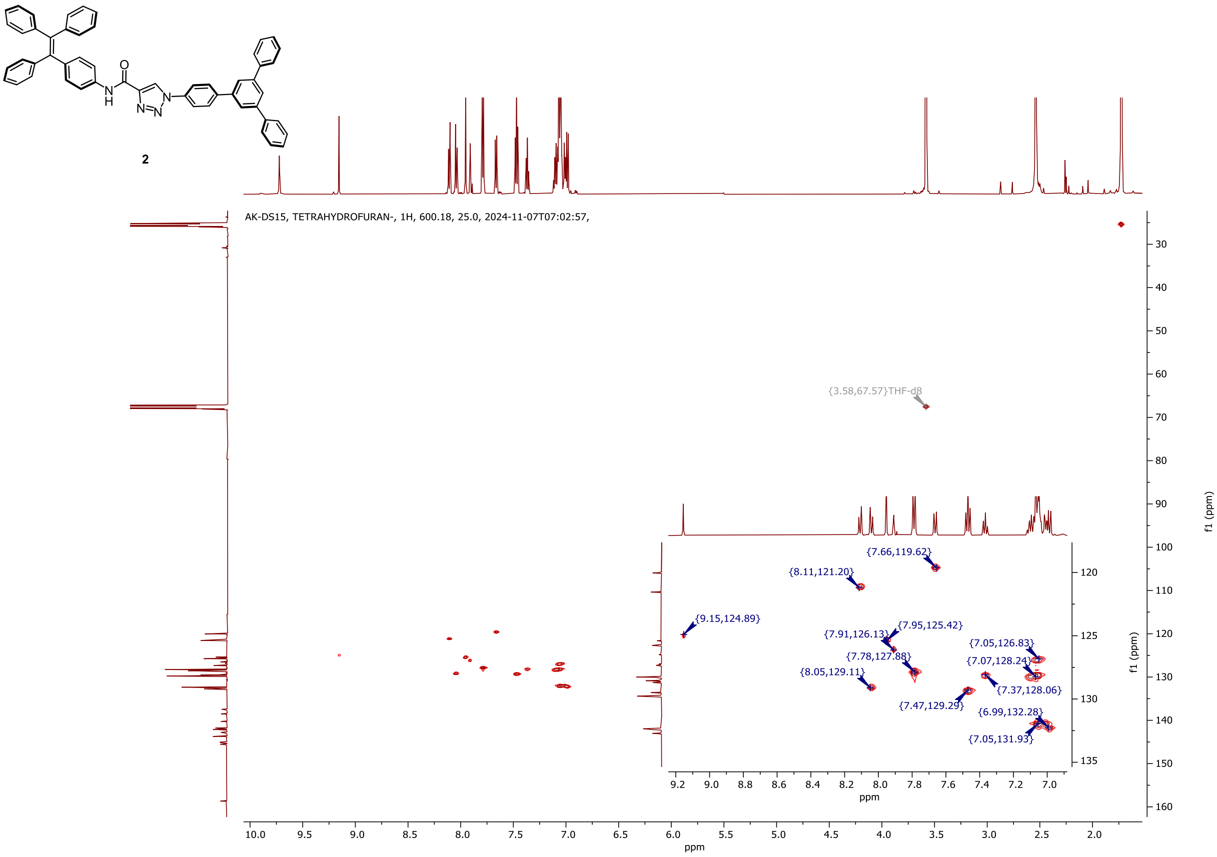

2

Figure S 9 ^1H- ^{13}C HSQC (THF-d_8) of compound **2**

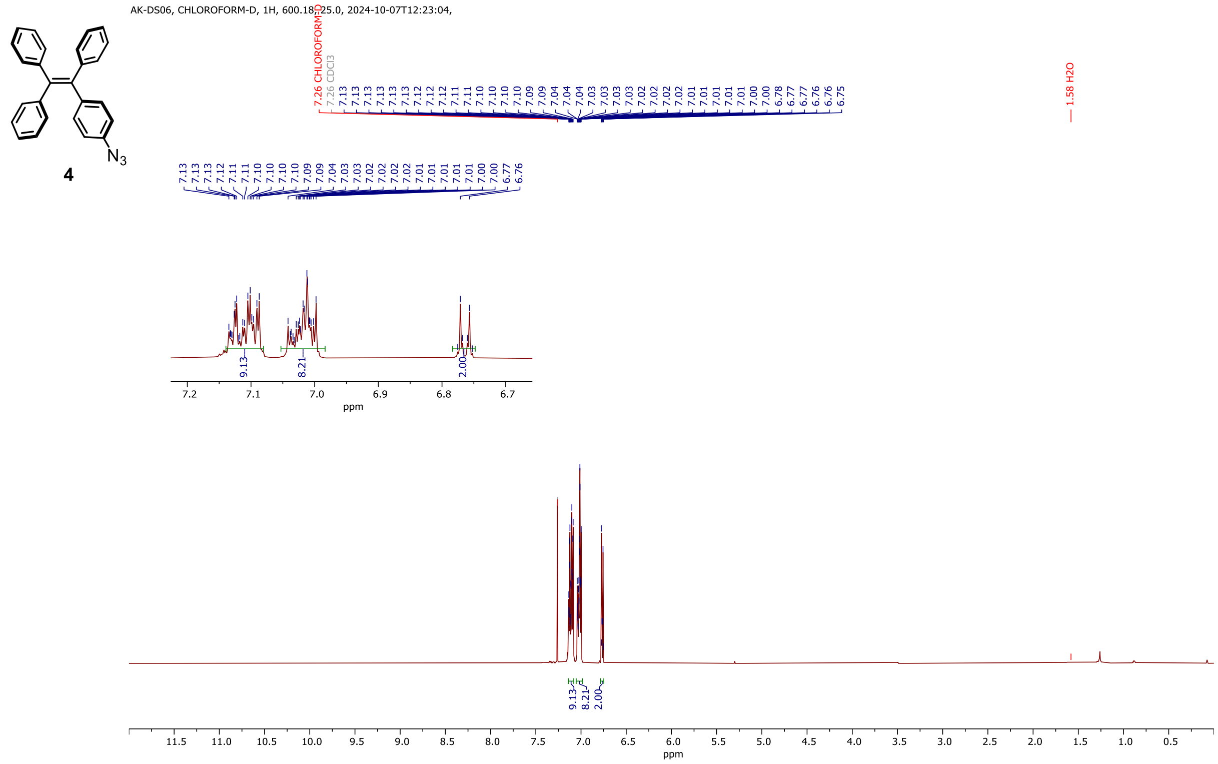

Figure S 10 ^{1}H NMR spectrum (600MHz, $CDCl_3$) of compound **4**

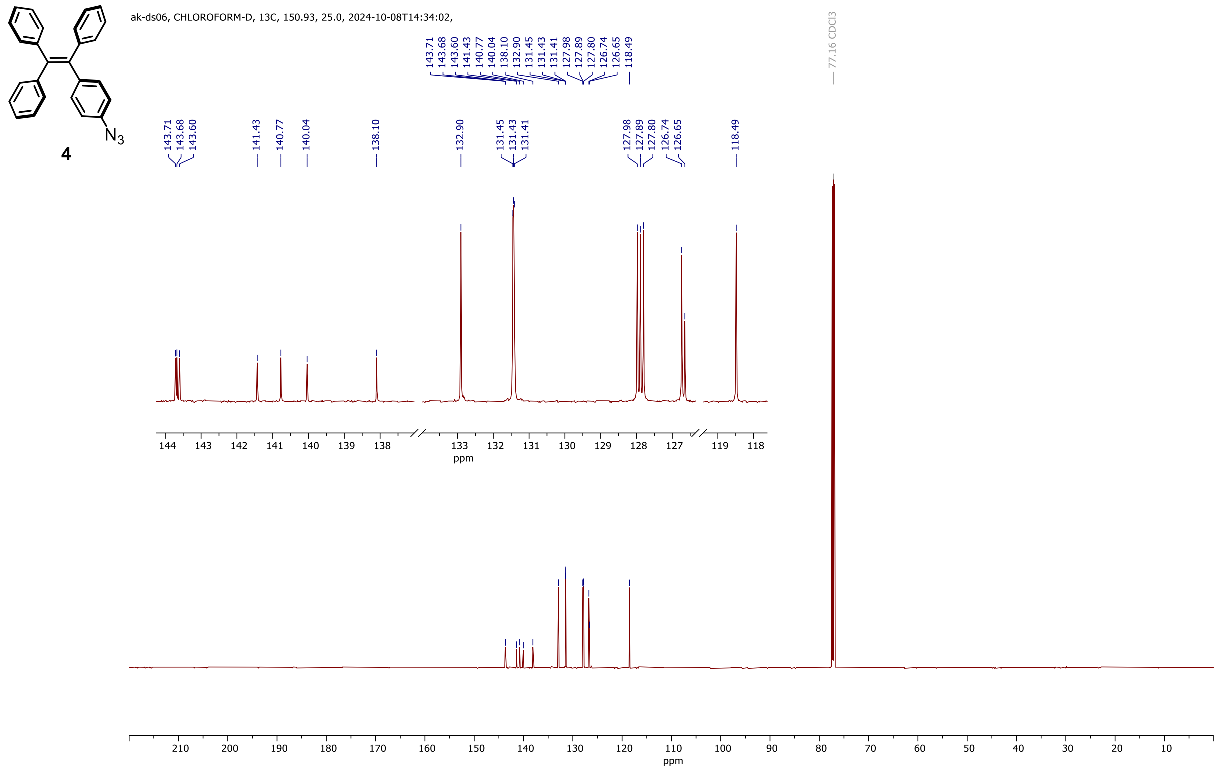

Figure S 11 ^{13}C NMR (151 MHz, $CDCl_3$) of compound **4**

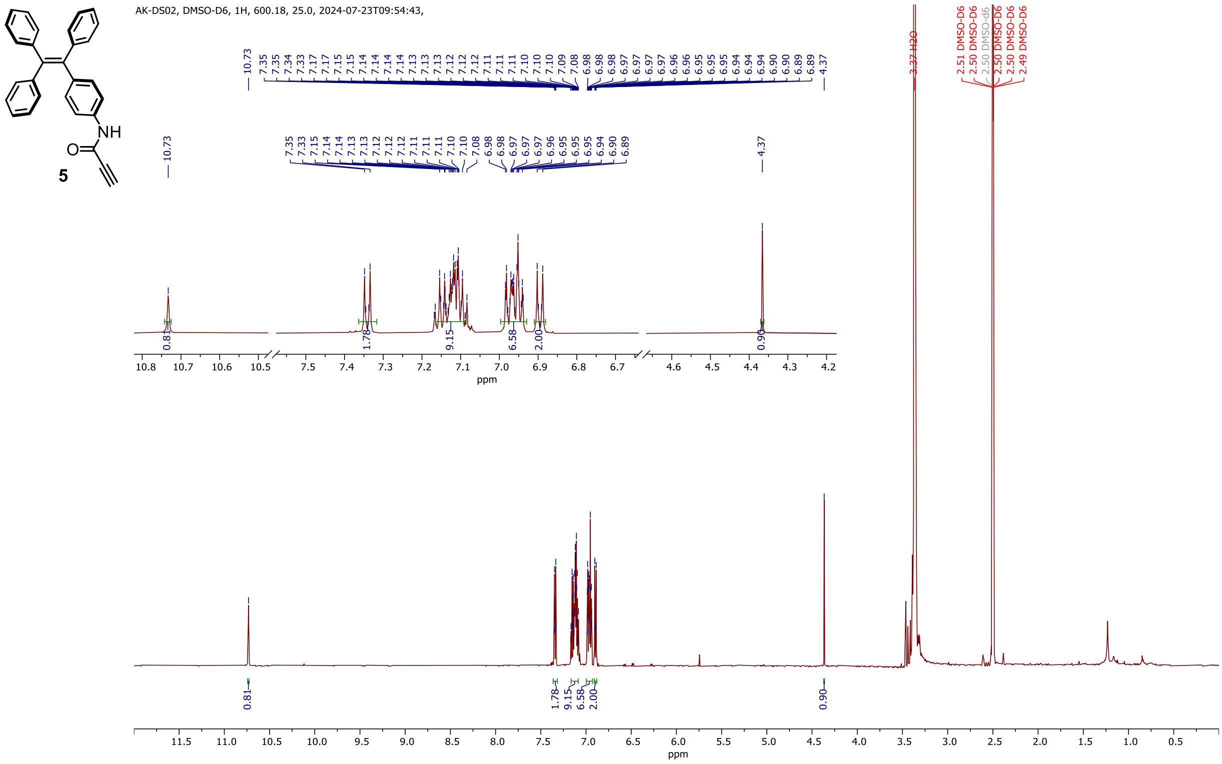

Figure S 12 ^{1}H NMR spectrum (600MHz, DMSO-d_6) of compound **5**

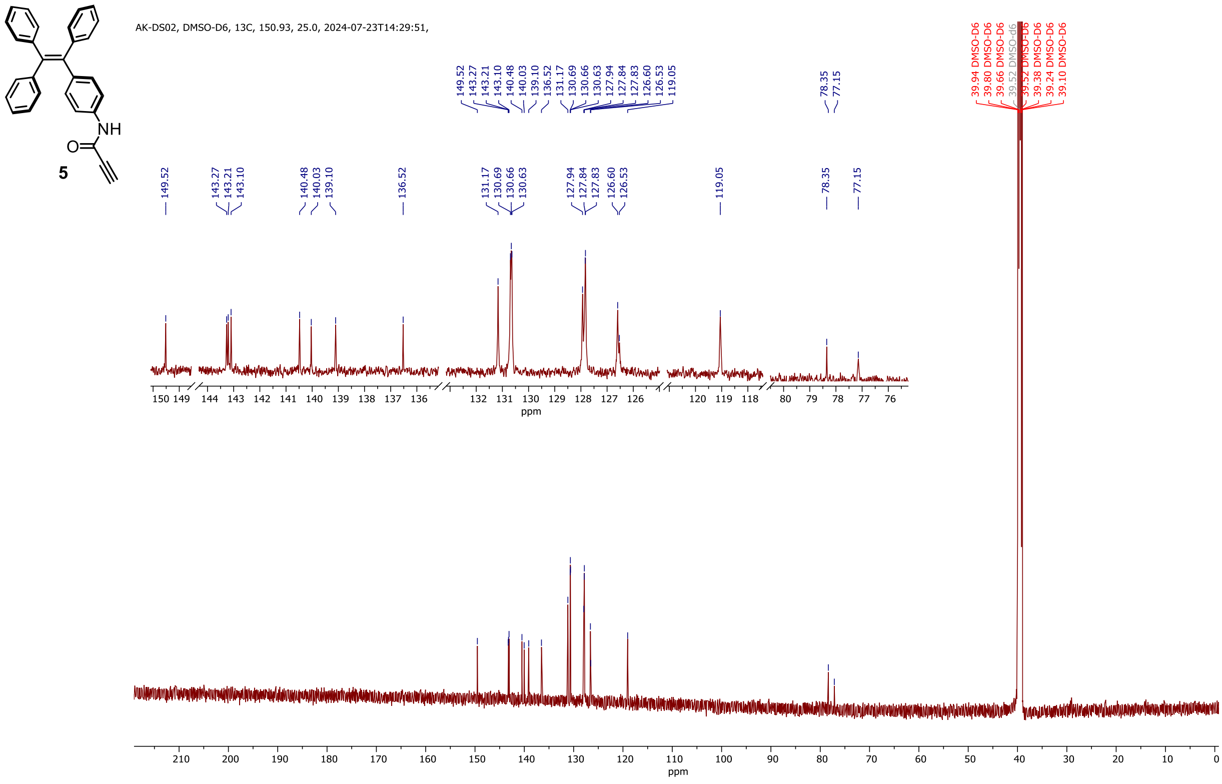

Figure S 13 ^{13}C NMR (151 MHz, DMSO-d_6) of compound **5**

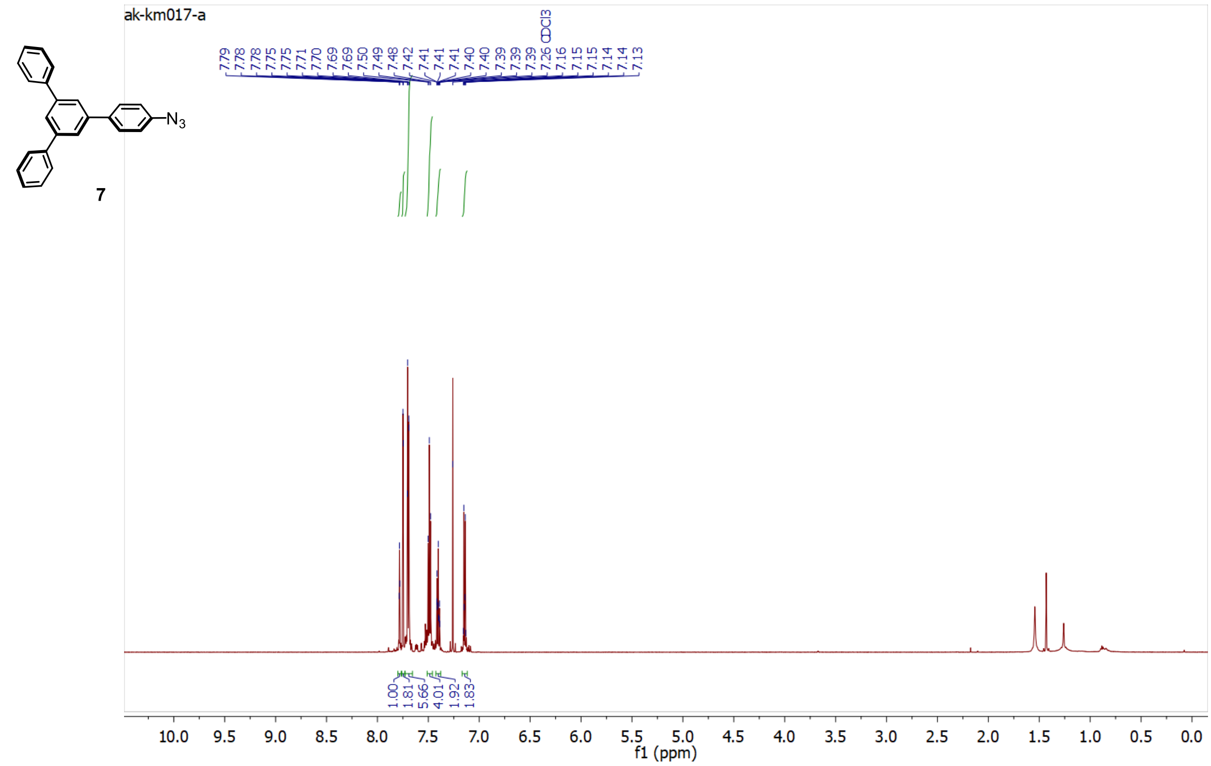

Figure S 14 ^{1}H NMR spectrum (600MHz, $CDCl_3$) of compound **7**

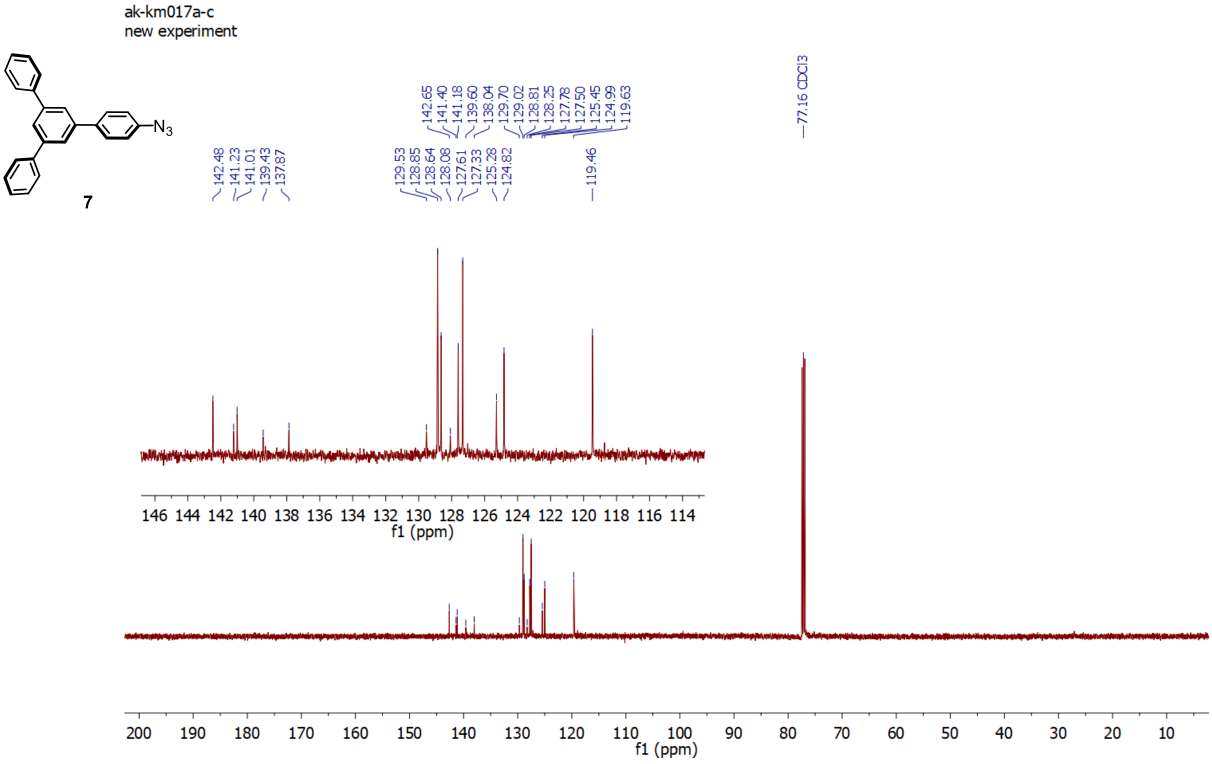

Figure S 15 ^{13}C NMR (151 MHz, $CDCl_3$) of compound **7**

S3. HRMS spectra

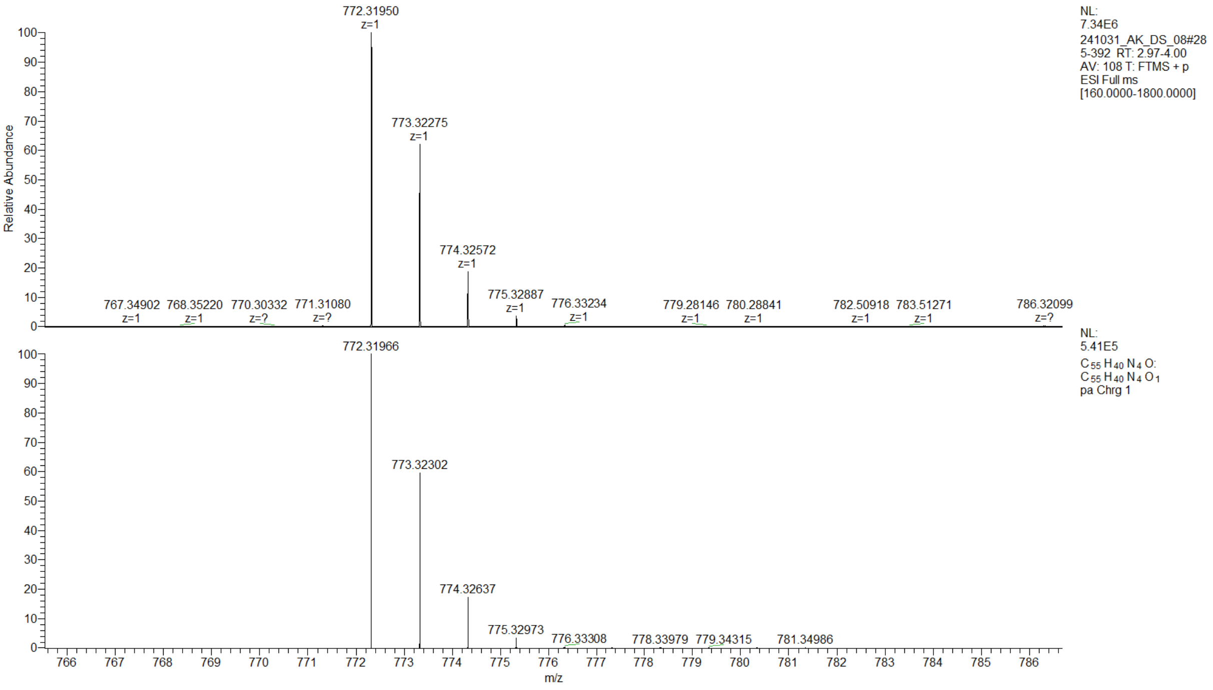

Figure S 16 ESI-HRMS (TOF) spectrum of compound **1**

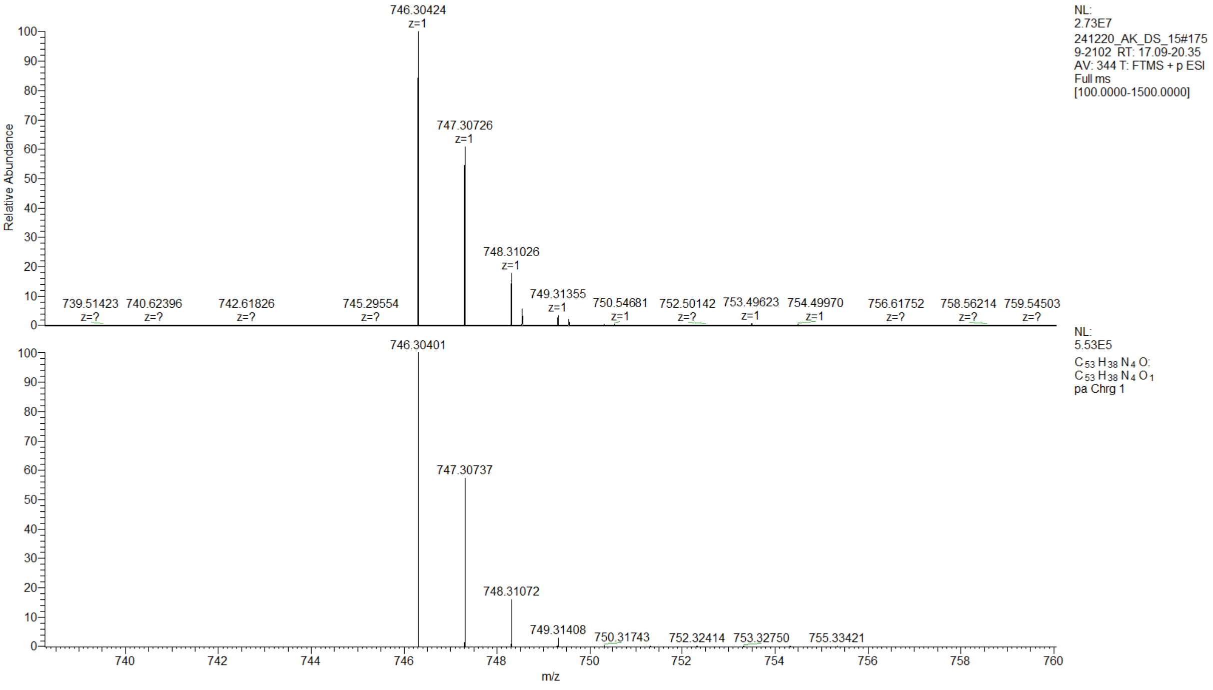

Figure S 17 ESI-HRMS (TOF) spectrum of compound **2**

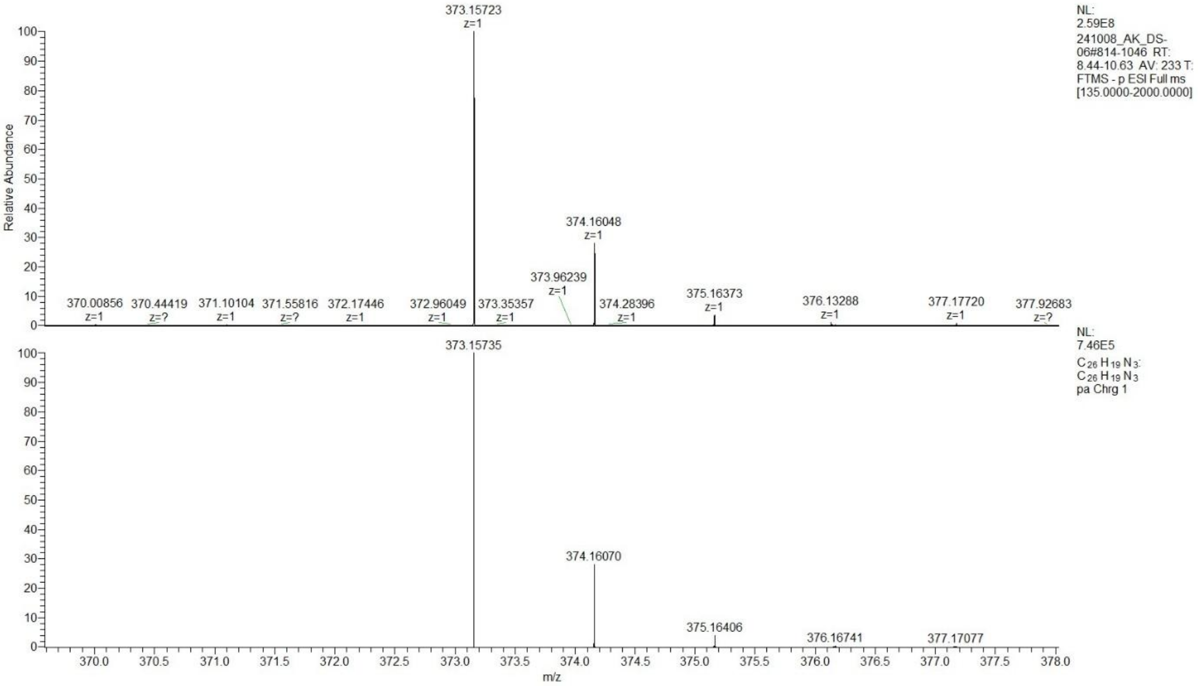

Figure S 18 ESI-HRMS (TOF) spectrum of compound **4**

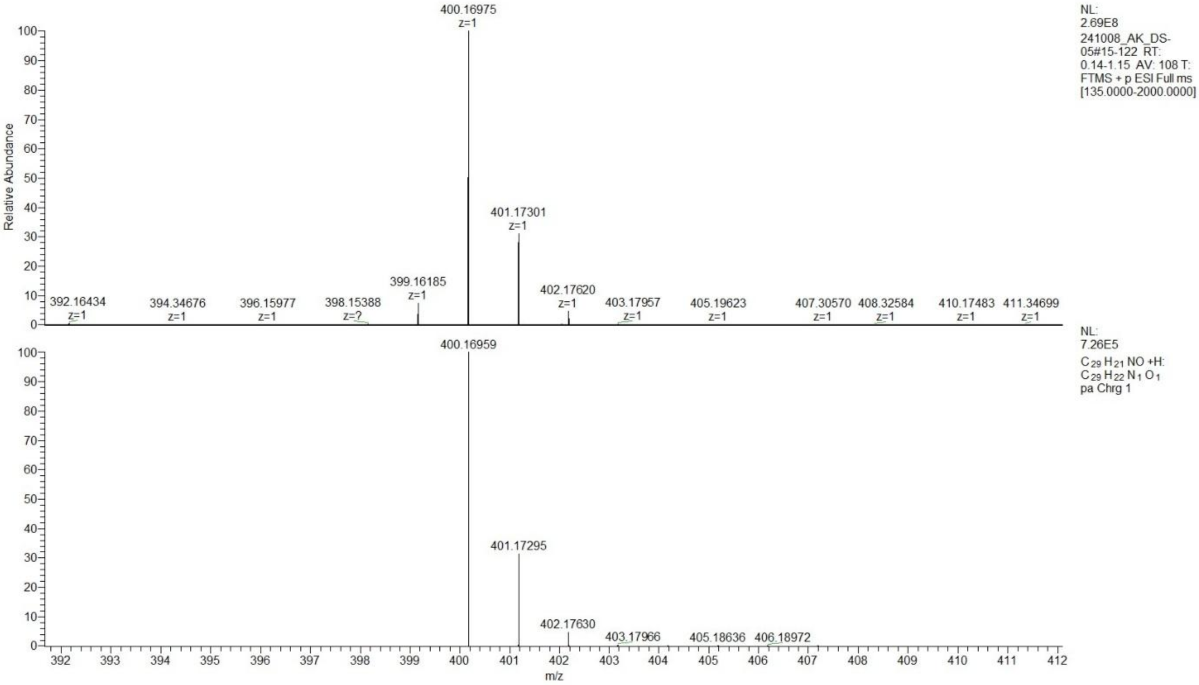

Figure S 19 ESI-HRMS (TOF) spectrum of compound **5**

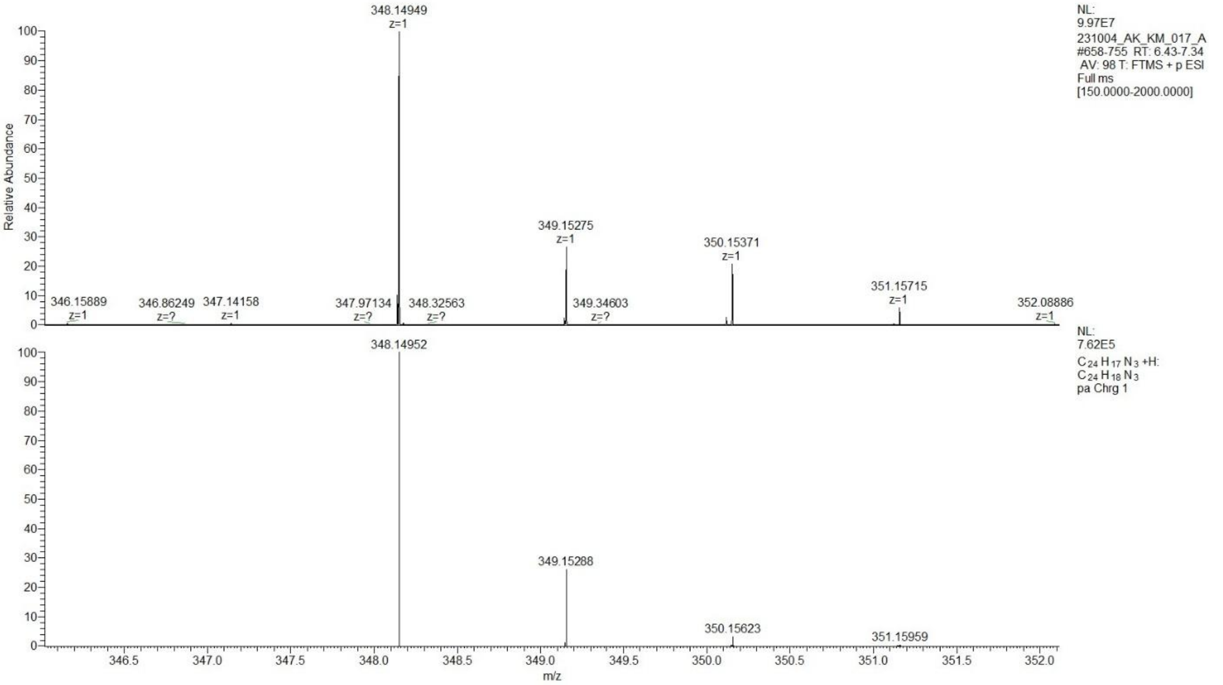

Figure S 20 ESI-HRMS (TOF) spectrum of compound **7**

S4. DFT computations

Density functional theory (DFT) computations regarding structure optimization were performed with Gaussian software[4] with B3LYP functional[5] and the 6-31g(d,p) basis[6] set. The initial structures of molecules were edited in GaussView software[7], and then subjected to calculation. After structure optimization, vibrational frequencies were calculated. All optimized compounds were stable geometric structures as no detected imaginary frequencies were found. Electrostatic surface potential (ESP) maps were generated and analysed using the GaussView software.

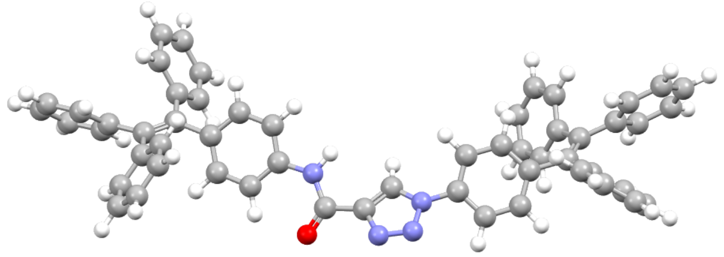

Figure S 21 DFT optimized (B3LYP/6-31g(d,p)) structure of **1**.

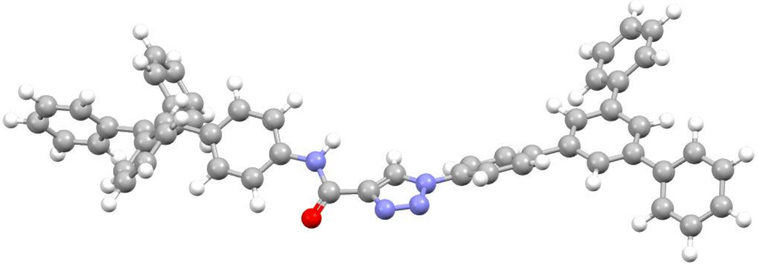

Figure S 22 DFT optimized (B3LYP/6-31g(d,p)) structure of **2**.

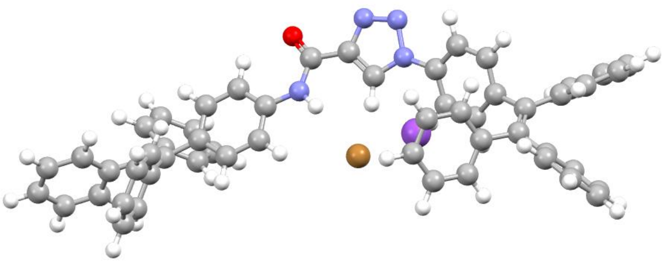

Figure S 23 DFT optimized (B3LYP/3-21g) structure of **1-Br$^-$** system, arrangement 1 (in the form of sodium salt).

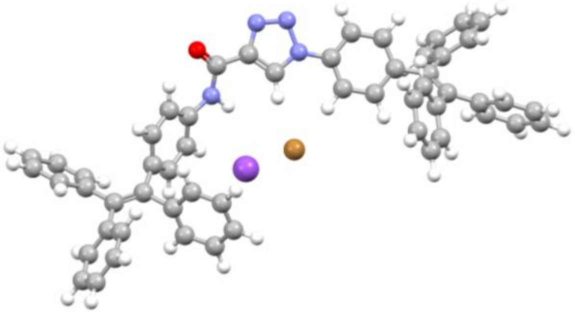

Figure S 24 DFT optimized (B3LYP/3-21g) structure of **1-Br**$^{-}$ system, arrangement 2 (in the form of sodium salt).

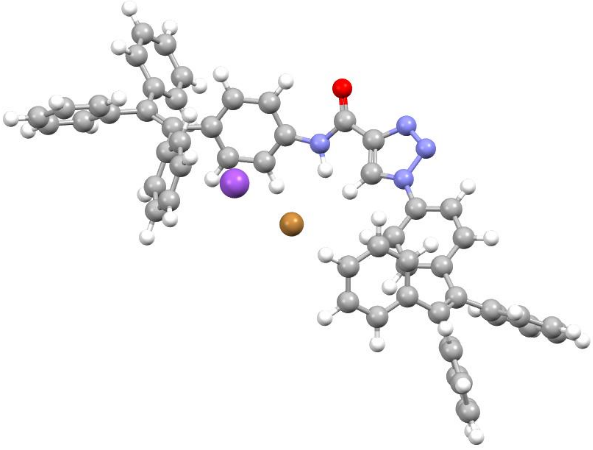

Figure S 25 DFT optimized (B3LYP/3-21g) structure of **1-Br**$^{-}$ system, arrangement 3 (in the form of sodium salt).

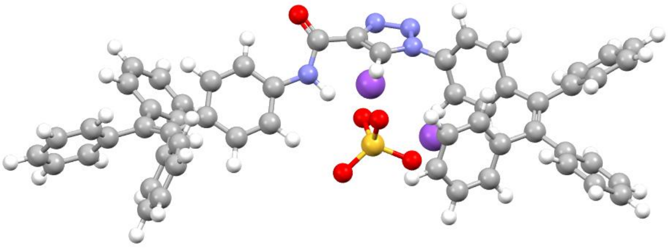

Figure S 26 DFT optimized (B3LYP/3-21g) structure of **1-SO$_4$$^{2-}$** system (in the form of sodium salt).

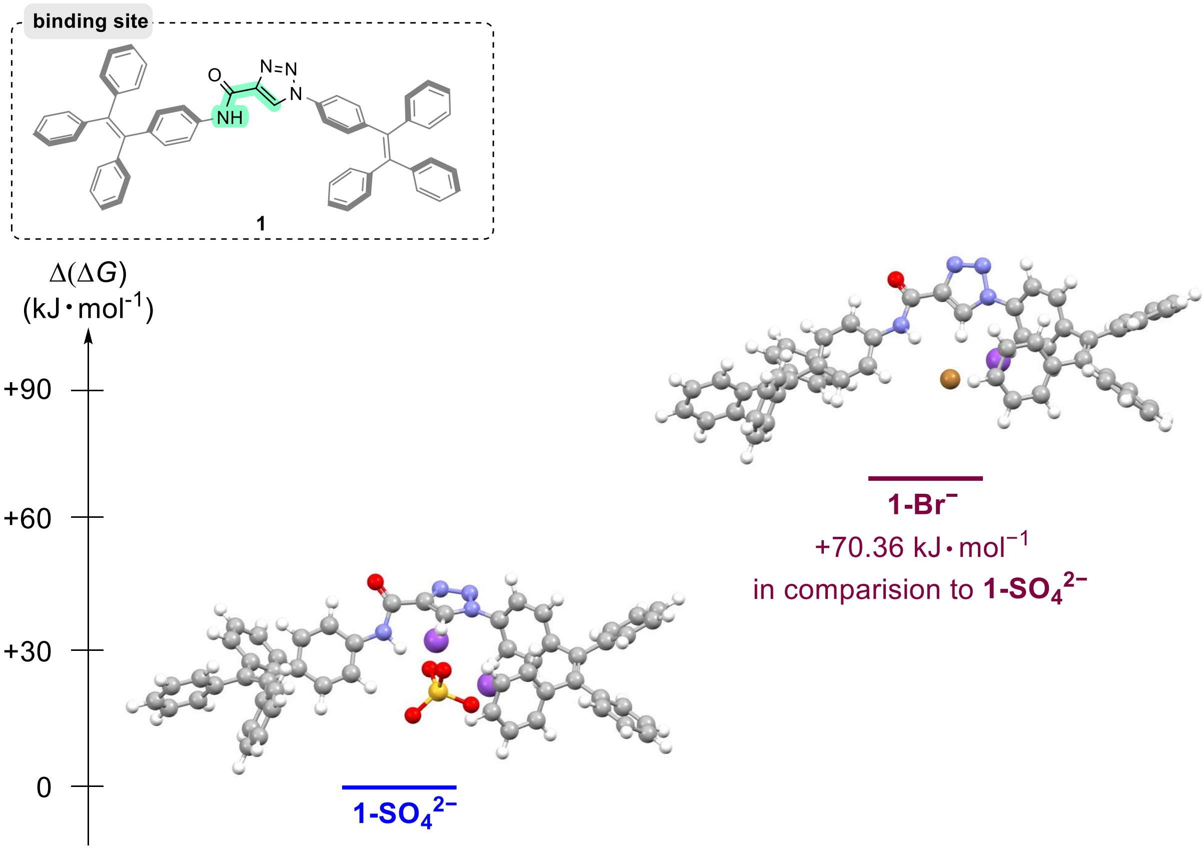

Figure S 27 Graphical presentation of the results of DFT studies (B3LYP/3-21g) regarding comparison of Δ*G* value for system **1-**$\mathbf{SO_4^{2-}}$ and **1-**$\mathbf{Br^-}$ (in the form of sodium salts; arrangement 1 for both cases). Reference Δ*G* value was taken for the system featuring the lower Δ*G* value (**1-**$\mathbf{SO_4^{2-}}$).

Table S 5 Atomic coordinates for the DFT optimized (B3LYP/6-31g(d,p)) structure of **1**.

	x	*y*	*z*
C	-12.0475000000	0.6392000000	0.8808000000
C	-12.3577000000	1.5106000000	-0.1652000000
C	-11.3619000000	1.8775000000	-1.0721000000
C	-10.0651000000	1.3873000000	-0.9283000000
C	-9.7346000000	0.5181000000	0.1251000000
C	-10.7537000000	0.1398000000	1.0171000000
C	-8.3565000000	-0.0436000000	0.2666000000
C	-7.2340000000	0.7350000000	0.2186000000
C	-8.3039000000	-1.5244000000	0.4651000000
C	-5.8597000000	0.1667000000	0.0919000000
C	-7.2876000000	2.2280000000	0.2917000000
C	-7.5550000000	-2.0941000000	1.5087000000
C	-7.5391000000	-3.4733000000	1.7082000000
C	-8.2707000000	-4.3143000000	0.8679000000
C	-9.0286000000	-3.7625000000	-0.1666000000
C	-9.0541000000	-2.3823000000	-0.3583000000
C	-4.8092000000	0.6412000000	0.8968000000
C	-3.5220000000	0.1362000000	0.7708000000
C	-3.2302000000	-0.8513000000	-0.1837000000
C	-4.2600000000	-1.3235000000	-1.0120000000
C	-5.5467000000	-0.8135000000	-0.8642000000
C	-6.5661000000	3.0146000000	-0.6235000000
C	-6.5909000000	4.4061000000	-0.5483000000
C	-7.3194000000	5.0425000000	0.4587000000
C	-8.0229000000	4.2743000000	1.3880000000
C	-8.0076000000	2.8829000000	1.3050000000
N	-1.8968000000	-1.2995000000	-0.2657000000
C	-1.3622000000	-2.2838000000	-1.0762000000
O	-2.0049000000	-2.9687000000	-1.8553000000
C	0.1161000000	-2.4240000000	-0.9269000000
C	1.0786000000	-1.4791000000	-0.6271000000
N	2.2541000000	-2.1567000000	-0.6833000000
N	2.0233000000	-3.4659000000	-1.0019000000
N	0.7459000000	-3.6183000000	-1.1547000000
C	3.5787000000	-1.6795000000	-0.4816000000
C	3.8124000000	-0.5822000000	0.3515000000
C	5.1101000000	-0.1117000000	0.5221000000
C	6.2001000000	-0.7283000000	-0.1150000000
C	5.9392000000	-1.8554000000	-0.9154000000
C	4.6453000000	-2.3224000000	-1.1164000000
C	7.5993000000	-0.2518000000	0.0978000000
C	7.9471000000	1.0672000000	0.0114000000

C	8.5862000000	-1.3319000000	0.4056000000
C	9.2705000000	1.5848000000	0.4736000000
C	7.0341000000	2.1052000000	-0.5579000000
C	6.4161000000	1.9261000000	-1.8071000000
C	5.5927000000	2.9132000000	-2.3463000000
C	5.3678000000	4.1003000000	-1.6472000000
C	5.9839000000	4.2977000000	-0.4097000000
C	6.8176000000	3.3162000000	0.1231000000
C	9.7789000000	1.2584000000	1.7423000000
C	10.9965000000	1.7765000000	2.1795000000
C	11.7339000000	2.6302000000	1.3572000000
C	11.2373000000	2.9712000000	0.0977000000
C	10.0134000000	2.4636000000	-0.3343000000
C	8.2990000000	-2.3076000000	1.3763000000
C	9.2028000000	-3.3323000000	1.6510000000
C	10.4046000000	-3.4161000000	0.9454000000
C	10.6941000000	-2.4664000000	-0.0359000000
C	9.7952000000	-1.4353000000	-0.3028000000
H	-12.8159000000	0.3432000000	1.5894000000
H	-13.3678000000	1.8939000000	-0.2769000000
H	-11.5954000000	2.5439000000	-1.8976000000
H	-9.2968000000	1.6750000000	-1.6382000000
H	-10.5228000000	-0.5484000000	1.8247000000
H	-6.9843000000	-1.4457000000	2.1653000000
H	-6.9557000000	-3.8916000000	2.5235000000
H	-8.2547000000	-5.3894000000	1.0211000000
H	-9.6042000000	-4.4077000000	-0.8243000000
H	-9.6553000000	-1.9590000000	-1.1572000000
H	-5.0070000000	1.4128000000	1.6342000000
H	-2.7311000000	0.5145000000	1.4154000000
H	-4.0436000000	-2.0797000000	-1.7527000000
H	-6.3316000000	-1.1879000000	-1.5125000000
H	-5.9882000000	2.5257000000	-1.4020000000
H	-6.0375000000	4.9942000000	-1.2751000000
H	-7.3335000000	6.1267000000	0.5222000000
H	-8.5833000000	4.7586000000	2.1826000000
H	-8.5570000000	2.2917000000	2.0301000000
H	-1.2737000000	-0.9136000000	0.4290000000
H	1.0300000000	-0.4160000000	-0.4545000000
H	2.9929000000	-0.1126000000	0.8856000000
H	5.2847000000	0.7396000000	1.1703000000
H	6.7655000000	-2.3646000000	-1.4005000000
H	4.4496000000	-3.1775000000	-1.7517000000
H	6.5906000000	1.0074000000	-2.3576000000
H	5.1314000000	2.7567000000	-3.3172000000

H	4.7258000000	4.8691000000	-2.0673000000
H	5.8206000000	5.2208000000	0.1392000000
H	7.3058000000	3.4821000000	1.0787000000
H	9.2111000000	0.5956000000	2.3868000000
H	11.3677000000	1.5153000000	3.1663000000
H	12.6837000000	3.0319000000	1.6976000000
H	11.8013000000	3.6380000000	-0.5483000000
H	9.6270000000	2.7435000000	-1.3096000000
H	7.3613000000	-2.2560000000	1.9217000000
H	8.9658000000	-4.0688000000	2.4134000000
H	11.1058000000	-4.2188000000	1.1534000000
H	11.6205000000	-2.5301000000	-0.5993000000
H	10.0258000000	-0.7009000000	-1.0674000000

Table S 6 Atomic coordinates for the DFT optimized (B3LYP/6-31g(d,p)) structure of **2**.

	x	*y*	*z*
C	12.2415000000	0.7737000000	-0.7013000000
C	12.5722000000	1.3659000000	0.5191000000
C	11.5893000000	1.5156000000	1.4990000000
C	10.2850000000	1.0878000000	1.2571000000
C	9.9339000000	0.4997000000	0.0302000000
C	10.9400000000	0.3352000000	-0.9382000000
C	8.5474000000	0.0027000000	-0.2252000000
C	7.4358000000	0.7594000000	0.0206000000
C	8.4735000000	-1.3871000000	-0.7708000000
C	6.0553000000	0.1917000000	0.0248000000
C	7.5083000000	2.2259000000	0.3053000000
C	7.7075000000	-1.6832000000	-1.9109000000
C	7.6716000000	-2.9746000000	-2.4336000000
C	8.3999000000	-3.9996000000	-1.8273000000
C	9.1747000000	-3.7188000000	-0.7004000000
C	9.2200000000	-2.4247000000	-0.1852000000
C	5.0036000000	0.8557000000	-0.6307000000
C	3.7109000000	0.3494000000	-0.6122000000
C	3.4153000000	-0.8338000000	0.0834000000
C	4.4466000000	-1.5013000000	0.7618000000
C	5.7385000000	-0.9847000000	0.7235000000
C	6.8041000000	2.7791000000	1.3894000000
C	6.8461000000	4.1479000000	1.6481000000
C	7.5749000000	4.9985000000	0.8143000000
C	8.2613000000	4.4668000000	-0.2788000000
C	8.2287000000	3.0962000000	-0.5300000000
N	2.0762000000	-1.2735000000	0.0745000000
C	1.5371000000	-2.4171000000	0.6329000000
O	2.1779000000	-3.2769000000	1.2152000000
C	0.0546000000	-2.5015000000	0.4773000000
C	-0.8994000000	-1.5033000000	0.4419000000
N	-2.0821000000	-2.1621000000	0.3346000000
N	-1.8633000000	-3.5113000000	0.3010000000
N	-0.5865000000	-3.7086000000	0.3932000000
C	-3.4037000000	-1.6400000000	0.2702000000
C	-3.7073000000	-0.4118000000	0.8628000000
C	-5.0020000000	0.0908000000	0.7782000000
C	-6.0199000000	-0.6186000000	0.1200000000
C	-5.6887000000	-1.8578000000	-0.4551000000
C	-4.3972000000	-2.3685000000	-0.3895000000
C	-7.4008000000	-0.0781000000	0.0411000000
C	-8.5082000000	-0.9317000000	0.1428000000

C	-9.8182000000	-0.4406000000	0.0543000000
C	-10.0055000000	0.9354000000	-0.1399000000
C	-8.9193000000	1.8157000000	-0.2473000000
C	-7.6219000000	1.2926000000	-0.1545000000
C	-9.1382000000	3.2710000000	-0.4550000000
C	-10.9823000000	-1.3561000000	0.1822000000
C	-12.1257000000	-1.1819000000	-0.6150000000
C	-13.2176000000	-2.0403000000	-0.4980000000
C	-13.1894000000	-3.0923000000	0.4189000000
C	-12.0601000000	-3.2777000000	1.2179000000
C	-10.9687000000	-2.4186000000	1.1012000000
C	-8.3247000000	4.2218000000	0.1838000000
C	-8.5291000000	5.5866000000	-0.0117000000
C	-9.5530000000	6.0309000000	-0.8497000000
C	-10.3700000000	5.0982000000	-1.4905000000
C	-10.1644000000	3.7335000000	-1.2956000000
H	12.9996000000	0.6478000000	-1.4691000000
H	13.5882000000	1.7005000000	0.7075000000
H	11.8387000000	1.9624000000	2.4572000000
H	9.5267000000	1.2054000000	2.0241000000
H	10.6931000000	-0.1371000000	-1.8842000000
H	7.1393000000	-0.8908000000	-2.3870000000
H	7.0753000000	-3.1799000000	-3.3181000000
H	8.3684000000	-5.0067000000	-2.2325000000
H	9.7479000000	-4.5084000000	-0.2228000000
H	9.8344000000	-2.2107000000	0.6842000000
H	5.2048000000	1.7786000000	-1.1656000000
H	2.9190000000	0.8789000000	-1.1381000000
H	4.2273000000	-2.4101000000	1.3034000000
H	6.5247000000	-1.5115000000	1.2537000000
H	6.2261000000	2.1246000000	2.0349000000
H	6.3061000000	4.5514000000	2.5001000000
H	7.6025000000	6.0663000000	1.0112000000
H	8.8216000000	5.1209000000	-0.9408000000
H	8.7648000000	2.6894000000	-1.3811000000
H	1.4517000000	-0.7263000000	-0.5004000000
H	-0.8352000000	-0.4284000000	0.4907000000
H	-2.9504000000	0.1343000000	1.4162000000
H	-5.2348000000	1.0325000000	1.2646000000
H	-6.4478000000	-2.4175000000	-0.9918000000
H	-4.1462000000	-3.3178000000	-0.8466000000
H	-8.3499000000	-1.9944000000	0.2954000000
H	-11.0143000000	1.3349000000	-0.1578000000
H	-6.7731000000	1.9552000000	-0.2893000000
H	-12.1472000000	-0.3823000000	-1.3494000000

H	-14.0882000000	-1.8920000000	-1.1303000000
H	-14.0397000000	-3.7615000000	0.5100000000
H	-12.0313000000	-4.0877000000	1.9409000000
H	-10.1065000000	-2.5553000000	1.7471000000
H	-7.5432000000	3.8869000000	0.8593000000
H	-7.8935000000	6.3041000000	0.4993000000
H	-9.7130000000	7.0942000000	-1.0016000000
H	-11.1645000000	5.4331000000	-2.1511000000
H	-10.7887000000	3.0158000000	-1.8193000000

Table S 7 Atomic coordinates for the DFT optimized (B3LYP/3-21g) structure of **1-Br**⁻ system, arrangement 1 (in the form of sodium salt).

	x	*y*	*z*
C	11.6085000000	0.7493000000	-1.6693000000
C	12.1839000000	1.2627000000	-0.5037000000
C	11.4247000000	1.3339000000	0.6673000000
C	10.0977000000	0.9051000000	0.6725000000
C	9.5056000000	0.3954000000	-0.4956000000
C	10.2847000000	0.3107000000	-1.6634000000
C	8.0942000000	-0.1018000000	-0.4976000000
C	7.0606000000	0.6093000000	0.0362000000
C	7.9114000000	-1.4436000000	-1.1342000000
C	5.7026000000	0.0296000000	0.2548000000
C	7.2010000000	2.0400000000	0.4539000000
C	6.8955000000	-1.6691000000	-2.0787000000
C	6.7597000000	-2.9141000000	-2.6913000000
C	7.6339000000	-3.9565000000	-2.3711000000
C	8.6526000000	-3.7417000000	-1.4394000000
C	8.7965000000	-2.4939000000	-0.8330000000
C	4.5526000000	0.7580000000	-0.1017000000
C	3.2824000000	0.2409000000	0.1126000000
C	3.1160000000	-1.0201000000	0.7157000000
C	4.2571000000	-1.7472000000	1.0978000000
C	5.5242000000	-1.2241000000	0.8621000000
C	6.6919000000	2.4638000000	1.6943000000
C	6.7950000000	3.7981000000	2.0869000000
C	7.3896000000	4.7361000000	1.2388000000
C	7.8809000000	4.3295000000	-0.0047000000
C	7.7889000000	2.9935000000	-0.3942000000
N	1.7973000000	-1.4828000000	0.9068000000
C	1.4092000000	-2.6840000000	1.4748000000
O	2.1854000000	-3.5165000000	1.9692000000
C	-0.0598000000	-2.8824000000	1.4416000000
C	-0.9956000000	-2.1998000000	0.6975000000
N	-2.2077000000	-2.7119000000	1.0510000000
N	-2.0150000000	-3.7624000000	2.0433000000
N	-0.7066000000	-3.8301000000	2.2304000000
C	-3.4885000000	-2.2587000000	0.6837000000
C	-3.6705000000	-1.5444000000	-0.5118000000
C	-4.9069000000	-0.9515000000	-0.7820000000
C	-5.9803000000	-1.0833000000	0.1146000000
C	-5.8142000000	-1.9022000000	1.2441000000
C	-4.5789000000	-2.4704000000	1.5396000000
C	-7.2655000000	-0.3646000000	-0.1509000000
C	-7.2821000000	0.9958000000	-0.2081000000

C	-8.4592000000	-1.2374000000	-0.3366000000
C	-8.4288000000	1.7989000000	-0.7267000000
C	-6.1212000000	1.8159000000	0.2757000000
C	-5.6746000000	1.7059000000	1.6085000000
C	-4.6394000000	2.5220000000	2.0868000000
C	-4.0290000000	3.4571000000	1.2374000000
C	-4.4654000000	3.5717000000	-0.0914000000
C	-5.5076000000	2.7658000000	-0.5645000000
C	-9.0598000000	1.4539000000	-1.9349000000
C	-10.1029000000	2.2314000000	-2.4347000000
C	-10.5346000000	3.3633000000	-1.7371000000
C	-9.9104000000	3.7188000000	-0.5389000000
C	-8.8595000000	2.9486000000	-0.0409000000
C	-8.3403000000	-2.4535000000	-1.0337000000
C	-9.4405000000	-3.2979000000	-1.1805000000
C	-10.6708000000	-2.9512000000	-0.6176000000
C	-10.7953000000	-1.7537000000	0.0932000000
C	-9.7014000000	-0.9024000000	0.2326000000
H	12.1923000000	0.6868000000	-2.5808000000
H	13.2150000000	1.5971000000	-0.5062000000
H	11.8676000000	1.7204000000	1.5783000000
H	9.5085000000	0.9657000000	1.5785000000
H	9.8427000000	-0.1041000000	-2.5614000000
H	6.2117000000	-0.8652000000	-2.3195000000
H	5.9716000000	-3.0713000000	-3.4189000000
H	7.5240000000	-4.9251000000	-2.8453000000
H	9.3355000000	-4.5450000000	-1.1867000000
H	9.5954000000	-2.3216000000	-0.1218000000
H	4.6634000000	1.7370000000	-0.5522000000
H	2.4056000000	0.8078000000	-0.1838000000
H	4.1233000000	-2.7023000000	1.5783000000
H	6.3949000000	-1.7969000000	1.1551000000
H	6.2137000000	1.7382000000	2.3412000000
H	6.4071000000	4.1068000000	3.0511000000
H	7.4632000000	5.7747000000	1.5407000000
H	8.3330000000	5.0538000000	-0.6728000000
H	8.1750000000	2.6778000000	-1.3549000000
H	1.0745000000	-0.8194000000	0.6183000000
H	-0.8902000000	-1.3502000000	0.0378000000
H	-2.8530000000	-1.4416000000	-1.2161000000
H	-5.0441000000	-0.3894000000	-1.7007000000
H	-6.6532000000	-2.0619000000	1.9110000000
H	-4.4266000000	-3.0606000000	2.4329000000
H	-6.1546000000	0.9947000000	2.2694000000
H	-4.3225000000	2.4405000000	3.1205000000

H	-3.2270000000	4.0864000000	1.6032000000
H	-3.9943000000	4.2884000000	-0.7532000000
H	-5.8610000000	2.8764000000	-1.5829000000
H	-8.7292000000	0.5744000000	-2.4728000000
H	-10.5764000000	1.9573000000	-3.3701000000
H	-11.3460000000	3.9661000000	-2.1275000000
H	-10.2389000000	4.5967000000	0.0049000000
H	-8.3693000000	3.2328000000	0.8830000000
H	-7.3831000000	-2.7319000000	-1.4583000000
H	-9.3358000000	-4.2272000000	-1.7280000000
H	-11.5234000000	-3.6115000000	-0.7244000000
H	-11.7442000000	-1.4864000000	0.5434000000
H	-9.8008000000	0.0250000000	0.7817000000
Na	-3.2986000000	0.9887000000	0.2640000000
Br	-0.7322000000	1.0583000000	-0.4856000000

Table S 8 Atomic coordinates for the DFT optimized (B3LYP/3-21g) structure of **1-Br**$^{-}$ system, arrangement 2 (in the form of sodium salt).

	x	*y*	*z*
C	-10.5128000000	3.0106000000	0.9865000000
C	-10.3451000000	3.9081000000	-0.0722000000
C	-9.3789000000	3.6549000000	-1.0479000000
C	-8.5745000000	2.5171000000	-0.9613000000
C	-8.7285000000	1.6155000000	0.1029000000
C	-9.7181000000	1.8684000000	1.0690000000
C	-7.8811000000	0.3868000000	0.2277000000
C	-6.5339000000	0.4451000000	0.1679000000
C	-8.6280000000	-0.8943000000	0.4175000000
C	-5.5733000000	-0.7081000000	0.2203000000
C	-5.7272000000	1.7129000000	0.0364000000
C	-8.2215000000	-1.8675000000	1.3451000000
C	-8.9445000000	-3.0518000000	1.4916000000
C	-10.0805000000	-3.2845000000	0.7134000000
C	-10.4981000000	-2.3198000000	-0.2073000000
C	-9.7843000000	-1.1310000000	-0.3482000000
C	-4.7134000000	-0.8242000000	1.3300000000
C	-3.5566000000	-1.5961000000	1.2604000000
C	-3.2087000000	-2.2577000000	0.0615000000
C	-4.1467000000	-2.2748000000	-0.9901000000
C	-5.3018000000	-1.4971000000	-0.9079000000
C	-5.0086000000	1.9568000000	-1.1537000000
C	-4.0803000000	3.0028000000	-1.2270000000
C	-3.8390000000	3.8082000000	-0.1040000000
C	-4.5579000000	3.5809000000	1.0754000000
C	-5.4991000000	2.5461000000	1.1444000000
N	-1.9106000000	-2.7631000000	-0.0492000000
C	-1.3841000000	-3.4717000000	-1.1345000000
O	-2.0939000000	-4.0205000000	-1.9889000000
C	0.0859000000	-3.4352000000	-1.1575000000
C	0.9076000000	-2.4525000000	-0.6296000000
N	2.1720000000	-2.8006000000	-0.9742000000
N	2.1384000000	-4.0341000000	-1.7398000000
N	0.8580000000	-4.3677000000	-1.8323000000
C	3.4027000000	-2.1421000000	-0.7141000000
C	3.4275000000	-0.9033000000	-0.0616000000
C	4.6462000000	-0.2773000000	0.1803000000
C	5.8581000000	-0.8677000000	-0.2153000000
C	5.8112000000	-2.1207000000	-0.8525000000
C	4.5995000000	-2.7496000000	-1.1107000000
C	7.1734000000	-0.2225000000	0.0781000000
C	7.4186000000	1.0956000000	-0.1646000000

C	8.2003000000	-1.1446000000	0.6570000000
C	8.6414000000	1.7981000000	0.3342000000
C	6.4860000000	1.9573000000	-0.9558000000
C	5.9935000000	1.5395000000	-2.2035000000
C	5.1494000000	2.3655000000	-2.9453000000
C	4.7817000000	3.6203000000	-2.4527000000
C	5.2728000000	4.0495000000	-1.2167000000
C	6.1261000000	3.2299000000	-0.4786000000
C	9.0587000000	1.6751000000	1.6703000000
C	10.1801000000	2.3652000000	2.1294000000
C	10.9055000000	3.1870000000	1.2625000000
C	10.4951000000	3.3228000000	-0.0663000000
C	9.3671000000	2.6426000000	-0.5246000000
C	7.8571000000	-2.0086000000	1.7118000000
C	8.7983000000	-2.8903000000	2.2428000000
C	10.0920000000	-2.9370000000	1.7165000000
C	10.4380000000	-2.0951000000	0.6562000000
C	9.5016000000	-1.2052000000	0.1308000000
H	-11.2652000000	3.1989000000	1.7435000000
H	-10.9681000000	4.7923000000	-0.1397000000
H	-9.2547000000	4.3388000000	-1.8795000000
H	-7.8324000000	2.3204000000	-1.7244000000
H	-9.8580000000	1.1621000000	1.8786000000
H	-7.3434000000	-1.6947000000	1.9525000000
H	-8.6214000000	-3.7910000000	2.2153000000
H	-10.6383000000	-4.2067000000	0.8267000000
H	-11.3803000000	-2.4926000000	-0.8127000000
H	-10.1147000000	-0.3769000000	-1.0519000000
H	-4.9306000000	-0.2714000000	2.2398000000
H	-2.8790000000	-1.6339000000	2.1050000000
H	-3.9245000000	-2.8677000000	-1.8637000000
H	-5.9829000000	-1.4678000000	-1.7510000000
H	-5.1737000000	1.3163000000	-2.0135000000
H	-3.5439000000	3.1853000000	-2.1516000000
H	-3.1043000000	4.6026000000	-0.1513000000
H	-4.3811000000	4.2047000000	1.9439000000
H	-6.0522000000	2.3721000000	2.0598000000
H	-1.2555000000	-2.4030000000	0.6451000000
H	0.6478000000	-1.5479000000	-0.0958000000
H	2.5098000000	-0.4236000000	0.2592000000
H	4.6598000000	0.6828000000	0.6788000000
H	6.7370000000	-2.6014000000	-1.1433000000
H	4.5580000000	-3.7059000000	-1.6117000000
H	6.2736000000	0.5649000000	-2.5826000000
H	4.7802000000	2.0306000000	-3.9078000000

H	4.1227000000	4.2596000000	-3.0287000000
H	4.9932000000	5.0227000000	-0.8297000000
H	6.5210000000	3.5675000000	0.4722000000
H	8.5010000000	1.0341000000	2.3409000000
H	10.4863000000	2.2640000000	3.1643000000
H	11.7779000000	3.7212000000	1.6208000000
H	11.0506000000	3.9616000000	-0.7435000000
H	9.0371000000	2.7609000000	-1.5497000000
H	6.8489000000	-1.9834000000	2.1077000000
H	8.5216000000	-3.5435000000	3.0625000000
H	10.8213000000	-3.6276000000	2.1241000000
H	11.4365000000	-2.1340000000	0.2361000000
H	9.7716000000	-0.5488000000	-0.6866000000
Na	-2.0164000000	1.0889000000	0.1073000000
Br	0.5132000000	0.5891000000	0.8485000000

Table S 9 Atomic coordinates for the DFT optimized (B3LYP/3-21g) structure of **1-Br**⁻ system, arrangement 3 (in the form of sodium salt).

	x	*y*	*z*
C	-10.5128190000	3.0105900000	0.9862740000
C	-10.3451030000	3.9080300000	-0.0724850000
C	-9.3787540000	3.6548520000	-1.0479730000
C	-8.5742500000	2.5170830000	-0.9612690000
C	-8.7283070000	1.6155760000	0.1029840000
C	-9.7180920000	1.8684270000	1.0689000000
C	-7.8808600000	0.3869120000	0.2278880000
C	-6.5336440000	0.4451670000	0.1677430000
C	-8.6277740000	-0.8941750000	0.4182690000
C	-5.5731880000	-0.7081420000	0.2202140000
C	-5.7269430000	1.7128030000	0.0357630000
C	-8.2212800000	-1.8668710000	1.3464770000
C	-8.9441500000	-3.0510720000	1.4935830000
C	-10.0801360000	-3.2842790000	0.7153320000
C	-10.4977100000	-2.3200860000	-0.2059240000
C	-9.7839080000	-1.1313810000	-0.3473980000
C	-4.7130830000	-0.8239050000	1.3298250000
C	-3.5562450000	-1.5958550000	1.2601930000
C	-3.2085950000	-2.2577040000	0.0614590000
C	-4.1468250000	-2.2751760000	-0.9899520000
C	-5.3019190000	-1.4974590000	-0.9077760000
C	-5.0083860000	1.9563190000	-1.1544500000
C	-4.0800110000	3.0022950000	-1.2281220000
C	-3.8386960000	3.8080240000	-0.1053780000
C	-4.5576240000	3.5811690000	1.0741300000
C	-5.4987770000	2.5463850000	1.1434690000
N	-1.9104260000	-2.7630010000	-0.0494600000
C	-1.3842280000	-3.4720100000	-1.1345970000
O	-2.0942300000	-4.0211580000	-1.9886850000
C	0.0857710000	-3.4355010000	-1.1579510000
C	0.9074870000	-2.4526470000	-0.6302390000
N	2.1718420000	-2.8008110000	-0.9749270000
N	2.1383280000	-4.0341960000	-1.7404180000
N	0.8578850000	-4.3680030000	-1.8327330000
C	3.4025450000	-2.1421920000	-0.7147790000
C	3.4271810000	-0.9034310000	-0.0621650000
C	4.6458260000	-0.2773630000	0.1798960000
C	5.8578150000	-0.8677070000	-0.2155820000
C	5.8110570000	-2.1206170000	-0.8529270000
C	4.5994340000	-2.7495810000	-1.1113400000
C	7.1730270000	-0.2224780000	0.0781410000
C	7.4182720000	1.0956690000	-0.1642400000

C	8.1999170000	-1.1446610000	0.6569010000
C	8.6409420000	1.7980530000	0.3347840000
C	6.4857330000	1.9575580000	-0.9553870000
C	5.9934930000	1.5401090000	-2.2033560000
C	5.1495890000	2.3662320000	-2.9450870000
C	4.7816360000	3.6209120000	-2.4522090000
C	5.2724840000	4.0498110000	-1.2160740000
C	6.1257150000	3.2300450000	-0.4779970000
C	9.0581630000	1.6747530000	1.6709640000
C	10.1795050000	2.3647400000	2.1303030000
C	10.9048880000	3.1868310000	1.2637110000
C	10.4946270000	3.3229750000	-0.0651540000
C	9.3667360000	2.6428860000	-0.5237030000
C	7.8566780000	-2.0088800000	1.7115250000
C	8.7978920000	-2.8906200000	2.2424200000
C	10.0915700000	-2.9372620000	1.7161450000
C	10.4375970000	-2.0951710000	0.6559590000
C	9.5011520000	-1.2052590000	0.1306320000
H	-11.2654090000	3.1988650000	1.7431640000
H	-10.9681280000	4.7921730000	-0.1401080000
H	-9.2544650000	4.3387420000	-1.8795940000
H	-7.8321110000	2.3203630000	-1.7243130000
H	-9.8580950000	1.1621730000	1.8785220000
H	-7.3432510000	-1.6936650000	1.9538230000
H	-8.6210880000	-3.7898960000	2.2176450000
H	-10.6378350000	-4.2064600000	0.8290560000
H	-11.3798350000	-2.4933360000	-0.8113050000
H	-10.1143300000	-0.3776250000	-1.0515750000
H	-4.9301210000	-0.2707890000	2.2395070000
H	-2.8784250000	-1.6333950000	2.1046530000
H	-3.9247950000	-2.8683440000	-1.8634110000
H	-5.9832410000	-1.4684240000	-1.7506760000
H	-5.1735580000	1.3155870000	-2.0139940000
H	-3.5436020000	3.1844490000	-2.1527150000
H	-3.1040400000	4.6024540000	-0.1529980000
H	-4.3807950000	4.2052160000	1.9423590000
H	-6.0519170000	2.3726920000	2.0589290000
H	-1.2551870000	-2.4025990000	0.6445250000
H	0.6477220000	-1.5481990000	-0.0962580000
H	2.5092880000	-0.4237920000	0.2584300000
H	4.6592280000	0.6826360000	0.6784910000
H	6.7368640000	-2.6012390000	-1.1436690000
H	4.5579970000	-3.7058840000	-1.6123630000
H	6.2737760000	0.5656060000	-2.5826170000
H	4.7805810000	2.0315570000	-3.9077930000

H	4.1227370000	4.2603380000	-3.0282030000
H	4.9927040000	5.0228880000	-0.8287950000
H	6.5203410000	3.5673860000	0.4729710000
H	8.5004050000	1.0335510000	2.3413640000
H	10.4855740000	2.2633300000	3.1652490000
H	11.7772740000	3.7210130000	1.6221560000
H	11.0501740000	3.9619760000	-0.7421630000
H	9.0367720000	2.7614410000	-1.5488230000
H	6.8484180000	-1.9837170000	2.1074950000
H	8.5211700000	-3.5439100000	3.0620540000
H	10.8208290000	-3.6278820000	2.1236240000
H	11.4361280000	-2.1340700000	0.2358590000
H	9.7711490000	-0.5486750000	-0.6866290000
Na	-2.9914700000	0.9124810000	0.3274540000
Br	-0.4618530000	0.4130060000	1.0687470000

Table S 10 Atomic coordinates for the DFT optimized (B3LYP/3-21g) structure of **1-SO$_4^{2-}$** system (in the form of sodium salt).

	x	*y*	*z*
C	11.7007890000	1.1322610000	-0.9593630000
C	12.0754930000	1.8039730000	0.2074020000
C	11.1621230000	1.9244470000	1.2581850000
C	9.8806960000	1.3866600000	1.1414650000
C	9.4896580000	0.7165840000	-0.0302220000
C	10.4230370000	0.5851720000	-1.0739540000
C	8.1309350000	0.1025420000	-0.1563180000
C	6.9856040000	0.7691860000	0.1633850000
C	8.1318640000	-1.3051390000	-0.6641310000
C	5.6596860000	0.0907930000	0.2702210000
C	6.9580520000	2.2396970000	0.4390920000
C	7.2718220000	-1.7049920000	-1.7013800000
C	7.3109140000	-3.0094230000	-2.1924900000
C	8.2064560000	-3.9387130000	-1.6558200000
C	9.0711770000	-3.5504600000	-0.6293970000
C	9.0413400000	-2.2430480000	-0.1442840000
C	4.5189250000	0.6568890000	-0.3288370000
C	3.2745850000	0.0471410000	-0.2294940000
C	3.1363290000	-1.1507040000	0.4995070000
C	4.2610640000	-1.7123730000	1.1265380000
C	5.5018370000	-1.0948650000	1.0053650000
C	6.2430130000	2.7341700000	1.5442230000
C	6.1890630000	4.1038500000	1.8010740000
C	6.8310600000	5.0045920000	0.9472080000
C	7.5276330000	4.5252220000	-0.1654150000
C	7.5922700000	3.1552960000	-0.4175650000
N	1.8465990000	-1.7271120000	0.5633640000
C	1.4627930000	-2.8391360000	1.2779560000
O	2.2019300000	-3.5926850000	1.9330140000
C	-0.0087930000	-3.0695670000	1.1035060000
C	-1.0298650000	-2.1365990000	1.1854480000
N	-2.1595210000	-2.7900380000	0.8050540000
N	-1.8604030000	-4.1505260000	0.4348170000
N	-0.5231290000	-4.2767320000	0.6499680000
C	-3.4671680000	-2.2304850000	0.6145540000
C	-3.7339000000	-1.5203930000	-0.5580050000
C	-4.9670340000	-0.8789770000	-0.6998850000
C	-5.9348780000	-0.9437520000	0.3158030000
C	-5.6754170000	-1.7323040000	1.4493650000
C	-4.4432250000	-2.3660660000	1.6042300000

C	-7.2209910000	-0.1922680000	0.1702400000
C	-7.2175950000	1.1564820000	-0.0119850000
C	-8.4532840000	-1.0303700000	0.2394440000
C	-8.4181070000	1.9448120000	-0.4207850000
C	-5.9835670000	1.9843900000	0.2048670000
C	-5.3519700000	2.0094250000	1.4647260000
C	-4.2433300000	2.8364810000	1.6915940000
C	-3.7425290000	3.6434160000	0.6591000000
C	-4.3625400000	3.6210680000	-0.5995290000
C	-5.4792670000	2.8063400000	-0.8215440000
C	-9.2410400000	1.5091230000	-1.4742280000
C	-10.3343350000	2.2728300000	-1.8791640000
C	-10.6260260000	3.4804620000	-1.2385280000
C	-9.8110900000	3.9257080000	-0.1947760000
C	-8.7098110000	3.1694820000	0.2057430000
C	-8.4907560000	-2.2825490000	-0.3998330000
C	-9.6244060000	-3.0901630000	-0.3116330000
C	-10.7299180000	-2.6688550000	0.4310710000
C	-10.6959850000	-1.4335830000	1.0847740000
C	-9.5691240000	-0.6192870000	0.9900810000
H	12.4049740000	1.0313370000	-1.7776060000
H	13.0707990000	2.2234780000	0.2993660000
H	11.4491340000	2.4347820000	2.1706140000
H	9.1716060000	1.4854290000	1.9534120000
H	10.1368420000	0.0491470000	-1.9709890000
H	6.5719900000	-0.9881340000	-2.1114190000
H	6.6445350000	-3.3002150000	-2.9968430000
H	8.2334640000	-4.9531300000	-2.0369650000
H	9.7702170000	-4.2647340000	-0.2091180000
H	9.7228420000	-1.9364490000	0.6402100000
H	4.6130110000	1.5877970000	-0.8749410000
H	2.4075520000	0.5066750000	-0.6984530000
H	4.1375700000	-2.6191570000	1.6971330000
H	6.3654750000	-1.5388270000	1.4842120000
H	5.7283030000	2.0353250000	2.1924880000
H	5.6407750000	4.4685600000	2.6623230000
H	6.7818700000	6.0698700000	1.1418210000
H	8.0170240000	5.2190730000	-0.8394790000
H	8.1358380000	2.7842230000	-1.2769270000
H	1.1266220000	-1.2885250000	-0.0566400000
H	-1.0026510000	-1.0539780000	1.2989160000
H	-2.9670960000	-1.3567500000	-1.3093010000
H	-5.1703220000	-0.3112370000	-1.6027260000
H	-6.4345860000	-1.8270190000	2.2161930000
H	-4.2246390000	-2.9445000000	2.4928450000

H	-5.7420860000	1.3914460000	2.2644550000
H	-3.7712520000	2.8514820000	2.6671100000
H	-2.8771770000	4.2721740000	0.8299530000
H	-3.9737620000	4.2334700000	-1.4044120000
H	-5.9691750000	2.8075950000	-1.7881640000
H	-9.0179420000	0.5719170000	-1.9680560000
H	-10.9561340000	1.9286690000	-2.6972840000
H	-11.4766750000	4.0724600000	-1.5550550000
H	-10.0299830000	4.8630720000	0.3032070000
H	-8.0707190000	3.5232340000	1.0064320000
H	-7.6290950000	-2.6169990000	-0.9655230000
H	-9.6428770000	-4.0483660000	-0.8175700000
H	-11.6076620000	-3.2999080000	0.5059410000
H	-11.5463610000	-1.1071630000	1.6719500000
H	-9.5458630000	0.3387390000	1.4933430000
S	-0.6509650000	0.4709020000	-1.1424480000
O	-2.0496810000	0.5395210000	-1.9960660000
O	0.4805190000	1.4879360000	-1.5946740000
O	-0.0896420000	-1.0738990000	-1.2410800000
O	-1.1519220000	0.6738570000	0.4267940000
Na	-0.1315640000	-3.1037580000	-1.4783890000
Na	-3.2454360000	1.0980480000	-0.1559030000

S5. Photophysical and AIE studies

S5.1 Fluorescence spectra

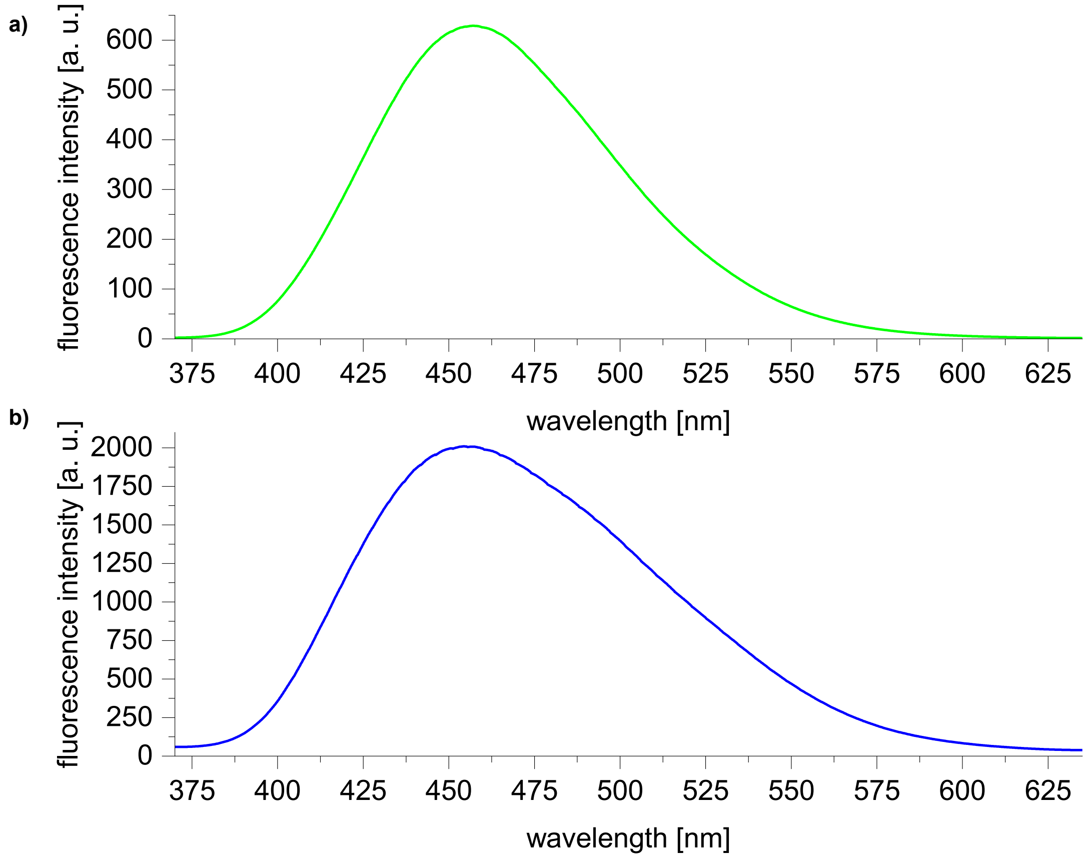

Figure S 28 Solid state fluorescence spectra: a) **1**, b) **2**, experimental conditions:($\lambda_{ex,\mathbf{1}}$ = 326 nm, PMT Voltage = 250V; $\lambda_{ex,\mathbf{2}}$ = 266 nm, PMT Voltage = 400V)

S5.2 AIE-studies – spectrofluorimetry

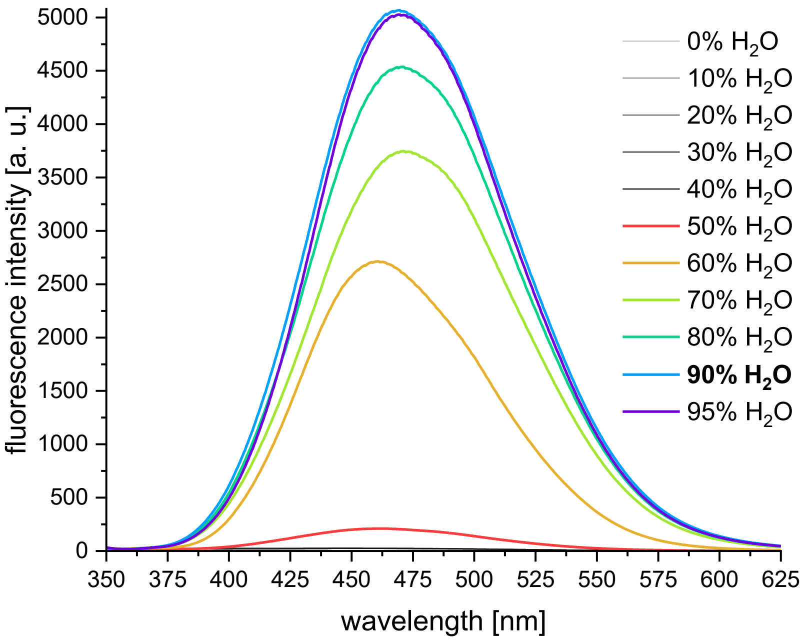

Figure S 29 Fluorescence spectra of **1** in H_2O/THF system containing different vol% of water in the sample ($C = 2 \cdot 10^{-5}$ M, PMT voltage: 400 V).

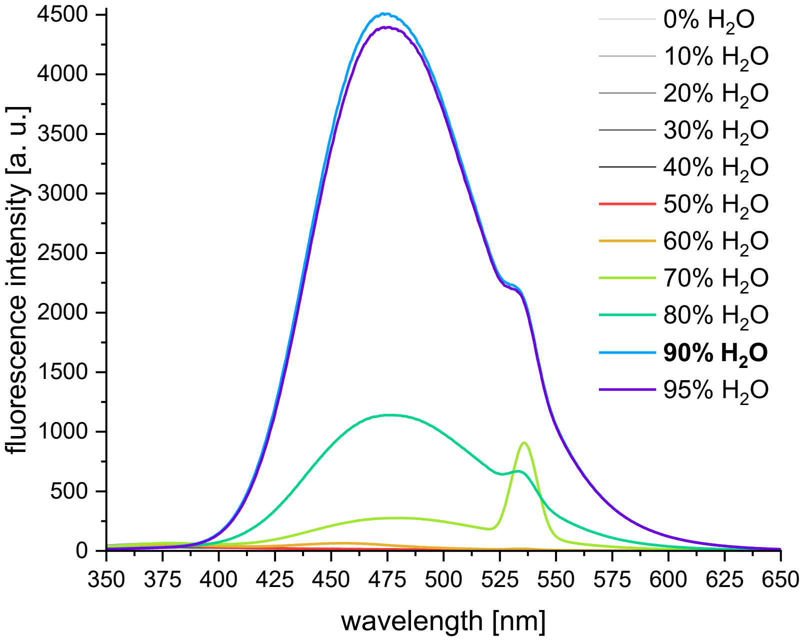

Figure S 30 Fluorescence spectra of **2** in H_2O/THF system containing different vol% of water in the sample ($C = 2 \cdot 10^{-5}$ M, PMT voltage: 400 V).

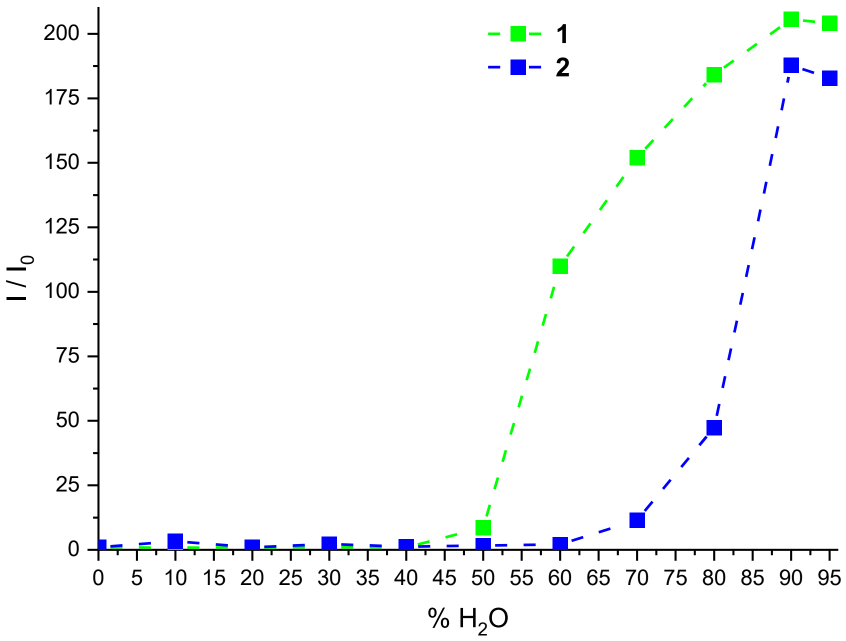

Figure S 31 Fluorescence intensity changes of compounds **1** and **2** in H_2O/THF system containing different vol% of water in the sample ($C = 2 \cdot 10^{-5}$ M; data for the maximum λ_{em}, PMT voltage: 400 V).

S6. Receptor studies – spectrofluorimetry

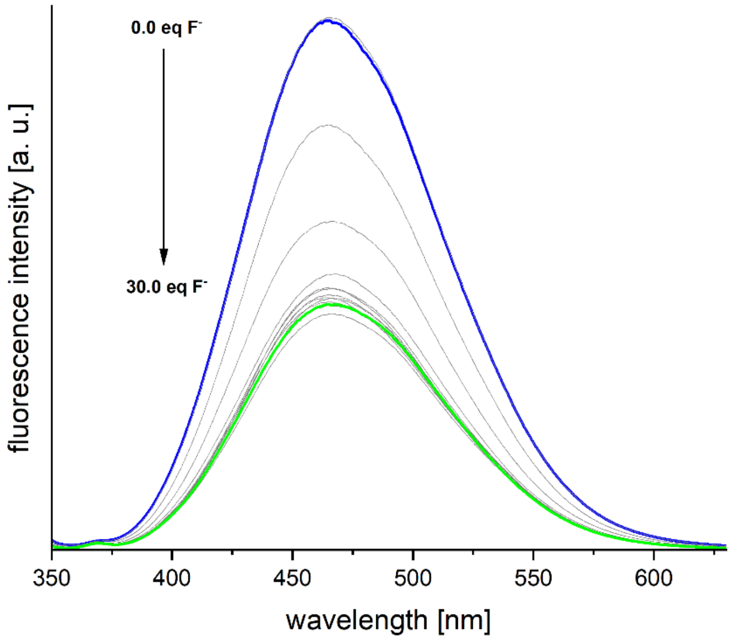

Figure S 32 Emission spectra of aggregated **1** in the presence of increasing molar equivalents of **F**$^-$. Conditions: H_2O:THF = 95:5 *v/v*, $C_1 = 2 \cdot 10^{-5}$ M, λ_{ex} = 326 nm, λ_{em} = 468 nm.

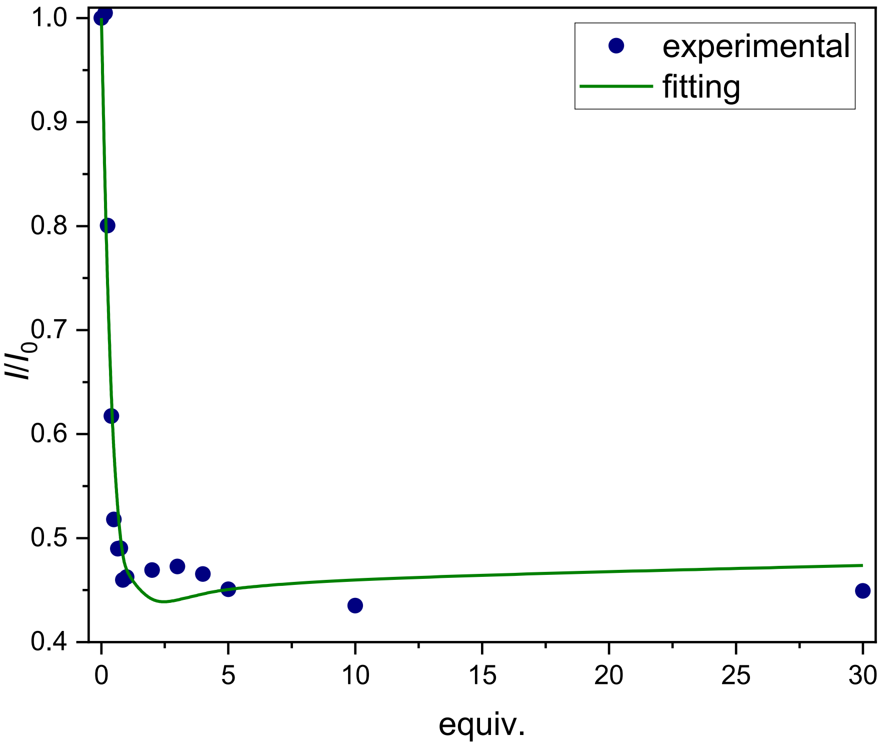

Figure S 33 Titration curve and global fitting (Bindfit) for interactions between **1** and **F**$^-$ in H_2O:THF = 95:5 *v/v* solvent system (model 2:1 statistical, Nelder-Mead method-algorithm, dilution correction, $K_a = (2.24 \pm 0.63) \cdot 10^5$ M^{-1}, covariance = $7.93 \cdot 10^{-2}$).

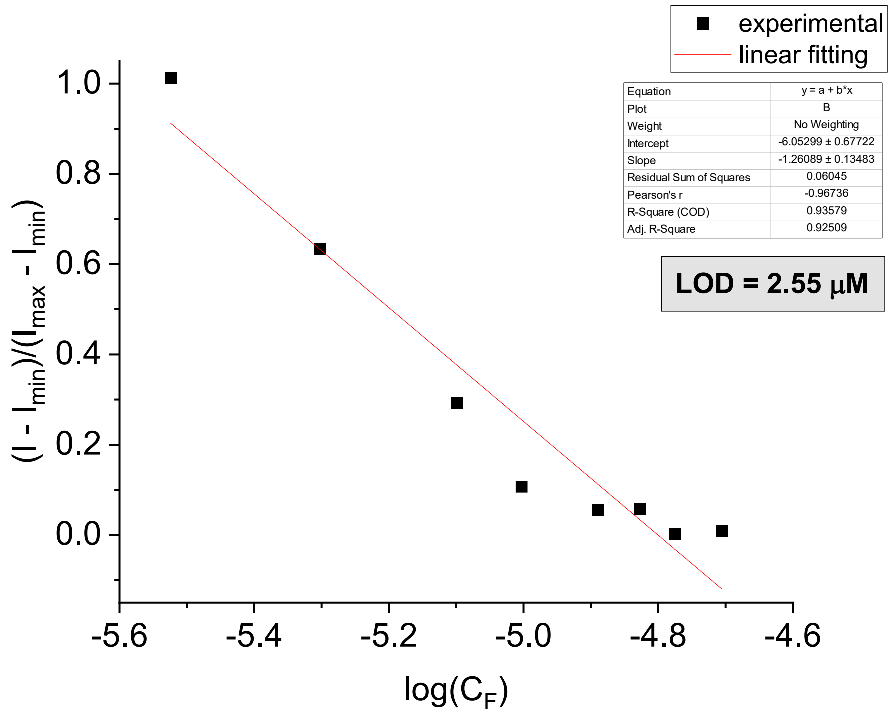

Figure S 34 $(I\text{-}I_{min})/(I_{max}\text{-}I_{min})$ versus log(C_{cation}) plot for the estimation of LOD for the interactions between 1 and F^-. The linear fit data and calculated LOD are presented in the figure.

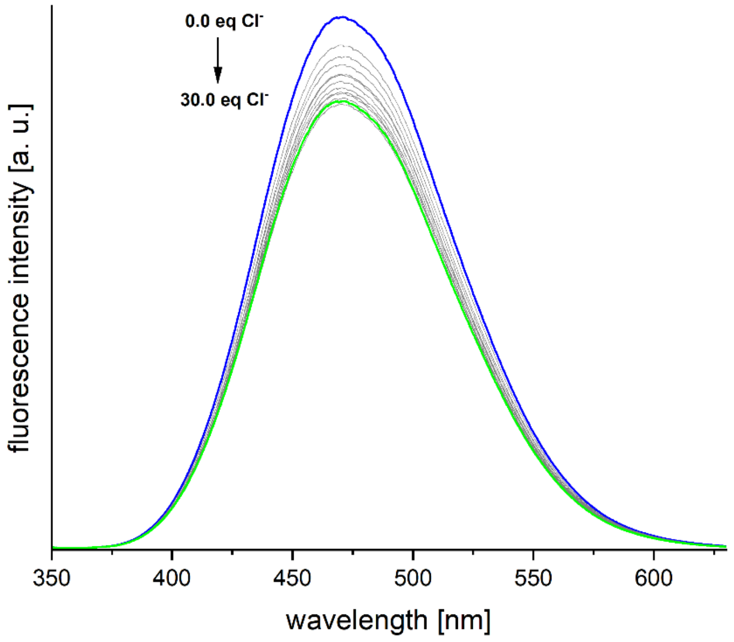

Figure S 35 Emission spectra of aggregated **1** in the presence of increasing molar equivalents of $\mathbf{Cl^-}$. Conditions: H_2O:THF = 95:5 *v/v*, $C_1 = 2{\cdot}10^{-5}$ M, λ_{ex} = 326 nm, λ_{em} = 468 nm.

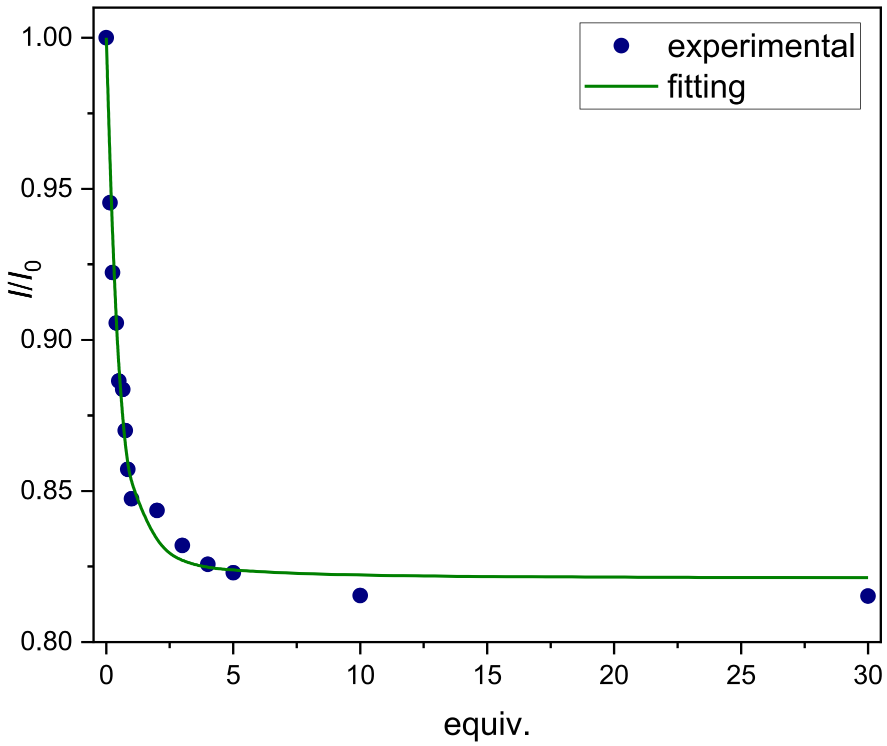

Figure S 36 Titration curve and global fitting (Bindfit) for interactions between **1** and **Cl**$^-$ in H_2O:THF = 95:5 *v/v* solvent system (model 2:1 non-cooperative, Nelder-Mead method-algorithm, dilution correction, K_a = (2.53±0.46)·10^5 M^{-1}, covariance = 1.75·10^{-2}).

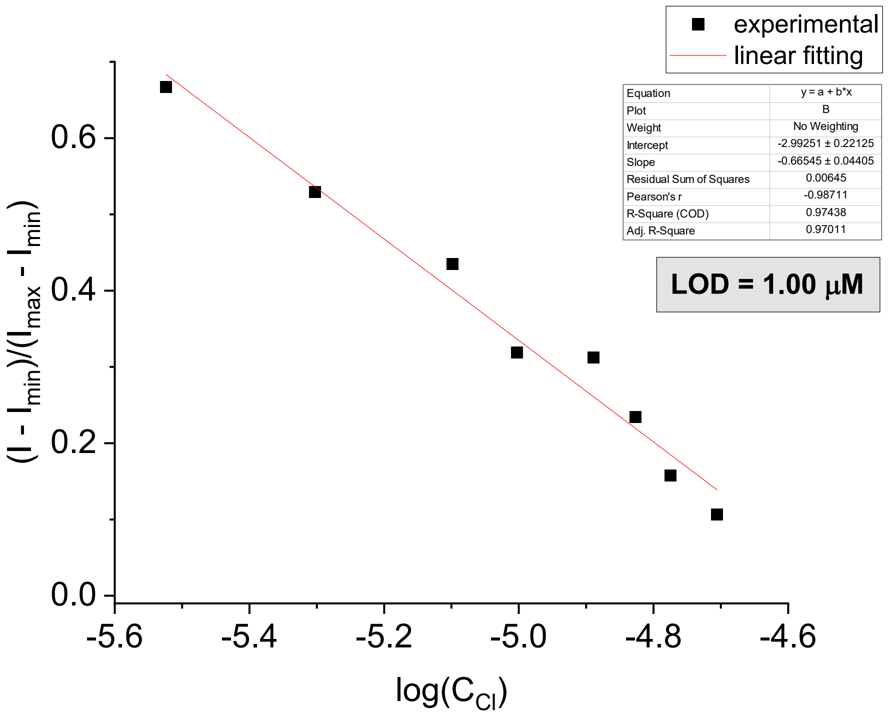

Figure S 37 (I-I_{min})/(I_{max}-I_{min}) versus log(C_{cation}) plot for the estimation of LOD for the interactions between **1** and **Cl**$^-$. The linear fit data and calculated LOD are presented in the figure.

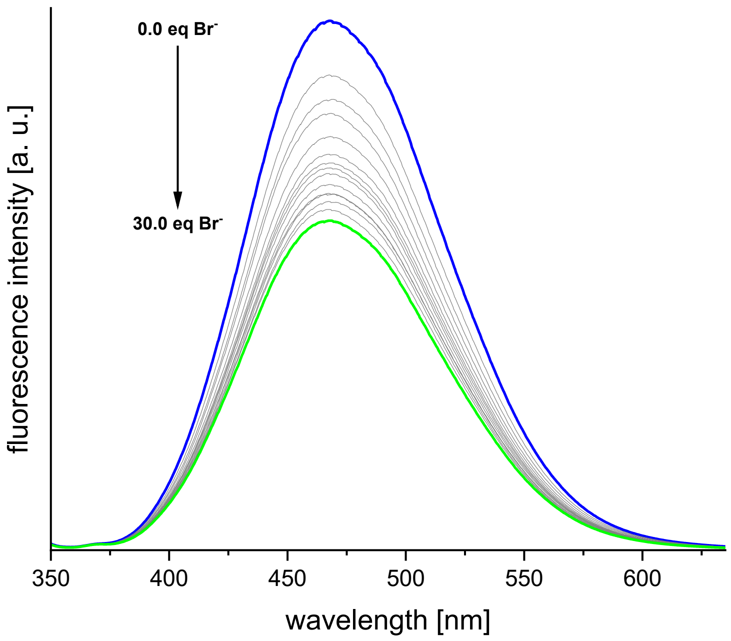

Figure S 38 Emission spectra of aggregated **1** in the presence of increasing molar equivalents of **Br**$^-$. Conditions: H_2O:THF = 95:5 *v/v*, $C_1 = 2 \cdot 10^{-5}$ M, λ_{ex} = 326 nm, λ_{em} = 468 nm.

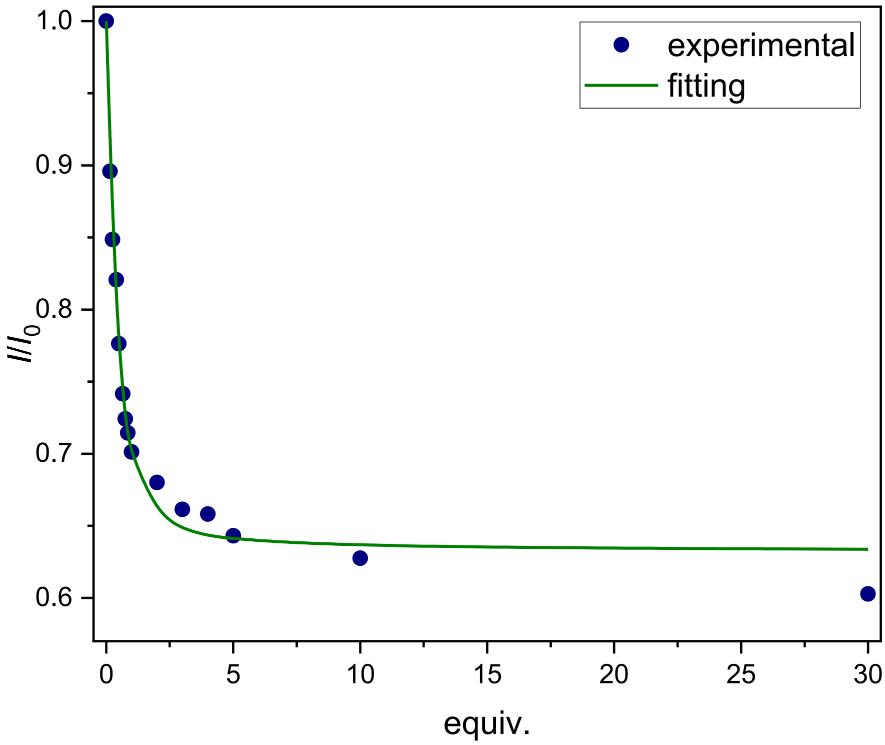

Figure S 39 Titration curve and global fitting (Bindfit) for interactions between **1** and **Br**$^-$ in H_2O:THF = 95:5 *v/v* solvent system (model 2:1 non-cooperative, Nelder-Mead method-algorithm, dilution correction, K_a = $(3.52 \pm 0.84) \cdot 10^5$ M^{-1}, covariance = $1.69 \cdot 10^{-2}$).

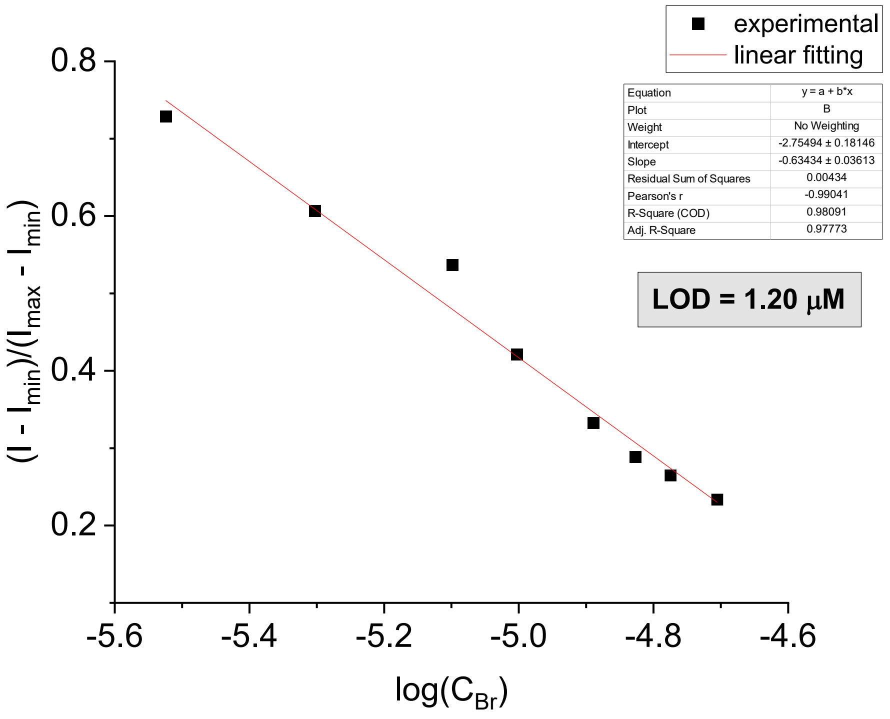

Figure S 40 (I-I_{min})/(I_{max}-I_{min}) versus log(C_{cation}) plot for the estimation of LOD for the interactions between **1** and **Br**$^{-}$. The linear fit data and calculated LOD are presented in the figure.

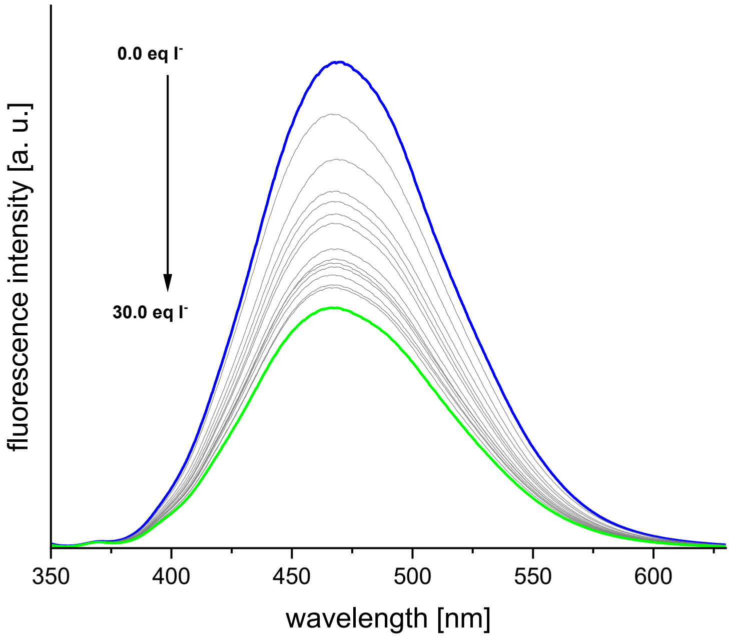

Figure S 41 Emission spectra of aggregated **1** in the presence of increasing molar equivalents of **I**$^{-}$. Conditions: H_2O:THF = 95:5 v/v, $C_1 = 2\cdot10^{-5}$ M, λ_{ex} = 326 nm, λ_{em} = 468 nm.

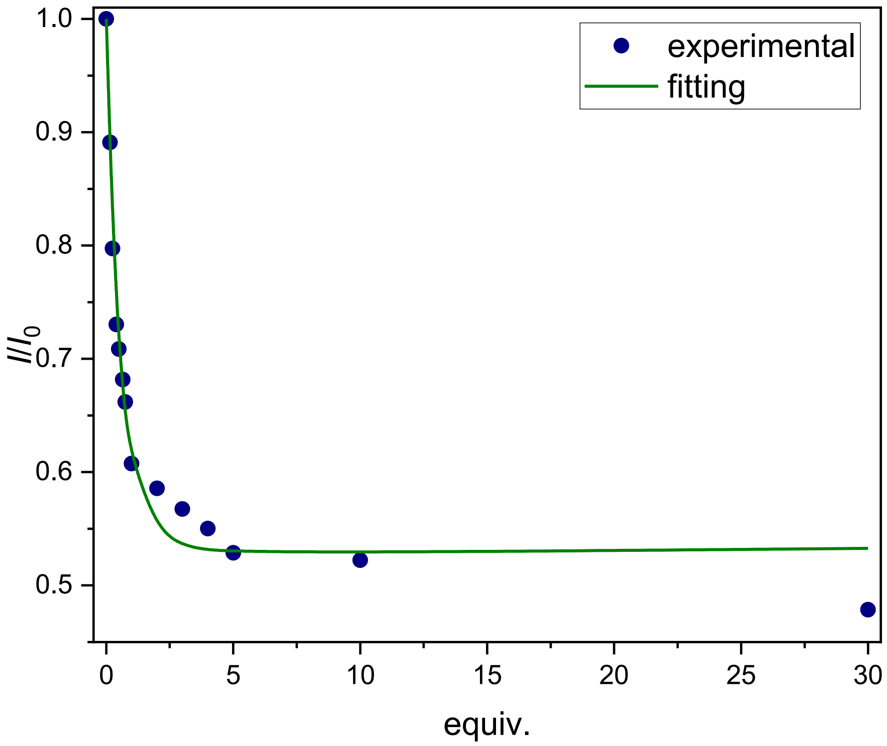

Figure S 42 Titration curve and global fitting (Bindfit) for interactions between **1** and **I**$^-$ in H_2O:THF = 95:5 *v/v* solvent system (model 2:1 statistical, Nelder-Mead method-algorithm, dilution correction, $K_a = (1.22 \pm 0.17) \cdot 10^5$ M^{-1}, covariance = $2.62 \cdot 10^{-2}$).

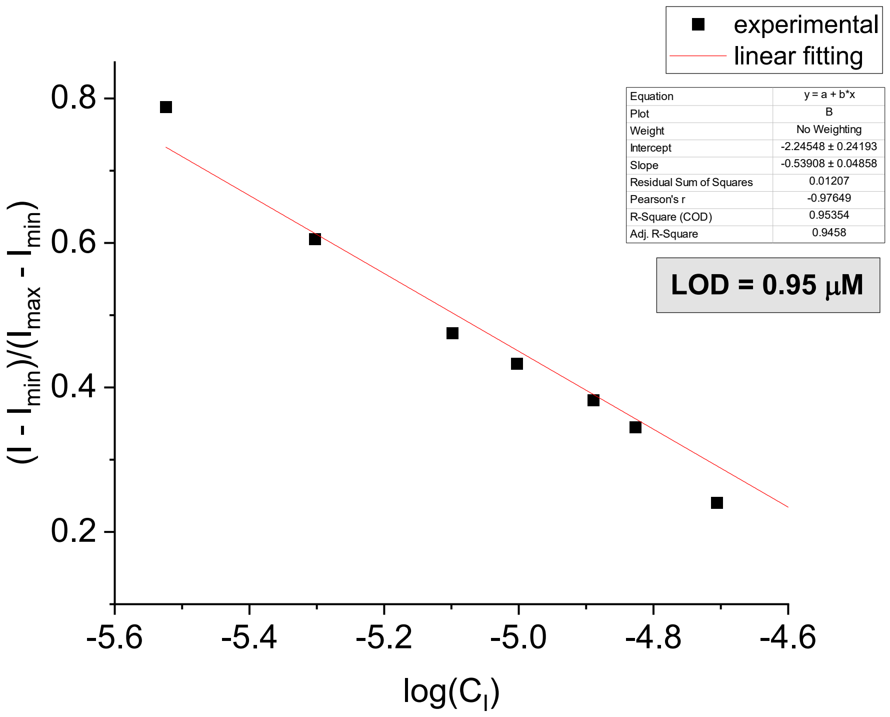

Figure S 43 $(I\text{-}I_{min})/(I_{max}\text{-}I_{min})$ versus $\log(C_{cation})$ plot for the estimation of LOD for the interactions between **1** and **I**$^-$. The linear fit data and calculated LOD are presented in the figure.

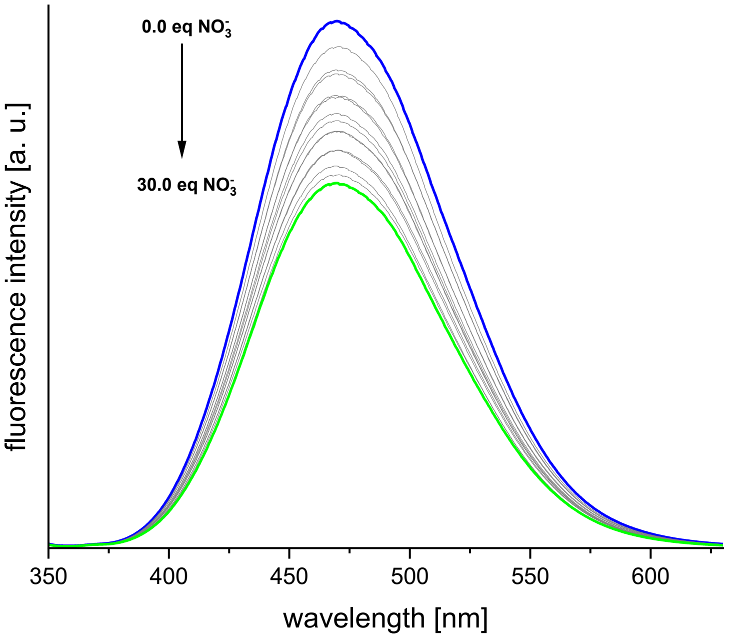

Figure S 44 Emission spectra of aggregated **1** in the presence of increasing molar equivalents of $\mathbf{NO_3^-}$. Conditions: H_2O:THF = 95:5 *v/v*, $C_1 = 2\cdot10^{-5}$ M, λ_{ex} = 326 nm, λ_{em} = 468 nm.

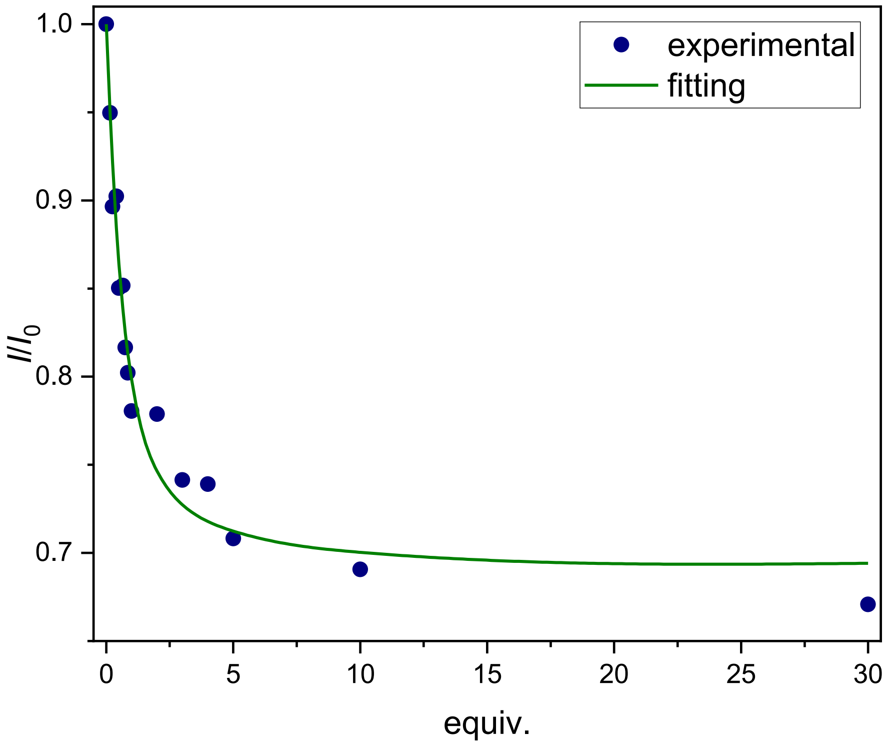

Figure S 45 Titration curve and global fitting (Bindfit) for interactions between **1** and $\mathbf{NO_3^-}$ in H_2O:THF = 95:5 *v/v* solvent system (model 2:1 non-cooperative, Nelder-Mead method-algorithm, dilution correction, $K_a = (1.47\pm0.41)\cdot10^5$ M^{-1}, covariance = $3.16\cdot10^{-2}$).

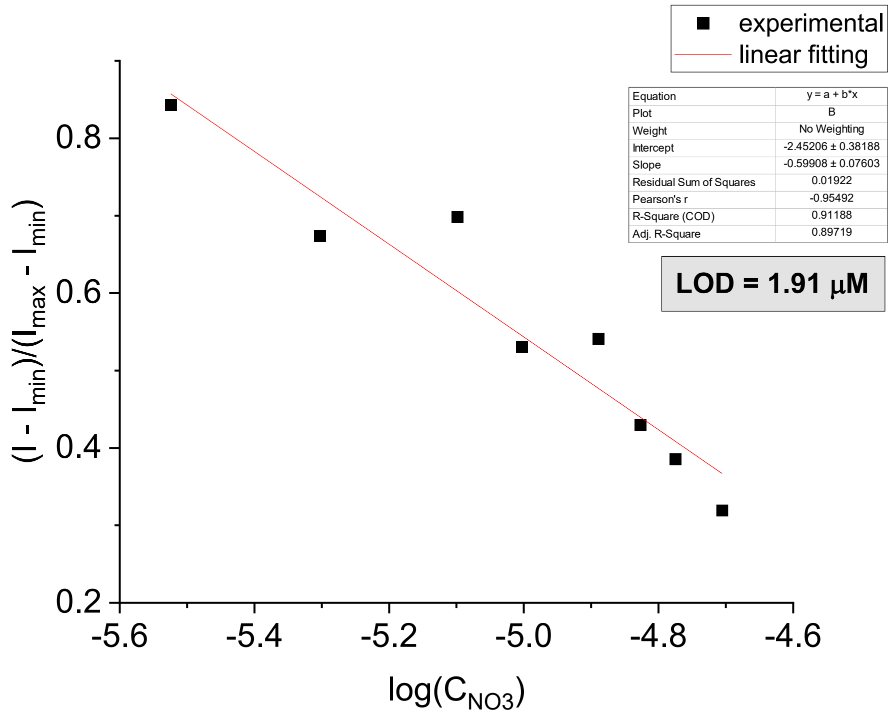

Figure S 46 (I-I_{min})/(I_{max}-I_{min}) versus log(C_{cation}) plot for the estimation of LOD for the interactions between **1** and **NO_3^-**. The linear fit data and calculated LOD are presented in the figure.

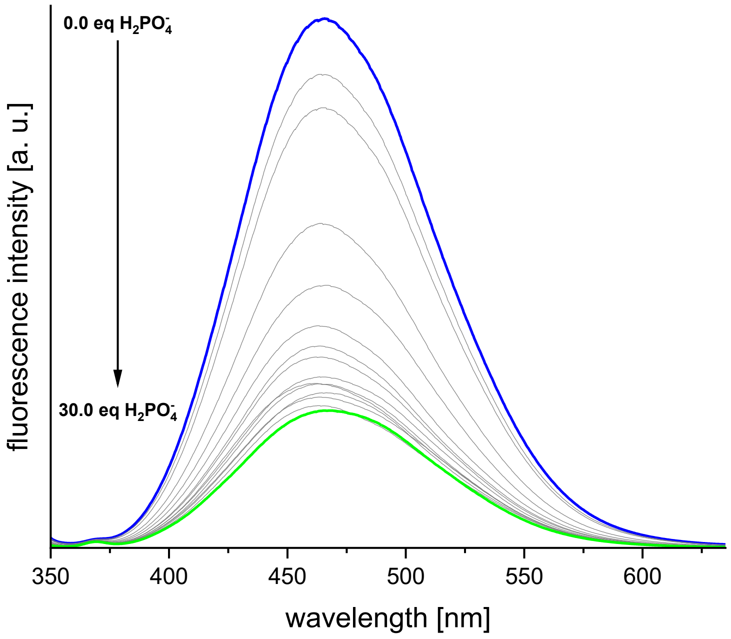

Figure S 47 Emission spectra of aggregated **1** in the presence of increasing molar equivalents of **$H_2PO_4^-$**. Conditions: H_2O:THF = 95:5 *v/v*, $C_1 = 2{\cdot}10^{-5}$ M, λ_{ex} = 326 nm, λ_{em} = 468 nm.

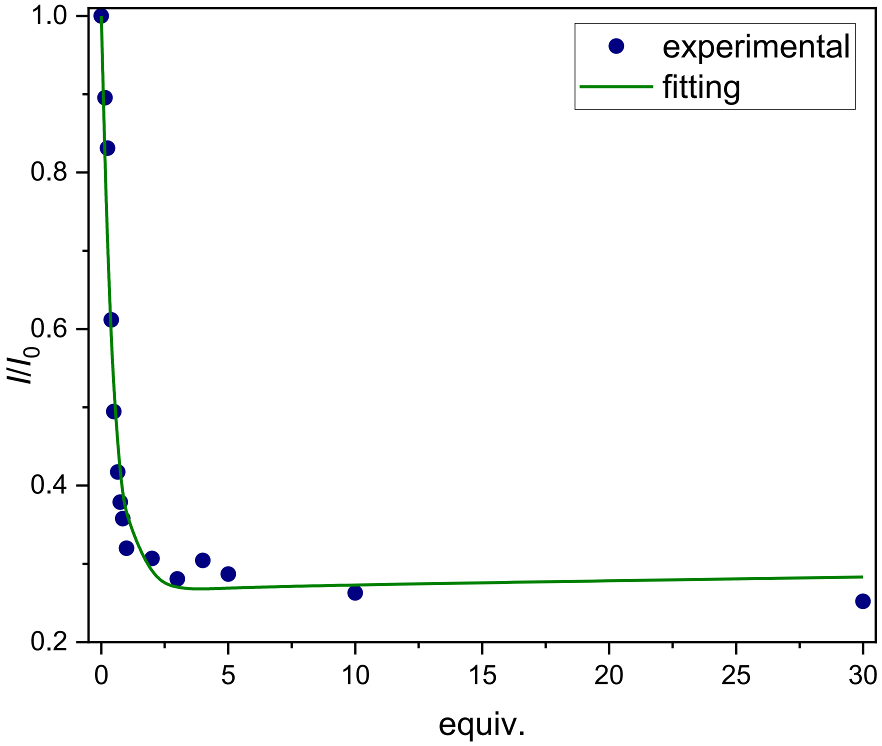

Figure S 48 Titration curve and global fitting (Bindfit) for interactions between **1** and **$H_2PO_4^-$** in H_2O:THF = 95:5 *v/v* solvent system (model 2:1 statistical, Nelder-Mead method-algorithm, dilution correction, K_a = $(1.49 \pm 0.25) \cdot 10^5$ M^{-1}, covariance = $3.65 \cdot 10^{-2}$).

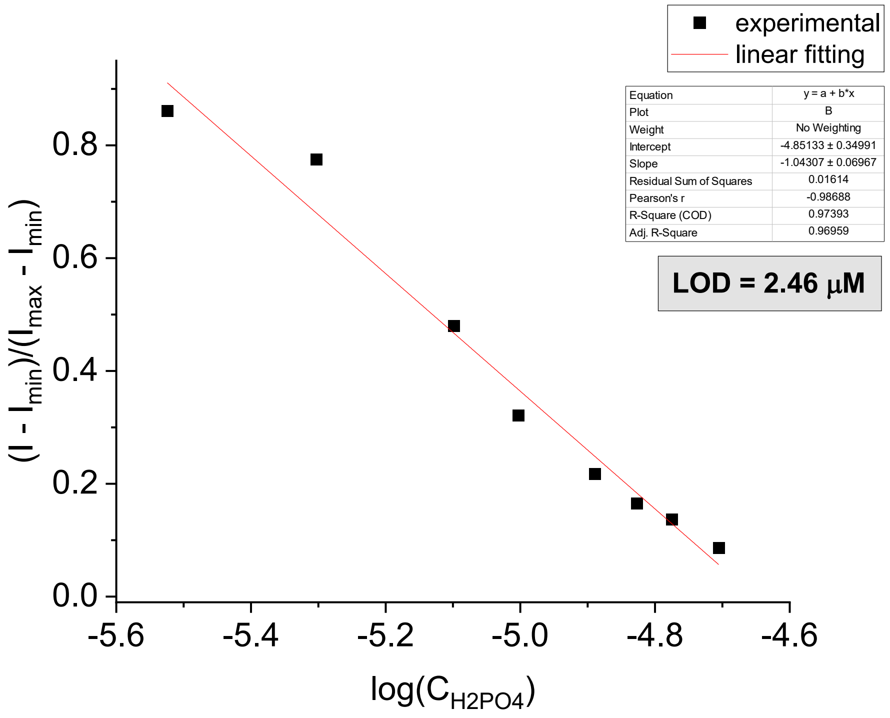

Figure S 49 $(I\text{-}I_{min})/(I_{max}\text{-}I_{min})$ versus log(C_{cation}) plot for the estimation of LOD for the interactions between **1** and **$H_2PO_4^-$**. The linear fit data and calculated LOD are presented in the figure.

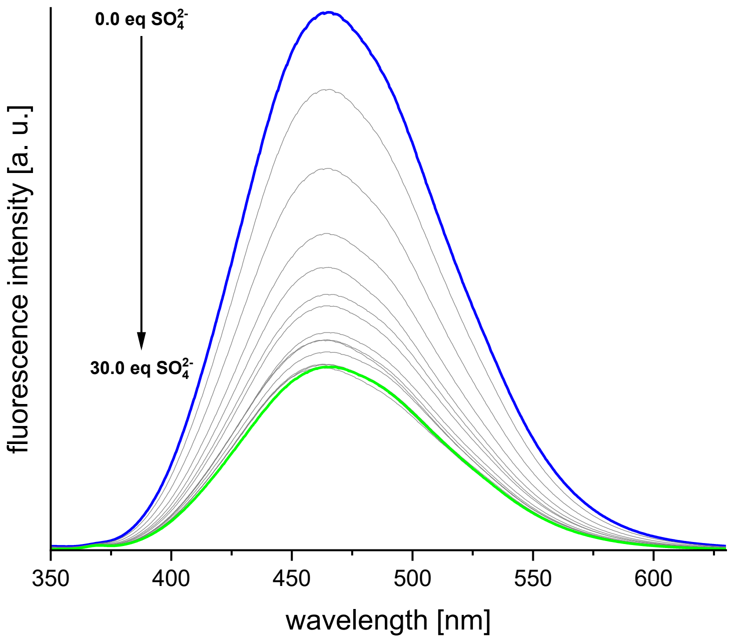

Figure S 50 Emission spectra of aggregated **1** in the presence of increasing molar equivalents of $\mathbf{SO_4^{2-}}$. Conditions: H_2O:THF = 95:5 *v/v*, $C_1 = 2{\cdot}10^{-5}$ M, λ_{ex} = 326 nm, λ_{em} = 468 nm.

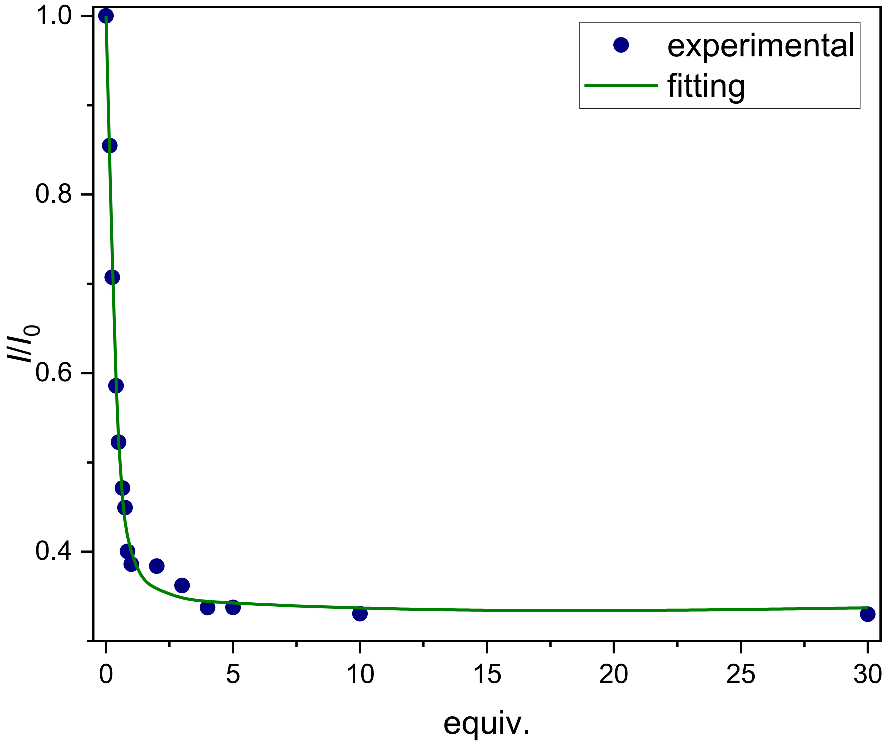

Figure S 51 Titration curve and global fitting (Bindfit) for interactions between **1** and $\mathbf{SO_4^{2-}}$ in H_2O:THF = 95:5 *v/v* solvent system (model 2:1 non-cooperative, Nelder-Mead method-algorithm, dilution correction, K_a = $(1.48\pm0.41){\cdot}10^6$ M^{-1}, covariance = $4.69{\cdot}10^{-3}$).

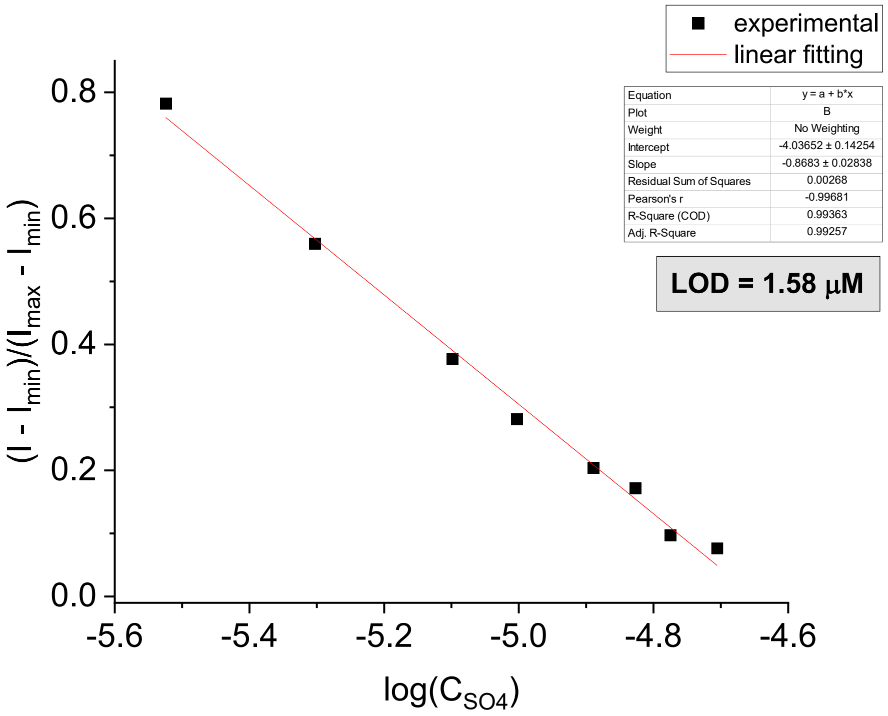

Figure S 52 $(I\text{-}I_{min})/(I_{max}\text{-}I_{min})$ versus log(C_{cation}) plot for the estimation of LOD for the interactions between **1** and $\mathbf{SO_4^{2-}}$. The linear fit data and calculated LOD are presented in the figure.

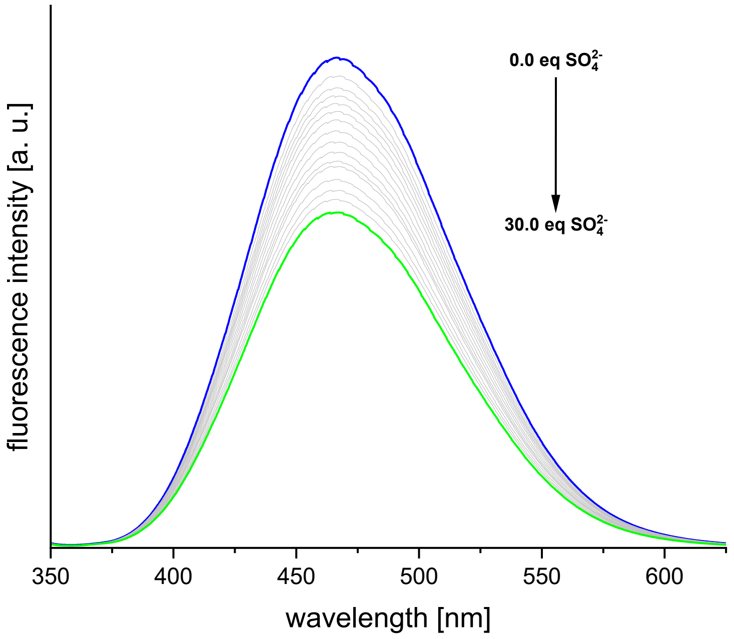

Figure S 53 Emission spectra of aggregated **1** in the presence of increasing molar equivalents of $\mathbf{SO_4^{2-}}$ in buffer (pH 5.1): THF = 95:5 *v/v* solvent system. Conditions: $C_1 = 2{\cdot}10^{-5}$ M, λ_{ex} = 326 nm, λ_{em} = 468 nm.

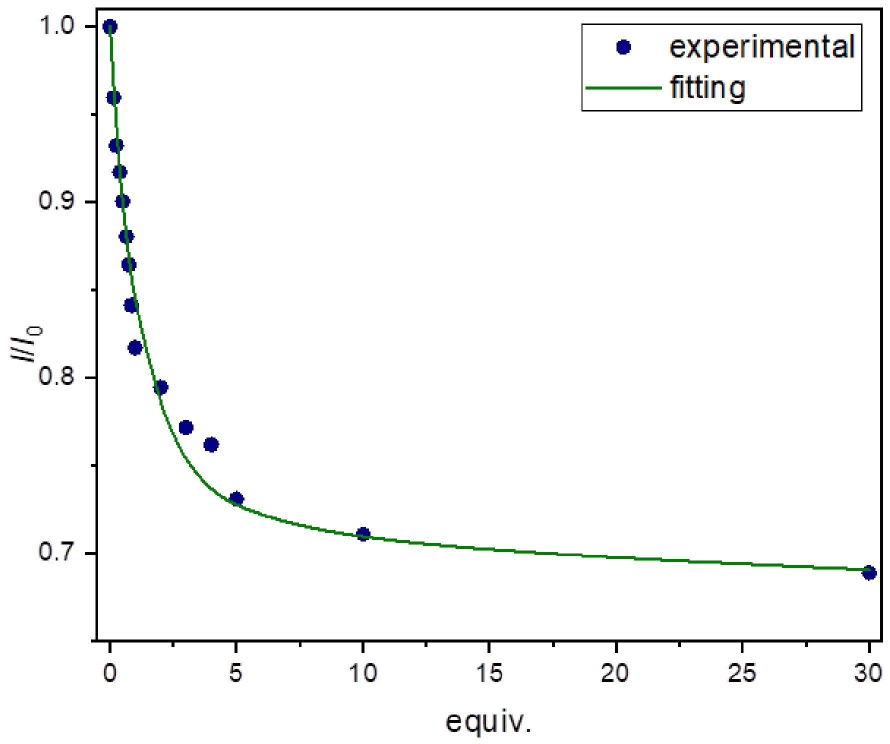

Figure S 54 Titration curve and global fitting (Bindfit) for interactions between aggregated **1** and $\mathbf{SO_4^{2-}}$ in buffer (pH 5.1) : THF = 95:5 *v/v* solvent system (model 2:1 statistical, Nelder-Mead method-algorithm, dilution correction, K_a = (4.32±0.57)·10^4 M^{-1}, covariance = 2.17·10^{-2}).

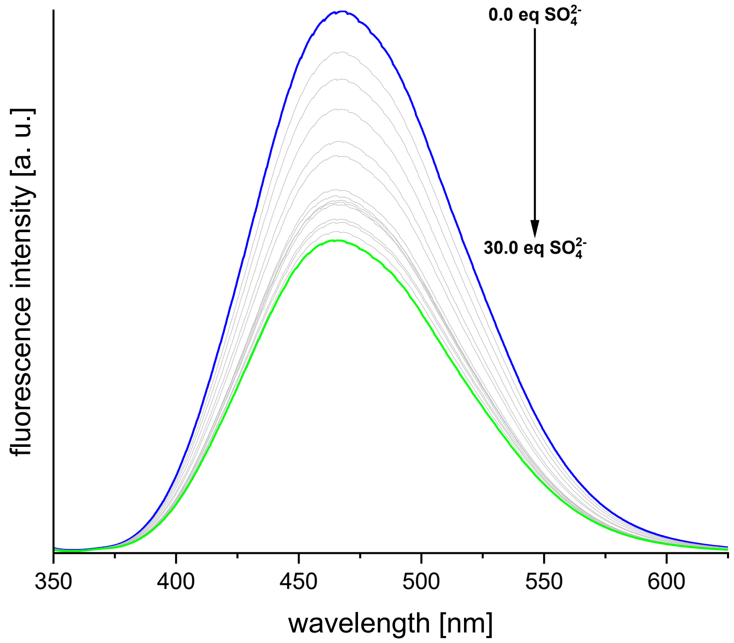

Figure S 55 Emission spectra of aggregated **1** in the presence of increasing molar equivalents of $\mathbf{SO_4^{2-}}$ in buffer (pH 7.4) : THF = 95:5 *v/v* solvent system. Conditions: $C_1 = 2\cdot10^{-5}$ M, λ_{ex} = 326 nm, λ_{em} = 468 nm.

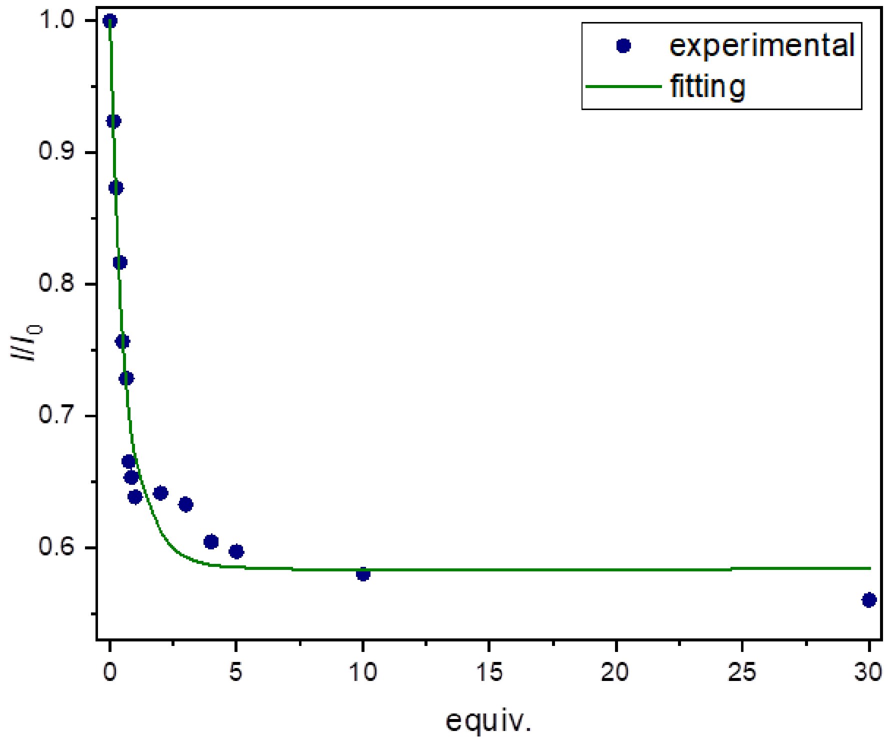

Figure S 56 Titration curve and global fitting (Bindfit) for interactions between aggregated **1** and $\mathbf{SO_4^{2-}}$ in buffer (pH 7.4): THF = 95:5 *v/v* solvent system (model 2:1 statistical, Nelder-Mead method-algorithm, dilution correction, $K_a = (1.13 \pm 0.17) \cdot 10^5\ M^{-1}$, covariance = $3.33 \cdot 10^{-2}$).

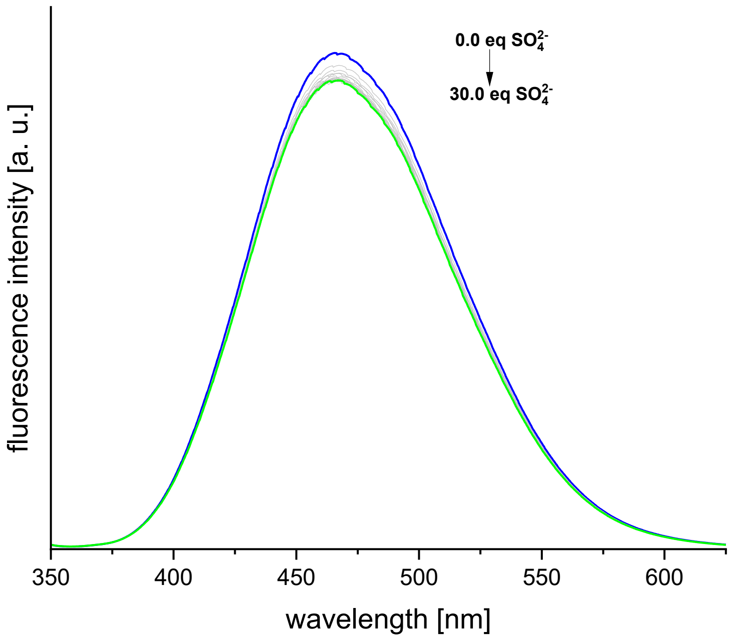

Figure S 57 Emission spectra of aggregated **1** in the presence of increasing molar equivalents of $\mathbf{SO_4^{-}}$ in buffer (pH 8.2) : THF = 95:5 *v/v* solvent system. Conditions: $C_1 = 2 \cdot 10^{-5}$ M, $\lambda_{ex} = 326$ nm, $\lambda_{em} = 468$ nm.

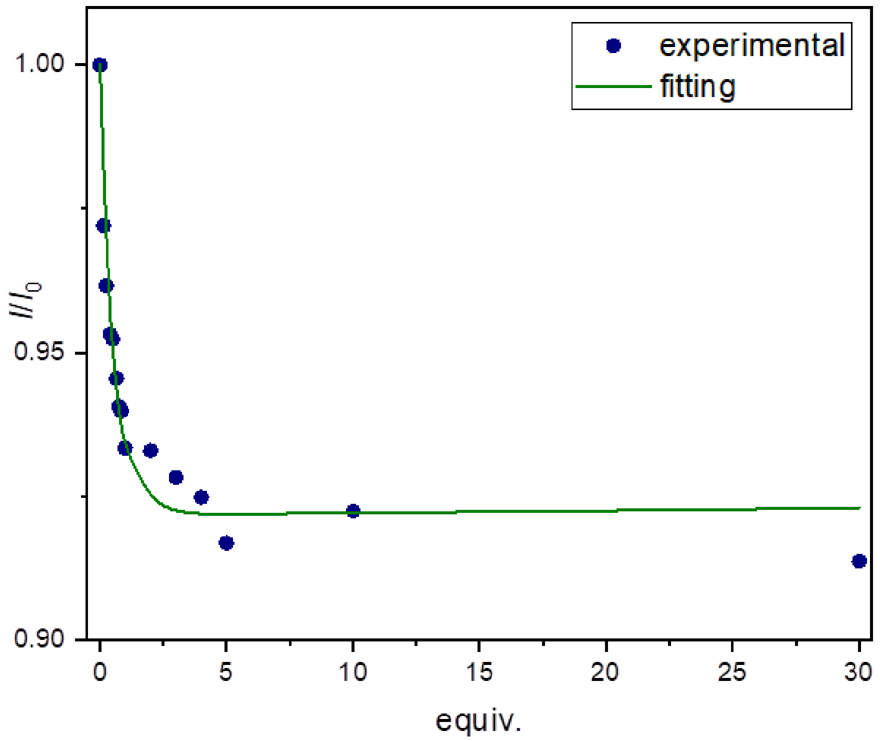

Figure S 58 Titration curve and global fitting (Bindfit) for interactions between aggregated **1** and $\mathbf{SO_4^{2-}}$ in buffer (pH 8.2) : THF = 95:5 *v/v* solvent system (model 2:1 statistical, Nelder-Mead method-algorithm, dilution correction, $K_a = (1.35{\pm}0.25){\cdot}10^5\ M^{-1}$, covariance = $5.50{\cdot}10^{-2}$).

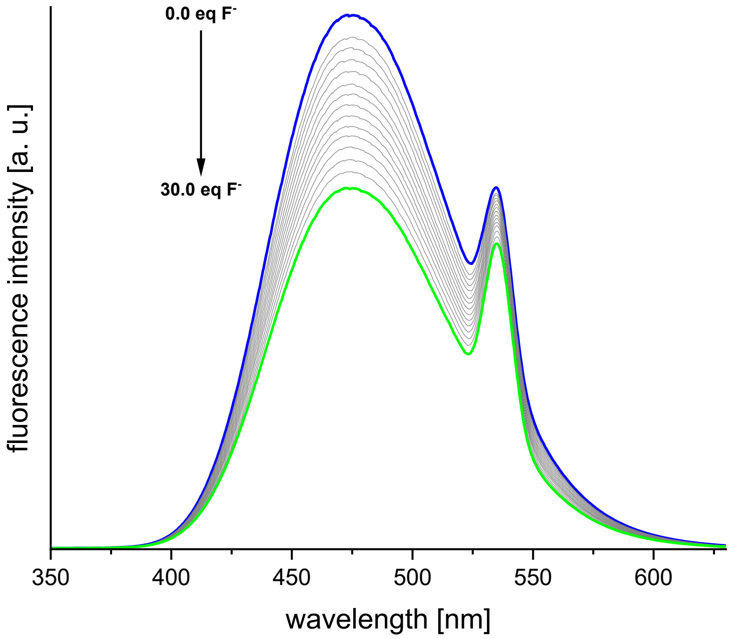

Figure S 59 Emission spectra of aggregated **2** in the presence of increasing molar equivalents of $\mathbf{F^-}$. Conditions: H_2O:THF = 95:5 *v/v*, $C_1 = 2{\cdot}10^{-5}$ M, λ_{ex} = 266 nm, λ_{em} = 475 nm.

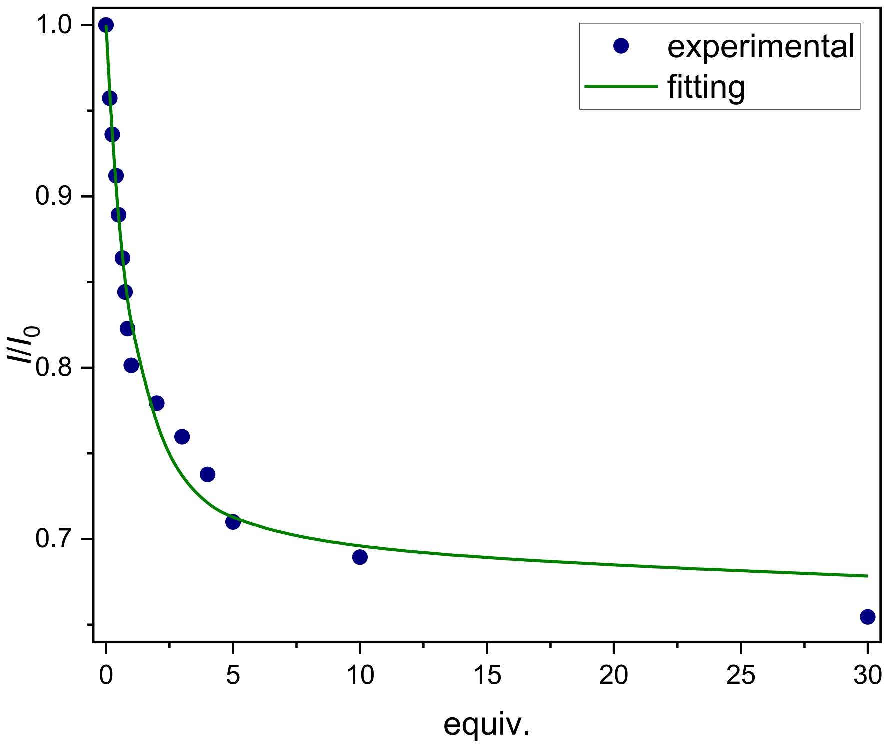

Figure S 60 Titration curve and global fitting (Bindfit) for interactions between **2** and **F**⁻ in H_2O:THF = 95:5 *v/v* solvent system (model 2:1 statistical, Nelder-Mead method-algorithm, dilution correction, $K_a = (8.20 \pm 1.55) \cdot 10^4$ M^{-1}, covariance = $1.79 \cdot 10^{-2}$).

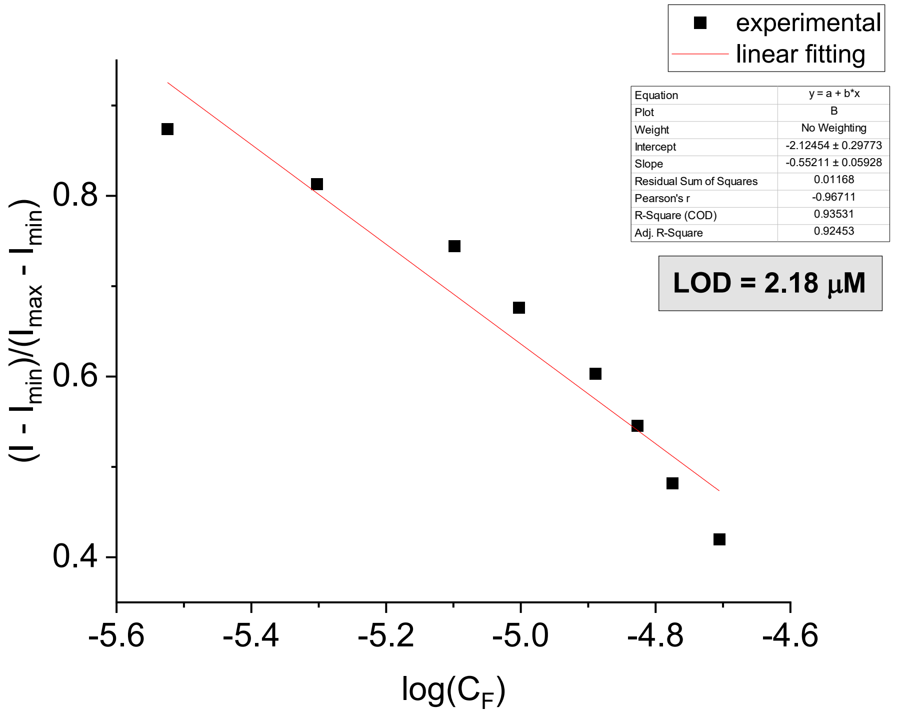

Figure S 61 $(I-I_{min})/(I_{max}-I_{min})$ versus log(C_{cation}) plot for the estimation of LOD for the interactions between **2** and **F**⁻. The linear fit data and calculated LOD are presented in the figure.

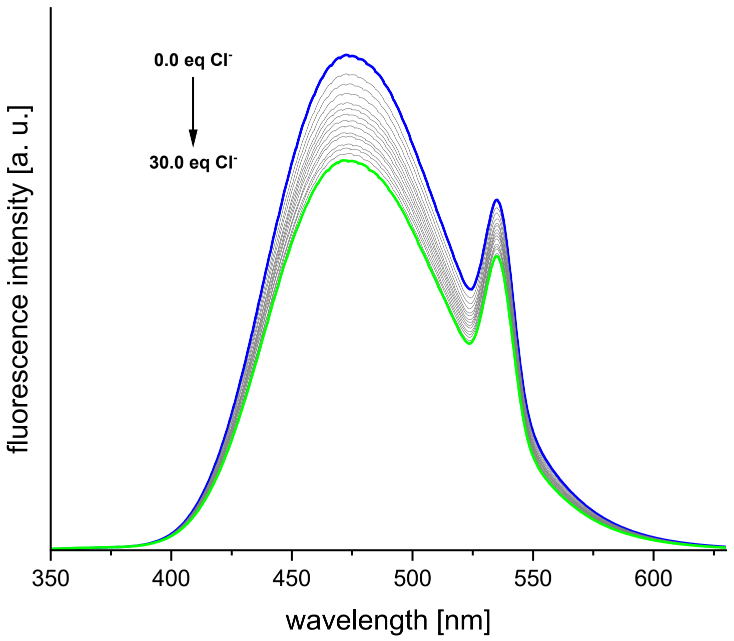

Figure S 62 Emission spectra of aggregated **2** in the presence of increasing molar equivalents of **Cl⁻**. Conditions: H_2O:THF = 95:5 *v/v*, $C_1 = 2{\cdot}10^{-5}$ M, λ_{ex} = 266 nm, λ_{em} = 475 nm.

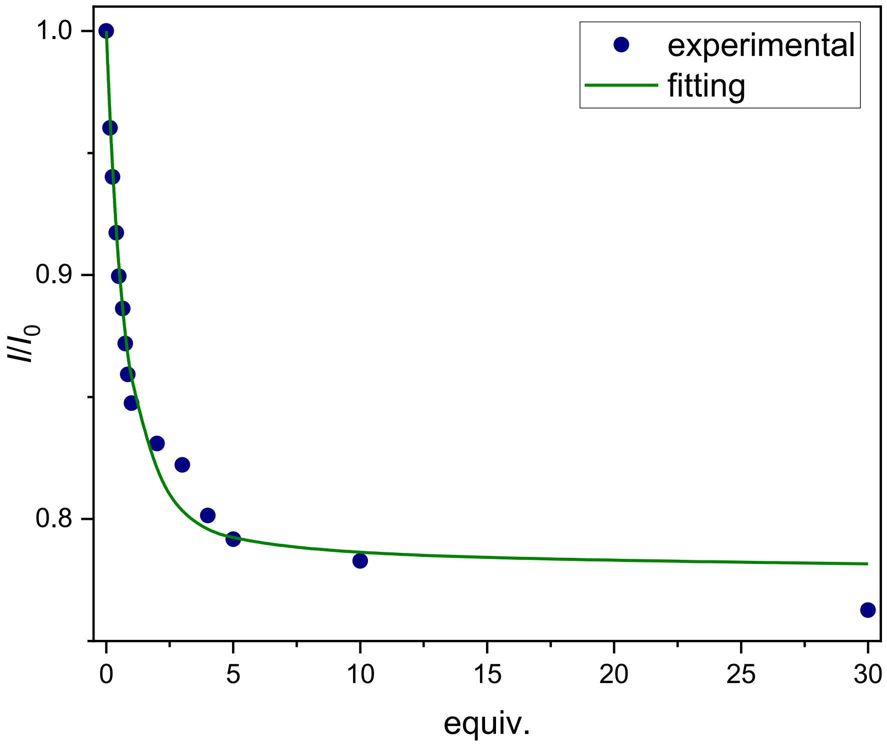

Figure S 63 Titration curve and global fitting (Bindfit) for interactions between **2** and **Cl⁻** in H_2O:THF = 95:5 *v/v* solvent system (model 2:1 statistical, Nelder-Mead method-algorithm, dilution correction, K_a = $(7.10{\pm}0.78){\cdot}10^4$ M^{-1}, covariance = $1.76{\cdot}10^{-2}$).

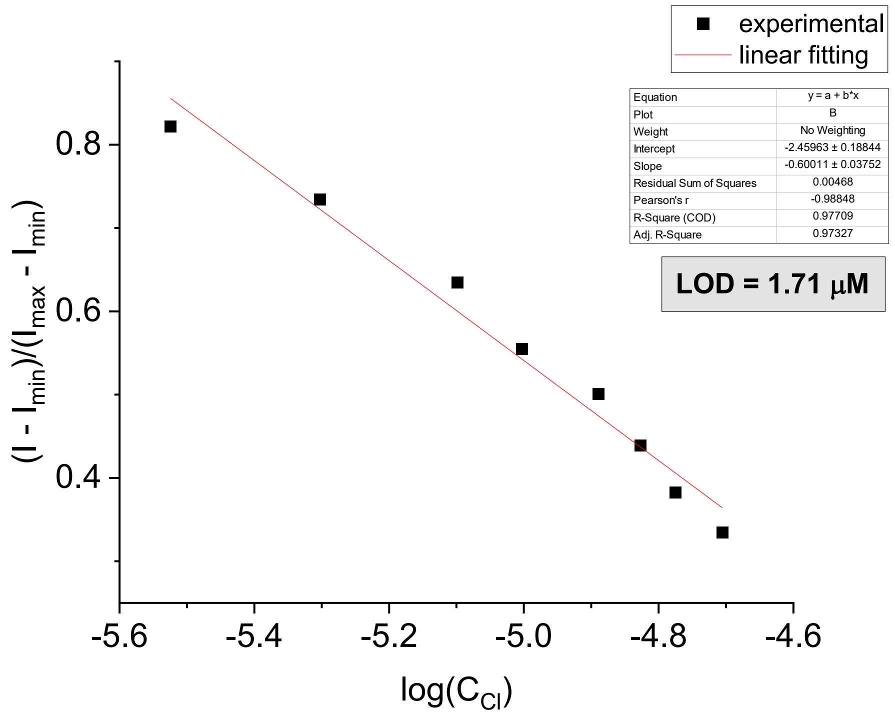

Figure S 64 (I-I_{min})/(I_{max}-I_{min}) versus log(C_{cation}) plot for the estimation of LOD for the interactions between **2** and **Cl**$^{-}$. The linear fit data and calculated LOD are presented in the figure.

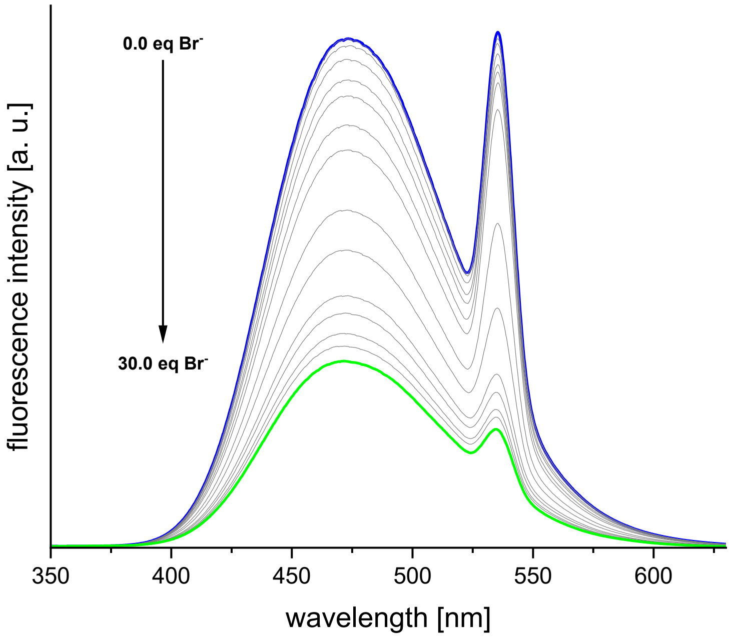

Figure S 65 Emission spectra of aggregated **2** in the presence of increasing molar equivalents of **Br**$^{-}$. Conditions: H_2O:THF = 95:5 *v/v*, $C_1 = 2{\cdot}10^{-5}$ M, λ_{ex} = 266 nm, λ_{em} = 475 nm.

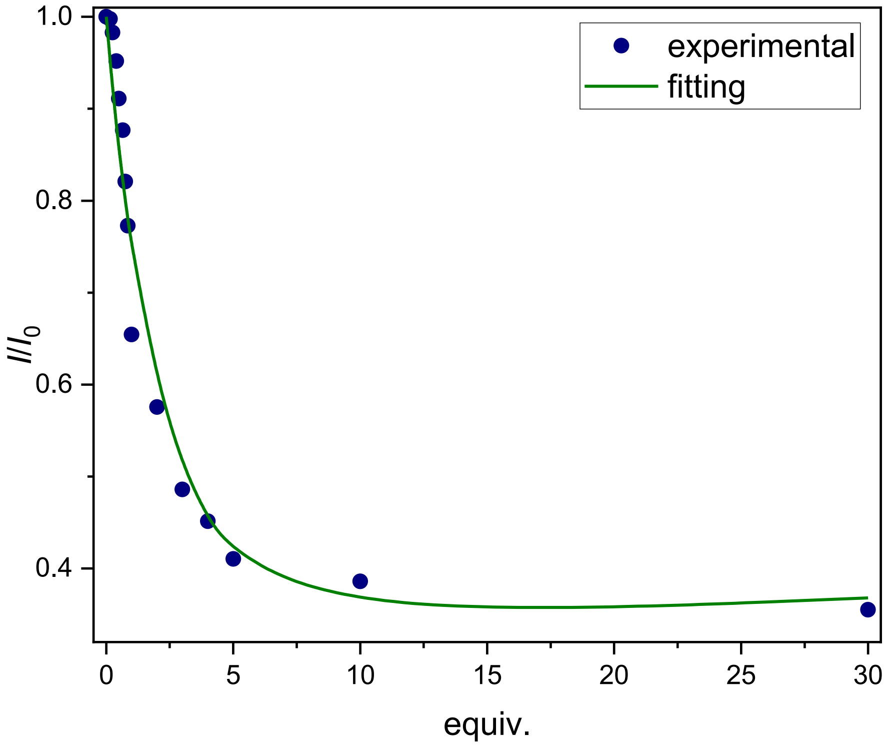

Figure S 66 Titration curve and global fitting (Bindfit) for interactions between **2** and **Br**$^{-}$ in H_2O:THF = 95:5 *v/v* solvent system (model 2:1 non-cooperative, Nelder-Mead method-algorithm, dilution correction, K_a = $(9.74\pm0.88)\cdot10^3$ M^{-1}, covariance = $3.27\cdot10^{-2}$).

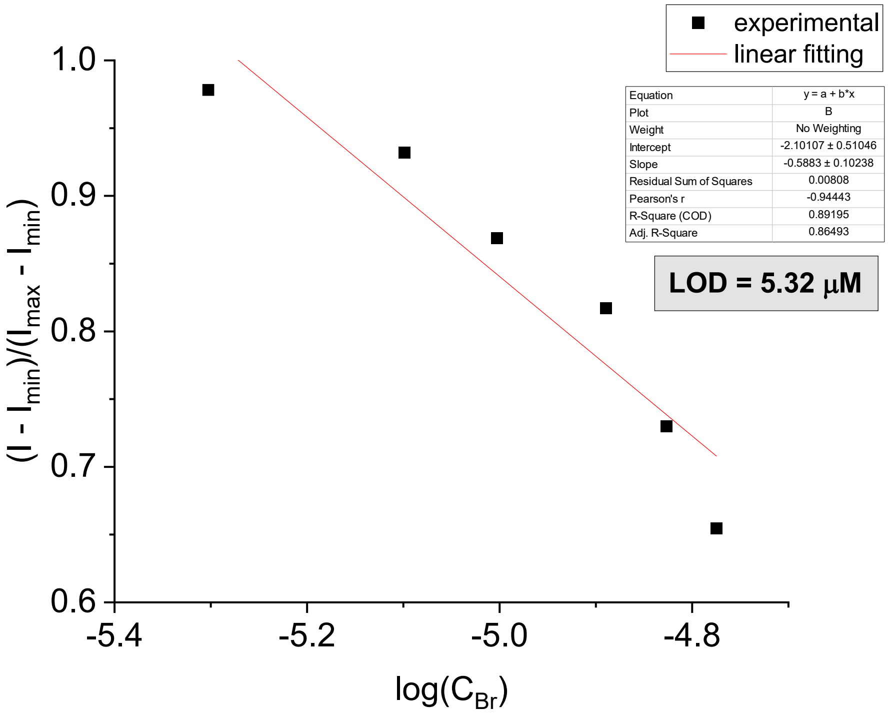

Figure S 67 $(I\text{-}I_{min})/(I_{max}\text{-}I_{min})$ versus $\log(C_{cation})$ plot for the estimation of LOD for the interactions between **2** and **Br**$^{-}$. The linear fit data and calculated LOD are presented in the figure.

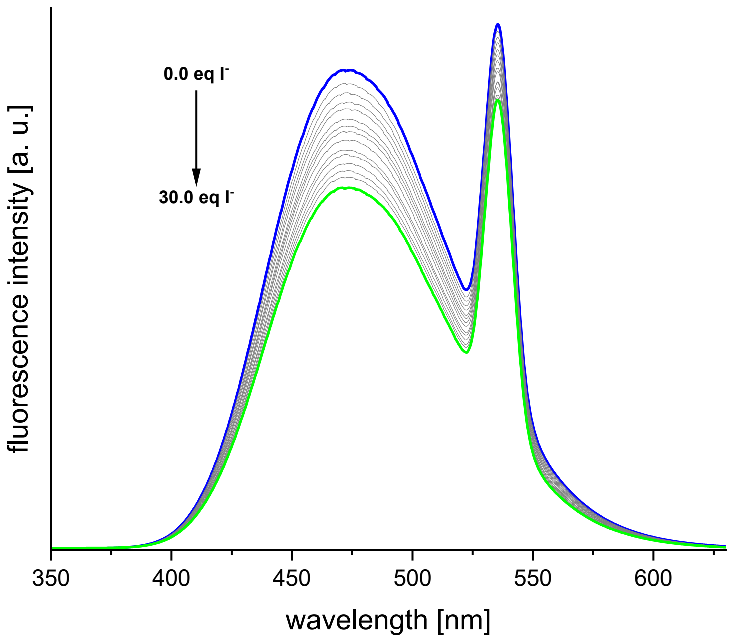

Figure S 68 Emission spectra of aggregated **2** in the presence of increasing molar equivalents of **I**$^-$. Conditions: H_2O:THF = 95:5 *v/v*, $C_I = 2\cdot10^{-5}$ M, λ_{ex} = 266 nm, λ_{em} = 475 nm.

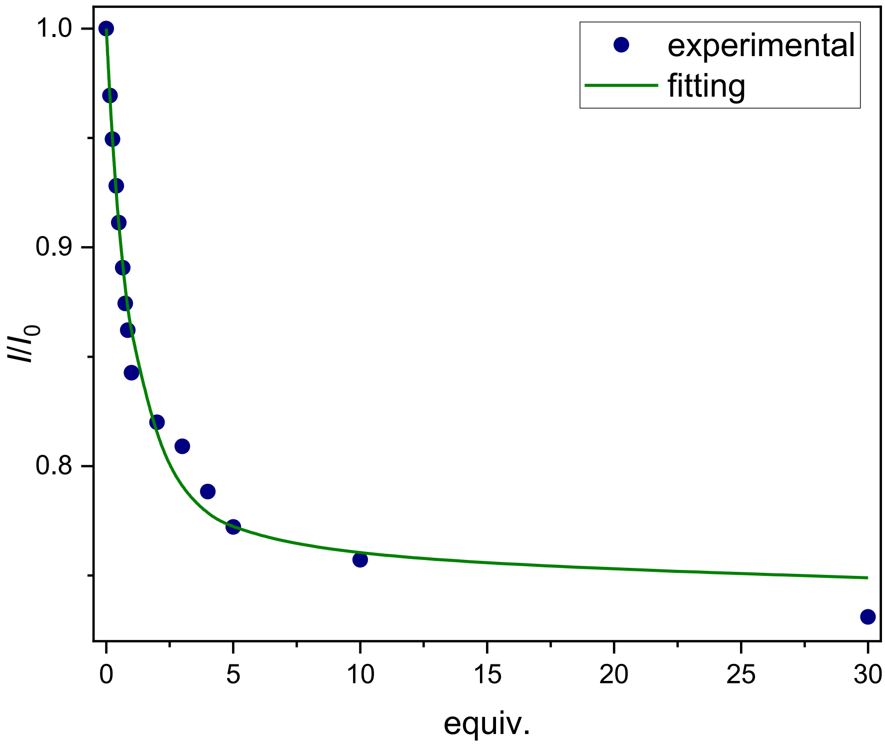

Figure S 69 Titration curve and global fitting (Bindfit) for interactions between **2** and **I**$^-$ in H_2O:THF = 95:5 *v/v* solvent system (model 2:1 statistical, Nelder-Mead method-algorithm, dilution correction, $K_a = (5.12\pm0.51)\cdot10^4$ M^{-1}, covariance = $1.50\cdot10^{-2}$).

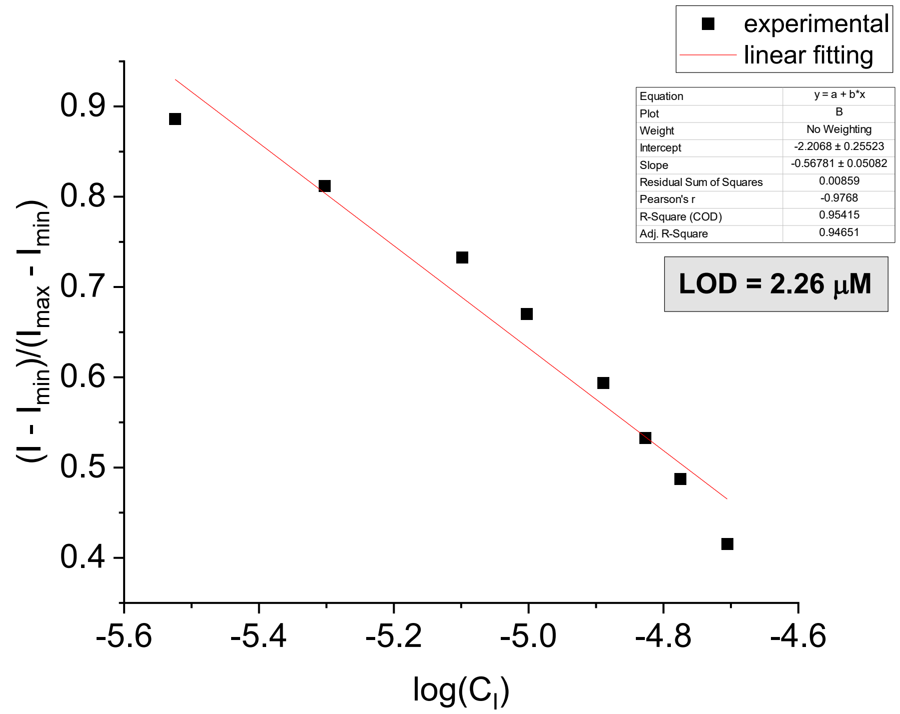

Figure S 70 (I-I_{min})/(I_{max}-I_{min}) versus log(C_{cation}) plot for the estimation of LOD for the interactions between **2** and **I**$^{-}$. The linear fit data and calculated LOD are presented in the figure.

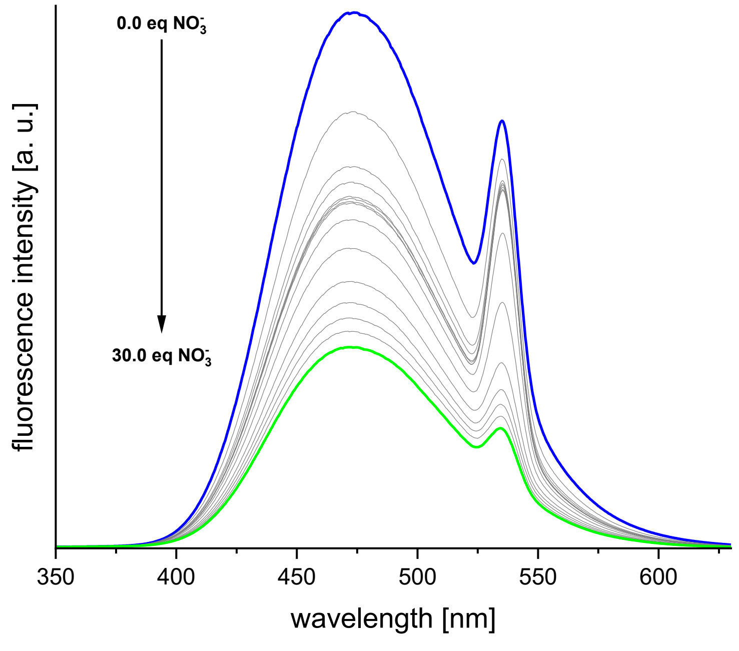

Figure S 71 Emission spectra of aggregated **2** in the presence of increasing molar equivalents of $\mathbf{NO_3}^{-}$. Conditions: H_2O:THF = 95:5 *v/v*, $C_I = 2{\cdot}10^{-5}$ M, λ_{ex} = 266 nm, λ_{em} = 475 nm.

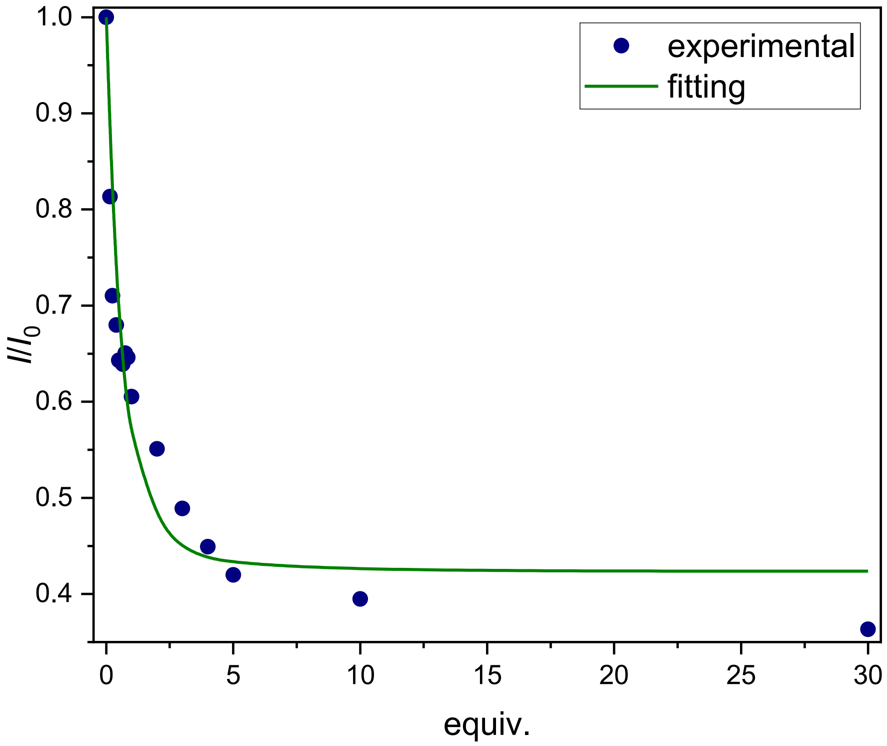

Figure S 72 Titration curve and global fitting (Bindfit) for interactions between **2** and **NO_3^-** in H_2O:THF = 95:5 *v/v* solvent system (model 2:1 statistical, Nelder-Mead method-algorithm, dilution correction, K_a = (9.56±2.29) $\cdot 10^4$ M^{-1}, covariance = $9.83 \cdot 10^{-2}$).

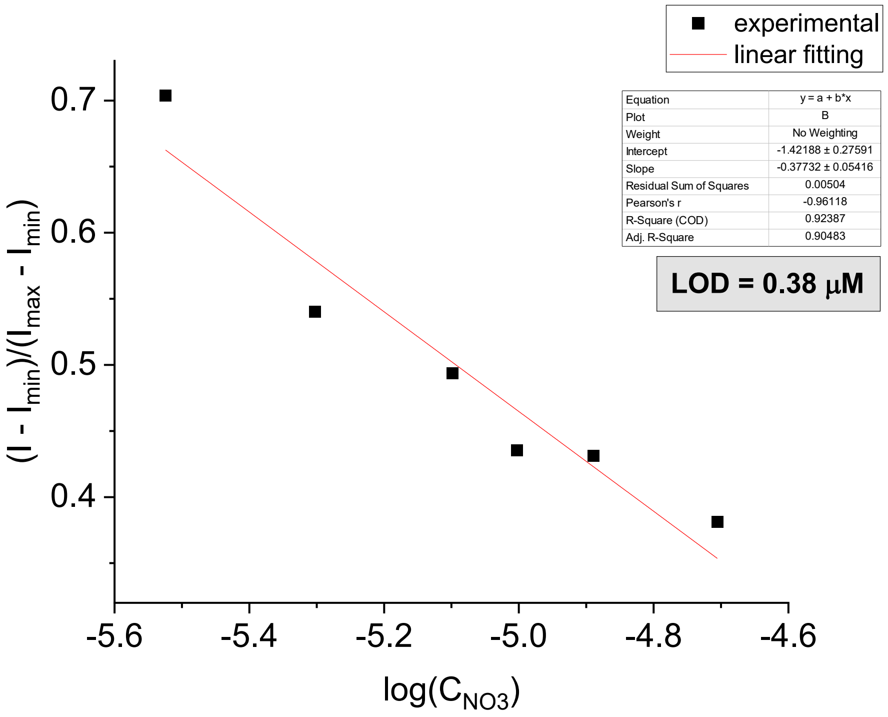

Figure S 73 (I-I_{min})/(I_{max}-I_{min}) versus log(C_{cation}) plot for the estimation of LOD for the interactions between **2** and **NO_3^-**. The linear fit data and calculated LOD are presented in the figure.

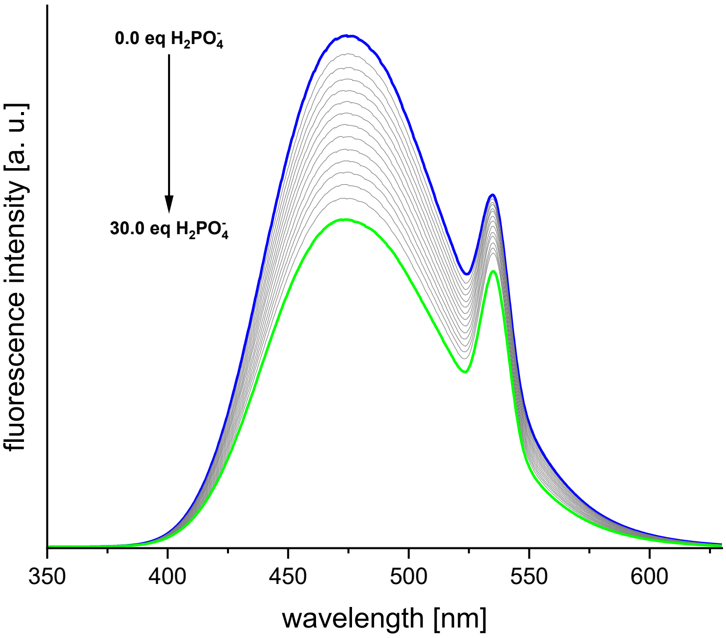

Figure S 74 Emission spectra of aggregated **2** in the presence of increasing molar equivalents of **$H_2PO_4^-$**. Conditions: H_2O:THF = 95:5 *v/v*, $C_1 = 2\cdot10^{-5}$ M, λ_{ex} = 266 nm, λ_{em} = 475 nm.

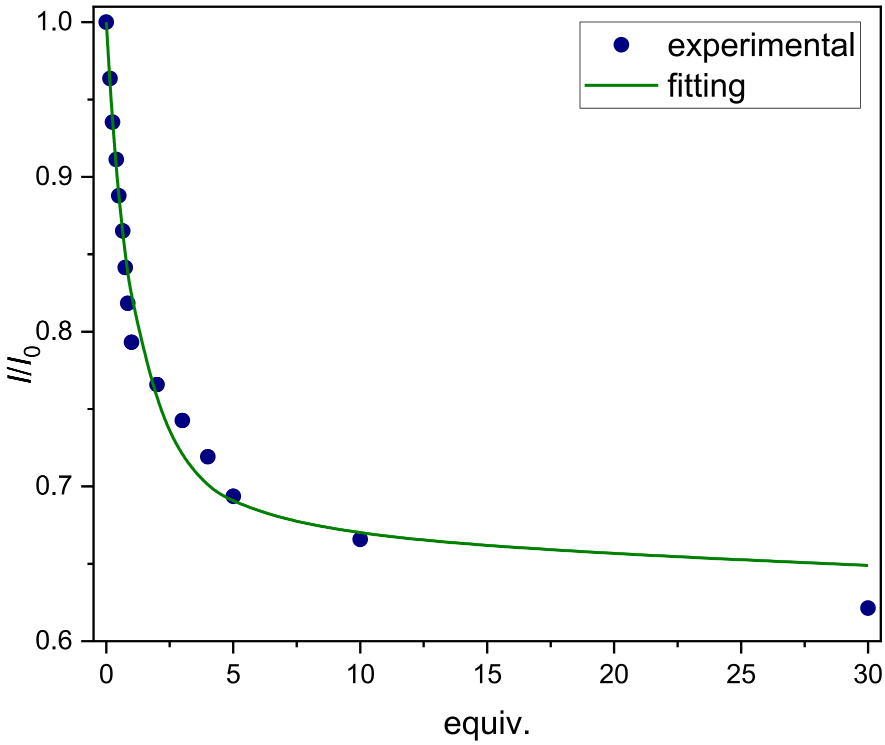

Figure S 75 Titration curve and global fitting (Bindfit) for interactions between **2** and **$H_2PO_4^-$** in H_2O:THF = 95:5 *v/v* solvent system (model 2:1 statistical, Nelder-Mead method-algorithm, dilution correction, K_a = $(4.33\pm0.48)\cdot10^4$ M^{-1}, covariance = $1.79\cdot10^{-2}$).

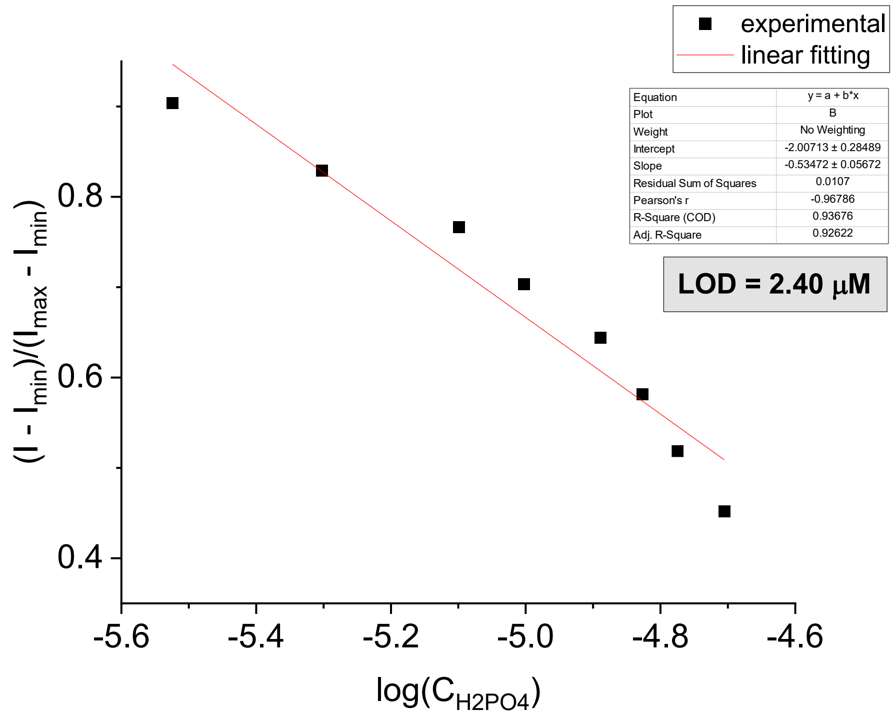

Figure S 76 (I-I_{min})/(I_{max}-I_{min}) versus log(C_{cation}) plot for the estimation of LOD for the interactions between **2** and **$H_2PO_4^-$**. The linear fit data and calculated LOD are presented in the figure.

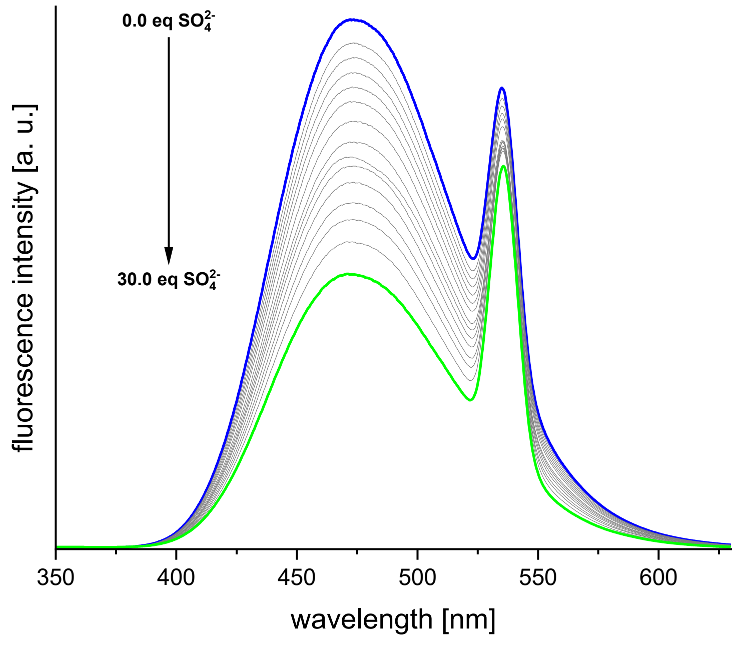

Figure S 77 Emission spectra of aggregated **2** in the presence of increasing molar equivalents of **SO_4^{2-}**. Conditions: H_2O:THF = 95:5 *v/v*, $C_1 = 2{\cdot}10^{-5}$ M, λ_{ex} = 266 nm, λ_{em} = 475 nm.

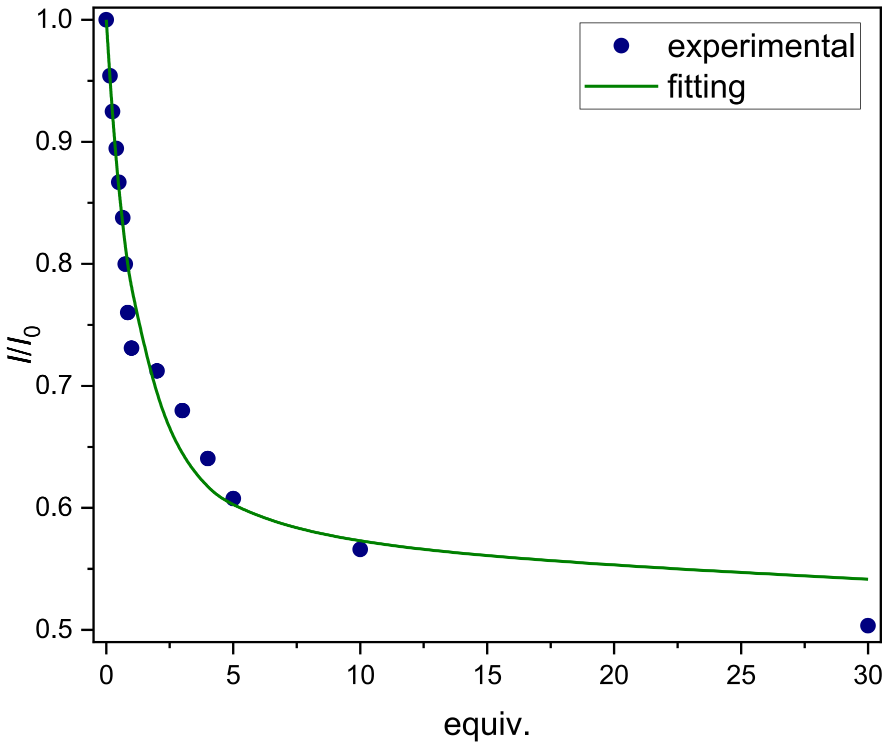

Figure S 78 Titration curve and global fitting (Bindfit) for interactions between **2** and $\mathbf{SO_4^{2-}}$ in H_2O:THF = 95:5 *v/v* solvent system (model 2:1 statistical, Nelder-Mead method-algorithm, dilution correction, K_a = (3.97±0.56)·10^4 M^{-1}, covariance = 2.71·10^{-2}).

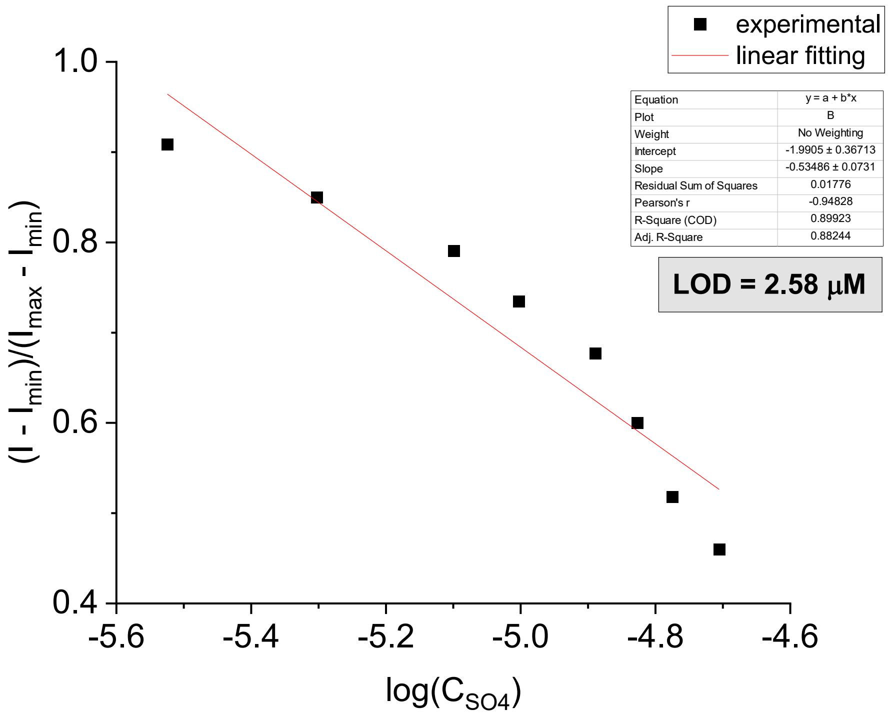

Figure S 79 (I-I_{min})/(I_{max}-I_{min}) versus log(C_{cation}) plot for the estimation of LOD for the interactions between **2** and $\mathbf{SO_4^{2-}}$. The linear fit data and calculated LOD are presented in the figure.

S7. Receptor studies – NMR

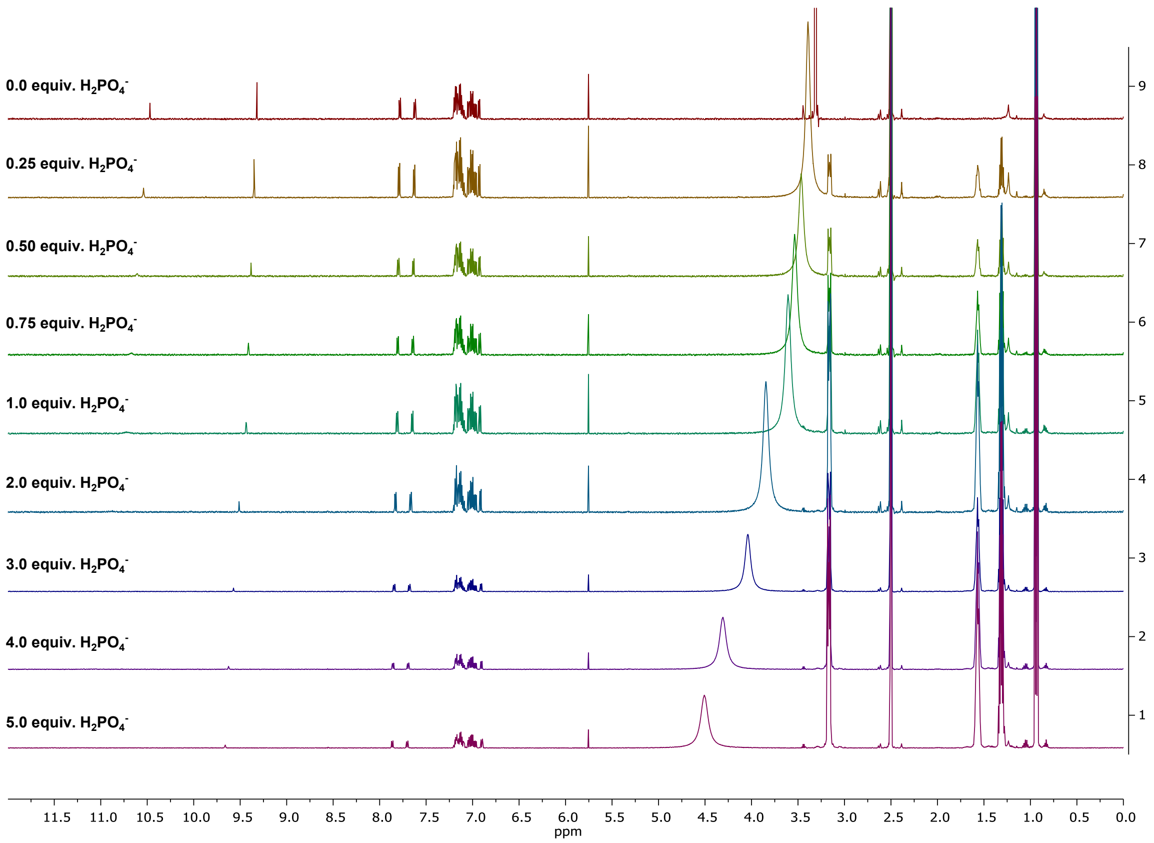

Figure S 80 Evolution of ^{1}H NMR spectra (600 MHz, DMSO-d_6 + TMS, C_1 = 1.29 mM) for the titration of **1** with $TBAH_2PO_4^-$:

S8. DLS

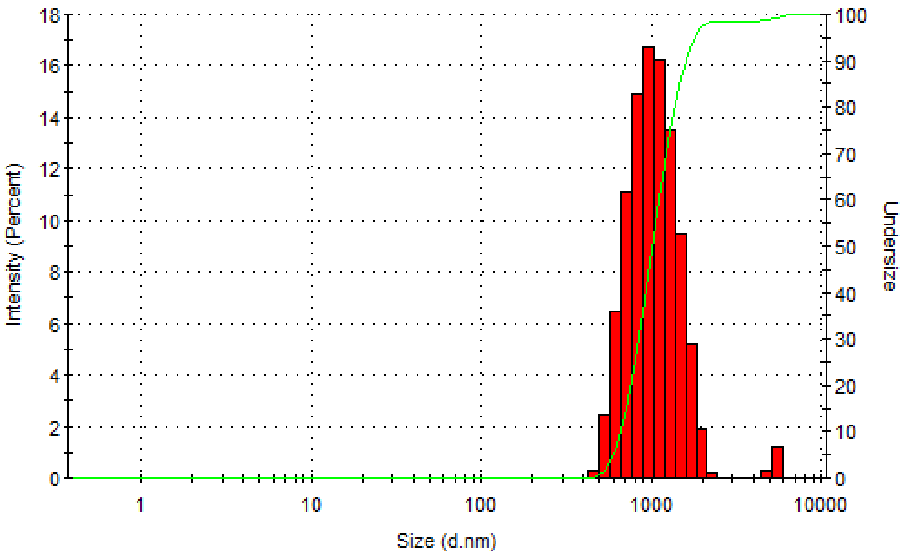

Figure S 81 Size distribution (DLS) for **1** in H_2O:THF = 95:5 *v/v* solvent system ($C = 2\cdot10^{-5}$ M)

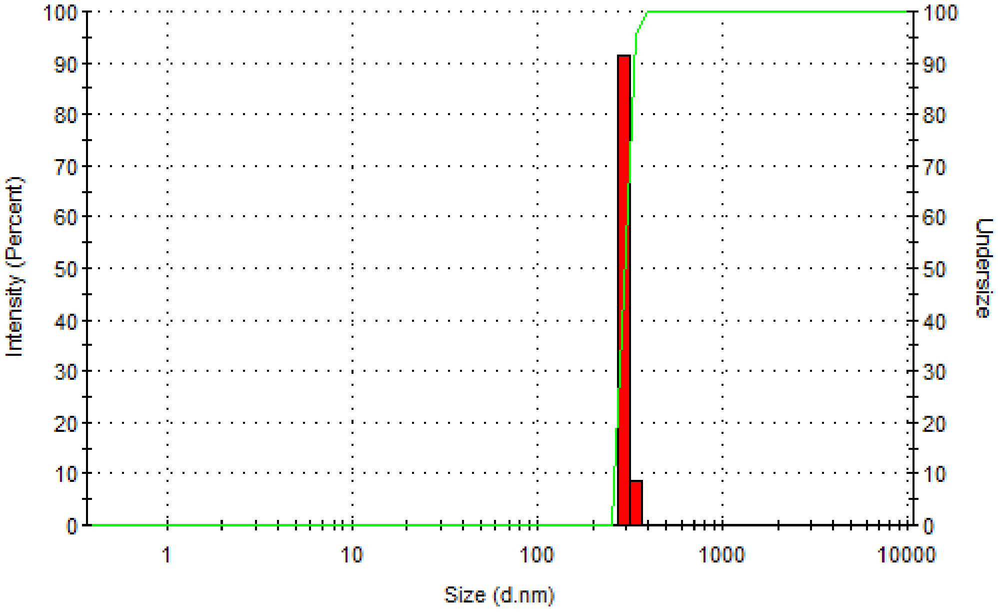

Figure S 82 Size distribution (DLS) for **1** in H_2O:THF = 95:5 *v/v* solvent system ($C = 2\cdot10^{-5}$ M) with addition of 5 equiv. of SO_4^{2-}

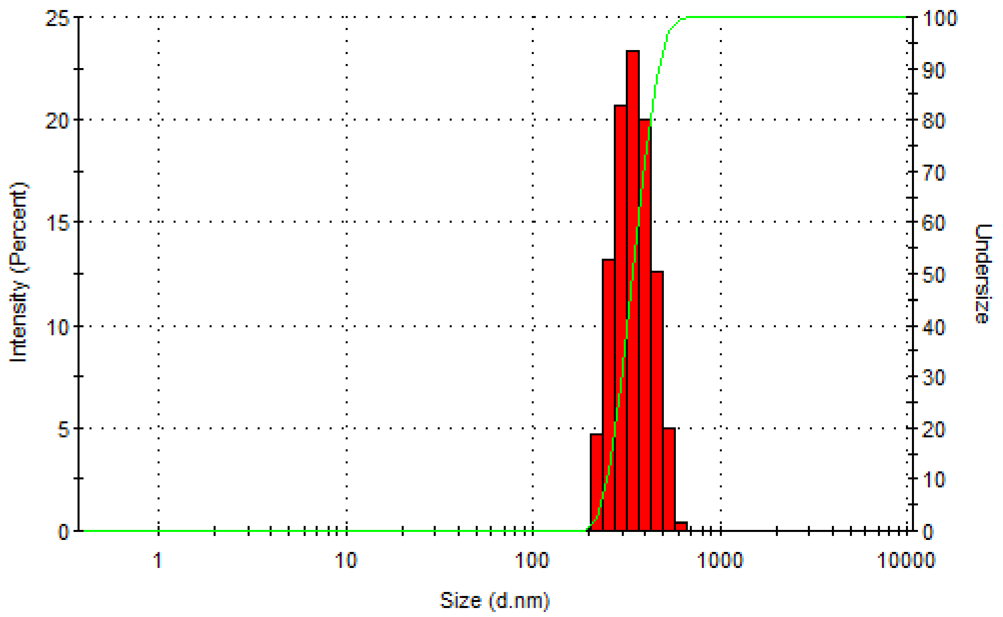

Figure S 83 Size distribution (DLS) for **2** in H_2O:THF = 95:5 *v/v* solvent system (C = $2 \cdot 10^{-5}$ M)

S9. supplementary references

(1) Bakker, E.; Bühlmann, P.; Pretsch, E. Carrier-Based Ion-Selective Electrodes and Bulk Optodes. 1. General Characteristics. *Chem. Rev.* **1997**, *97* (8), 3083–3132. https://doi.org/10.1021/cr940394a.

(2) Brouwer, A. M. Standards for Photoluminescence Quantum Yield Measurements in Solution (IUPAC Technical Report). *Pure Appl. Chem.* **2011**, *83* (12), 2213–2228. https://doi.org/10.1351/PAC-REP-10-09-31.

(3) Würth, C.; Grabolle, M.; Pauli, J.; Spieles, M.; Resch-Genger, U. Relative and Absolute Determination of Fluorescence Quantum Yields of Transparent Samples. *Nat. Protoc.* **2013**, *8* (8), 1535–1550. https://doi.org/10.1038/nprot.2013.087.

(4) Frisch, M. J.; Trucks, G. W.; Schlegel, H. B.; Scuseria, G. E.; Robb, M. A.; Cheeseman, J. R.; Scalmani, G.; Barone, V.; Petersson, G. A.; Nakatsuji, H.; Li, X.; Caricato, M.; Marenich, A. V.; Bloino, J.; Janesko, B. G.; Gomperts, R.; Mennucci, B.; Hratchian, H. P.; Ortiz, J. P.; Izmaylov, A. F.; Sonnenberg, L.; Williams-Young, D.; Ding, F.; Lipparini, F.; Egidi, F.; Peng, B.; Petrone, A.; Henderson, T.; Ranasinghe, D.; Zakrzewski, V. G.; Gao, J.; Rega, N.; Zheng, G.; Liang, W.; Hada, M.; Ehara, M.; Toyota, K.; Fukuda, R.; Hasegawa, J.; Hasegawa, J.; Ishida, M.; Nakajima, T.; Honda, Y.; Kitao, O.; Nakai, H.; Vreven, T.; Throssell, K.; Montgomery, Jr., J. A.; Peralta, J. E.; Ogliaro, F.; Bearpark, M. J.; Heyd, J. J.; Brothers, E. N.; Kudin, K. N.; Staroverov, V. N.; Keith, T. A.; Kobayashi, R.; Normand, J.; Raghavachari, K.; Rendell, A. P.; Burant, J. C.; Iyengar, S. S.; Tomasi, J.; Cossi, M.; Millam, J. M.; Klene, M.; Adamo, C.; Cammi, R.; Ochterski, J. W.; Martin, R. L.; Morokuma, K.; Farkas, O.; Foresman, J. Gaussian 16, Revision C.01, 2016.

(5) Becke, A. D. Density-functional Thermochemistry. III. The Role of Exact Exchange. *J. Chem. Phys.* **1993**, *98* (7), 5648–5652. https://doi.org/10.1063/1.464913.

(6) Binkley, J. S.; Pople, J. A.; Hehre, W. J. Self-Consistent Molecular Orbital Methods. 21. Small Split-Valence Basis Sets for First-Row Elements. *J. Am. Chem. Soc.* **1980**, *102* (3), 939–947. https://doi.org/10.1021/ja00523a008.

(7) Dennington, R., Keith, T.A. and Millam, J.M. (2016) GaussView 6.0. 16. Semichem Inc., Shawnee Mission. Scientific Research Publishing. https://www.scirp.org/reference/referencespapers?referenceid=3413212
